# Global proteomics of fibroblast cells treated with bacterial cyclic dinucleotides, c-di-GMP and c-di-AMP

**DOI:** 10.1080/20002297.2021.2003617

**Published:** 2021-12-29

**Authors:** Kenneth I. Onyedibe, Samira Elmanfi, Uma K. Aryal, Eija Könönen, Ulvi Kahraman Gürsoy, Herman O. Sintim

**Affiliations:** a Department of Chemistry, Purdue University, West Lafayette, USA; b Immunology and Infectious Disease, Purdue Institute for Drug Discovery and Purdue Institute of Inflammation, Purdue University, West Lafayette, USA; c Department of Periodontology, Institute of Dentistry, University of Turku, Turku, Finland; d Purdue Proteomics Facility, Bindley Bioscience Center, Purdue University, West Lafayette, USA; e Department of Comparative Pathobiology, Purdue University, West Lafayette, USA

**Keywords:** Cyclic dinucleotide, proteomics, fibroblasts

## Abstract

**Background:**

Constant exposure of human gingival fibroblasts (HGFs) to oral pathogens trigger selective immune responses. Recently, the activation of immune response to cyclic dinucleotides (CDNs) via STING has come to the forefront. Reports show that other proteins outside the STING-TBK1-IRF3 axis respond to CDNs but a global view of impacted proteome in diverse cells is lacking. HGFs are constantly exposed to bacterial-derived cyclic-di-adenosine monophosphate (c-di-AMP) and cyclic-di-guanosine monophosphate (c-di-GMP).

**AIM:**

To understand the response of HGFs to bacterial-derived CDNs, we carried out a global proteomics analysis of HGFs treated with c-di-AMP or c-di-GMP.

**Methods:**

The expression levels of several proteins modulated by CDNs were examined.

**Results:**

Interferon signaling proteins such as Ubiquitin-like protein ISG15 (ISG15), Interferon-induced GTP-binding protein Mx1 (MX1), Interferon-induced protein with tetratricopeptide repeats (IFIT) 1 (IFIT1), and (IFIT3) were significantly upregulated. Interestingly, other pathways not fully characterized to be regulated by CDNs, such as necroptosis signaling, iron homeostasis signaling, protein ubiquitination, EIF2 signaling, sumoylation and nucleotide excision repair pathways were also modulated by the bacterial-derived CDNs.

**Conclusion:**

This study has added to the increasing appreciation that beyond the regulation of cytokine production via STING, cyclic dinucleotides also broadly affect many critical processes in human cells.

## Introduction

**Figure 1.**
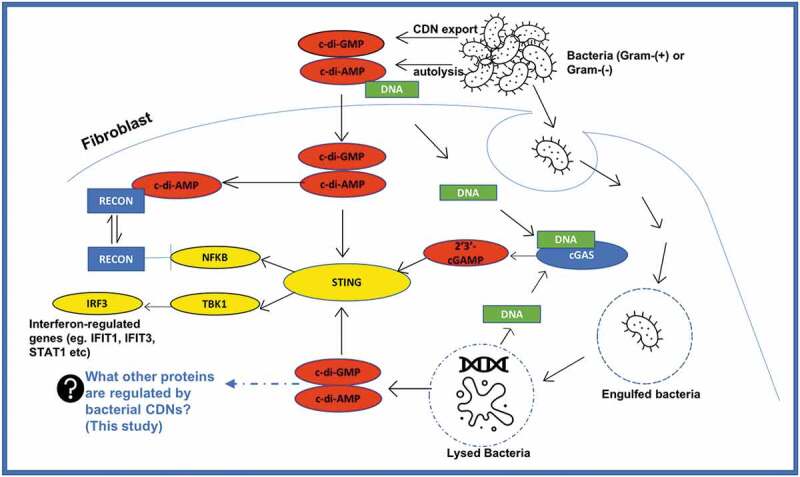
Current understanding of how cyclic dinucleotides promote inflammatory response via STING.

Cyclic dinucleotides (CDNs) are essential second messenger signaling molecules, which include cyclic-di-adenosine monophosphate (c-di-AMP), cyclic-di-guanosine monophosphate (c-di-GMP) and cyclic GMP-AMP (cGAMP). Several studies have described c-di-AMP and c-di-GMP secretion into the cytosol of mammalian host cells from both Gram-positive and Gram-negative bacterial pathogens during infections [1–4]. Production of these CDNs occur from a combination of two molecules of GTP or ATP in a reaction catalyzed by diguanylyl cyclases (DGC) for c-di-GMP or diadenylyl cyclases (DAC) for c-di-AMP [5]. C-di-AMP and c-di-GMP are bacterial signaling molecules and are described as pathogen associated molecular patterns (PAMPs) [5]. These CDNs have been reported to control several essential biochemical and physiological processes including virulence, immune system evasion, and survival mechanisms of pathogens and the converse protective immune responses in eukaryotic cells [3,5–7]. Thus, when pathogens invade or are engulfed by immune cells, the bacterial CDNs, which are released into the cytosol, activate signaling effector, a stimulator of interferon genes (STING), after which STING subsequently activates the TANK-binding kinase 1 (TBK1) – Interferon Regulatory Factor (IRF) 3 pathway. This leads to production of type 1 interferons and a robust innate immune response [8] (see Figure 1). The bacterial DNA, which is also released into the cytosol, activates cGAMP synthase (cGAS) to produce 2ʹ3’-cGAMP, which is even a better activator of STING than the bacterial-derived CDNs. The interferons produced and the resultant immune response keep the invading pathogen in check. Likewise, there are also several reports of immune response against viral infections and cancer cells via the STING/TBK1/IRF3 signaling pathway [9–11].

Accumulating evidences indicate that beyond upregulating the expression of interferons and cytokines via the STING pathway, CDNs also affect other non-STING pathways in a differential manner. For example, Woodward et al. demonstrated that c-di-AMP but not cGAMP binds to the oxidoreductase, RECON to regulate NF-kB [12]. cGAMP has also been shown to regulate ULK1 kinase via AMPK [13]. Using label-free quantitative proteomics, we previously demonstrated that c-di-GMP and 2ʹ3’cGAMP differentially affect pathways in macrophages [14]. As would be expected in macrophages, many of the proteins upregulated by the CDNs were related to cytokine signaling and interferon production including interferon-induced proteins 47, 202 and 204 (IFI47, IFI202, IFI204) and interferon-induced protein with tetratricopeptide repeats 1, 2 or 3 (IFIT1, IFIT2, IFIT3) [14]. Ubiquitin-like protein ISG15 (ISG15) was also significantly upregulated by cyclic dinucleotides [14]. The modulations of other processes by CDNs, other than inflammatory pathways, have not been fully characterized and this work aimed to fill this gap in knowledge, using fibroblast cell as a model.

The oral mucosa is home to numerous microorganisms, which potentially make the inflammatory response in that region very dynamic. In addition to immune cells, resident cells of gingiva, i.e. epithelial cells and fibroblasts, also come into contact with bacterial pathogens and respond to bacterial CDNs [7,15]. Oral fibroblasts are known for their role in periodontal wound healing and tissue remodeling [16]. Indeed, gingival fibroblasts constantly recognize bacterial virulence [17] and actively modulate immune response against it [18]. We previously demonstrated early and late cellular responses to CDNs by gingival epithelial and fibroblast cell lines [7,15]. In this report, we have extended the study to investigate the full proteome response when fibroblasts are treated with c-di-AMP and c-di-GMP using a global proteomics approach.

## Methods

In order to identify proteins and pathways affected by treatment with bacterial derived CDNs (c-di-AMP or c-di-GMP), we conducted a global proteomics study. Extracted wisdom teeth of five non-smoking, periodontally healthy young adults (age 18–25 year) were used to isolate human gingival fibroblast cells (HGFs). All patients gave informed consent before the surgical procedures. The Ethics committee of the Hospital District of South-West Finland and the Ethical committee of the Dentistry, University of Helsinki approved the experimental protocol (Permission date:19 November 2002, number of the study case: §262)). HGFs were grown in Dulbecco’s modified eagle medium (DMEM) supplemented with 10% fetal bovine serum (Gibco BRL, Life Technologies), antibiotics (100 IU/mL penicillin and 100 µg/mL streptomycin), and 1% non-essential amino acid (Gibco BRL, Life Technologies), at 37°C and 5% CO_2_.

HGFs (7 × 10^5^ /petri dish) were incubated at 37°C and 5% CO_2_ until the cells reached 80% confluency. The HGFs were incubated with fresh media containing 100 μM of c-di-GMP or 100 μM c-di-AMP at 37°C and 5% CO_2_ for 24 h. The control cells were not incubated with any CDN.

We then extracted proteins from the cell lines and performed global proteomics analyses at the Purdue Proteomics Facility as described previously [19]. Briefly, cells were lysed in 100 mM ammonium bicarbonate (ABC) using Barocycler (PBI Inc.). Proteins were then extracted by acetone precipitation and reconstituted back in 8 M urea for reduction, alkylation and trypsin/LysC digestion as described previously [20]. We collected LC-MS/MS data on a Thermo Q Exactive Orbitrap HF mass spectrometer coupled with a Dionex UltiMate 3000 HPLC system using a 120 min LC gradient and 300 μm ID × 5 mm trap column packed with 5 μm 100 Å PepMap C18 medium and 75 μm × 15 cm analytical column packed with 2 μm of 100 Å PepMap C18 medium (Thermo Fisher Scientific, Waltham, MA). Mobile phase A consisted of 0.1% FA in water, and mobile phase B consisted of 0.1% FA in 80% acetonitrile (ACN). The column temperature was maintained at 50°C. MS data were acquired with a Top 20 data-dependent MS/MS scan method with a maximum injection time of 100 m/s and a resolution of 120,000 at 200 m/z. Fragmentation of precursor ions was performed by high-energy C-trap dissociation (HCD) with a normalized collision energy of 27 eV. MS/MS scans were acquired at a resolution of 15,000 at m/z 200. The dynamic exclusion was set at 20 s to avoid repeated scanning of identical peptides. LC-MS/MS data were searched in MaxQuant (version 1.6.3.3) [21] against the Uniprot human protein database for protein identification and label-free quantitation (LFQ).

The Perseus software [22] was used for Maxquant data and bioinformatics analysis. For further analysis, we included only proteins that were identified in at least two out of the three treatment replicates and with at least two MS/MS counts. Label free quantification intensities were used to perform differential expression analysis. To determine differentially abundant proteins, LFQ intensities were Log2 transformed, data were filtered and Student’s t-test used with 5% permutation-based FDR except where otherwise mentioned. The R package of the MetaboAnalyst software Version 5.0 was used for the PLS-DA analysis and other statistical analyses when mentioned. Auto-scale normalized data were used to perform hierarchical clustering and to generate the heat map analysis in both Morpheus software and MetaboAnalyst. Scatter plots and correlation plots were used to determine the correlation between replicates. Venn diagrams were plotted using Venny software (Venny. 2.1). Volcano plots and PCA plots were generated in the OriginPro 2020 Software (OriginLab, MA). Pathway enrichment and graphics were carried out using the IPA functional network core analysis (QIAGEN Inc., https://www.qiagenbioinformatics.com/products/ingenuity-pathway-analysis).

## Results

### Global proteomics profile of fibroblasts in response to c-di-AMP and c-di-GMP

**Figure 2.**
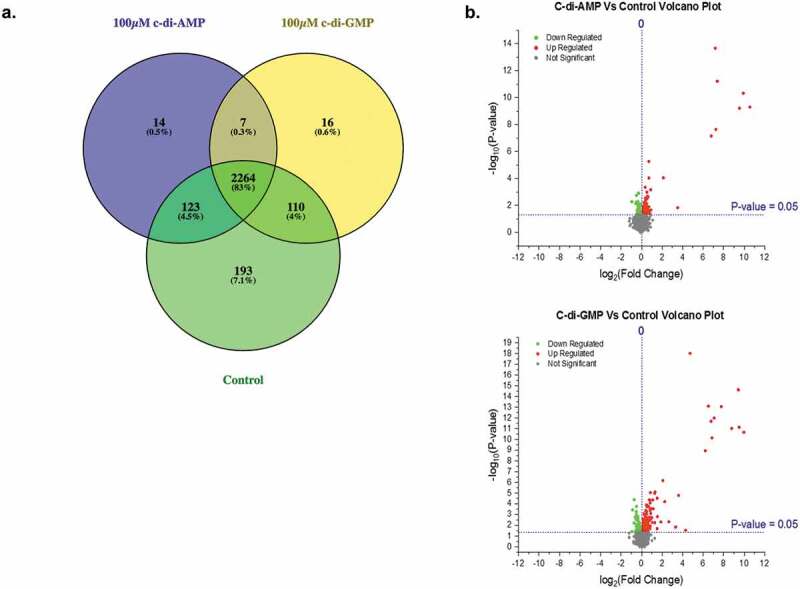
Global proteomic profiling. (**A)** Venn diagram showing the number and percentages of proteins identified in the control and 100 µM of c-di-AMP and c-di-GMP treated fibroblasts. Proteins in the subsets of the Venn diagram are listed in Table S1. (**B)** Volcano plots of quantified proteins in each treatment group versus controls. Horizontal blue line represents the Log10 (p value) cutoff. Volcano plots of c-di-AMP and c-di-GMP treatments showed significantly upregulated proteins (red dots) and downregulated proteins (green dots) in both treatment conditions. Volcano plots were plotted using the Origin (Pro), Version 2020 software (OriginLab Corporation, Northampton, MA).

We profiled the expression levels of the proteomes in c-di-AMP and c-di-GMP treated HGFs. While c-di-AMP and c-di-GMP are negatively charged and hence difficult to get into cells, earlier studies by us and also by others have shown that at high enough concentrations (such as 100 µM) some of the CDN get into the cell to affect the signaling pathway [14,15,23]. We also chose a 24 h time period for cell exposure to CDNs to mimic chronic infection whereby mammalian cells are constantly exposed to bacterial PAMPs for extended period. The analysis of the LC-MS/MS data identified a total of 3,810 proteins. Proteins that were detected in only one biological replicate were removed from the analysis. Thus, amongst proteins detected in at least two biological replicates, 2,690 proteins were identified in the control fibroblasts, whereas 2,407 and 2,397 proteins were identified in fibroblasts treated with 100 µM of c-di-AMP and c-di-GMP, respectively. About 83% of the total proteins identified were found in both treatment conditions and in the control fibroblasts whilst others were distributed differentially (see Figure 2a and supporting information (SI) Table S1). We observed a good correlation amongst the biological replicates using scatter plots (Figure S2A-C) as well as between samples using correlation plots (Figure S2D-E). Hierarchical clustering of proteins found in both control and CDN treated fibroblast samples revealed that the control and treatment samples clustered into three distinct groups (see SI, Figure S1). The Volcano plots showed that there were several regulated proteins at significant p values (< 0.05) (Figure 2b).

### Analysis of differentially expressed proteins in the different treatment groups

**Figure 3.**
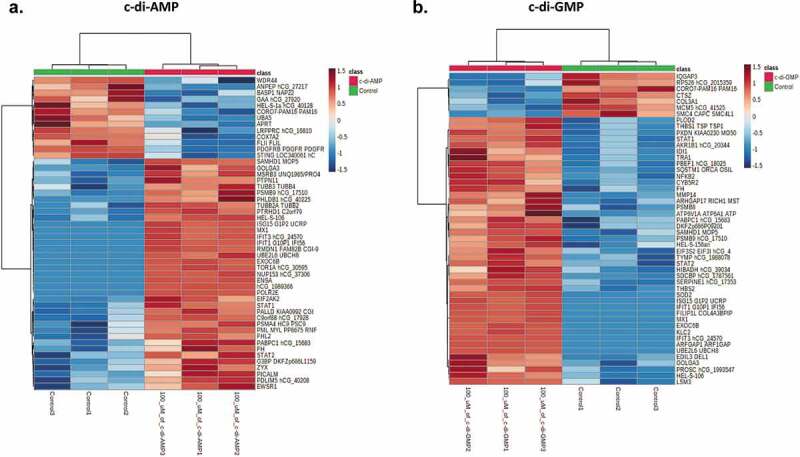
**(A)** Heatmap showing the top 50 regulated proteins (blue = downregulated, oxblood/red = upregulated) following c-di-AMP treatment. **(B)** Heatmap showing the top 50 regulated proteins following c-di-GMP treatment. Heatmaps were plotted on the MetaboAnalyst software Version 5.0 with auto-scale normalized data.

To determine the differential expression of proteins in the different treatment conditions, upregulated proteins (p ≤ 0.05 and Log2 fold change ≥ 0.5) and proteins that were undetectable in control fibroblasts but were seen in at least two biological replicates in each of the treatment conditions, were considered to be significantly upregulated by their respective treatment. A total of 46 proteins were significantly upregulated by c-di-AMP whilst 77 proteins were upregulated by c-di-GMP. Seventeen (16%) of these proteins were upregulated by both dinucleotides. Likewise, 50 (23.9%) of the total 209 significantly downregulated proteins were downregulated by both c-di-AMP and c-di-GMP. Additionally, we also identified the top 50 differentially expressed proteins by c-di-AMP (Figure 3a) and c-di-GMP (Figure 3b). Proteins which play major roles in interferon signaling and innate immune response were highly represented amongst the 17 commonly upregulated proteins (see Table S2). Important examples of the significantly upregulated innate immunity proteins include ISG15, Deoxynucleoside triphosphate triphosphohydrolase (SAMHD1), Signal transducer and activator of transcription 1-alpha/beta (STAT1), Signal transducer and activator of transcription (STAT2), MHC class I antigen (HLA-A), Interferon-induced GTP-binding protein Mx1 (MX1), Interferon-induced protein with tetratricopeptide repeats 1 (IFIT1), and Interferon-induced protein with tetratricopeptide repeats 3 (IFIT3). Immunoblotting indicated that both STING and TBK1 are expressed in the fibroblast cells (see SI, Figures S3 and S4), so it is not surprising to observe the upregulation of interferon-related proteins.

**Figure 4.**
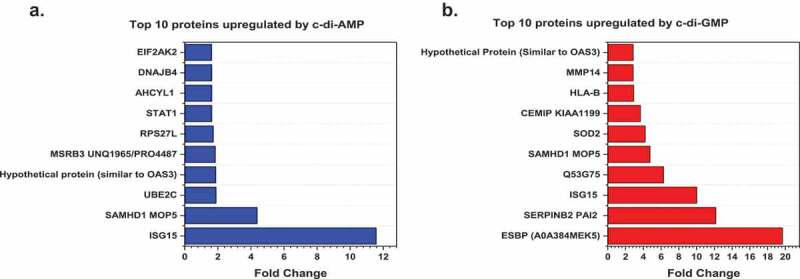
Top 10 proteins upregulated by c-di-AMP or c-di-GMP. (**A)** Top 10 statistically significant proteins with a measurable fold change, which are upregulated (blue bars) by c-di-AMP. **(B)** Top 10 statistically significant proteins with a measurable fold change, which are upregulated (red bars) by c-di-GMP. Charts were plotted using the Origin (Pro), Version 2020 software (OriginLab Corporation, Northampton, MA).

However, several other proteins essential in immune activation and pathogen killing were upregulated exclusively by either c-di-GMP or c-di-AMP. Notably, proteins such as Superoxide dismutase (SOD2), Immunity Related GTPase Q (IRGQ), Nuclear factor NF-kappa-B p100 subunit (NFKB2), Tumor necrosis factor receptor superfamily member 11B (TNFRSF11B), Beta-2-microglobulin (B2M), and Interferon-induced GTP-binding protein (MX2) were exclusively upregulated by c-di-GMP. Nevertheless, Interferon regulatory factor 9 (IRF9) and Interferon-induced 35 kDa protein (IFP 35) were exclusively upregulated by c-di-AMP. Interestingly, we observed that amongst the top 10 proteins significantly upregulated in each of the treatment conditions (Figures 4a and b), three proteins (ISG15, SAMHD1 and hypothetical OAS3) were commonly upregulated by both c-di-AMP and c-di-GMP. These were proteins with a measurable fold change compared to controls. For a complete list of all upregulated proteins in their various treatment groups, see supporting information Table S2. All significantly upregulated proteins with a measurable fold change are listed separately in Table S3 (c-di-AMP) and Table S4 (c-di-GMP).

Then, we further analyzed the downregulated proteins. In addition to differentially downregulated proteins with a measurable fold change (p ≤ 0.05 and Log2 fold change ≥ −0.5), proteins that were not detected in each of the treatment conditions but were detected in control fibroblasts were also considered to be significantly downregulated by the respective treatment, hence were undetectable. Amongst the 50 proteins downregulated in fibroblasts treated with both dinucleotides, Queuosine salvage protein, and E3 ubiquitin-protein ligase (MARCH5) were downregulated by both c-di-GMP and c-di-AMP. Another related E3 ubiquitination ligase, E3 ubiquitin-protein ligase RNF 181 (RNF181), was downregulated by only c-di-AMP. Similarly, some essential kinases such as Serine/threonine-protein kinase N2 (PKN2) and Uridine-cytidine kinase 2 (UCK2) were also downregulated by both c-di-GMP and c-di-AMP. Nevertheless, other critical kinases were downregulated by c-di-AMP and not c-di-GMP. For instance, Diacylglycerol kinase (DGK), Glycerol kinase (GK) and Mitogen-activated protein kinase (MAPK) were downregulated in the presence of only c-di-AMP. Conversely, a phosphatase, Glycerol-3-phosphate phosphatase (G3PP) and a phosphatase inhibitor, cAMP-regulated phosphoprotein 19 (ARPP19) were exclusively downregulated by c-di-GMP only. A complete list of proteins downregulated in the various treatment groups is provided in SI, Table S5. Downregulated proteins with a measurable fold change (p ≤ 0.05 and Log2 fold change ≥ −0.5) are listed separately with their fold change in Table S6 (c-di-AMP) and Table S7 (c-di-GMP).

### Multivariate analysis of proteins identified in both treatment conditions

**Figure 5.**
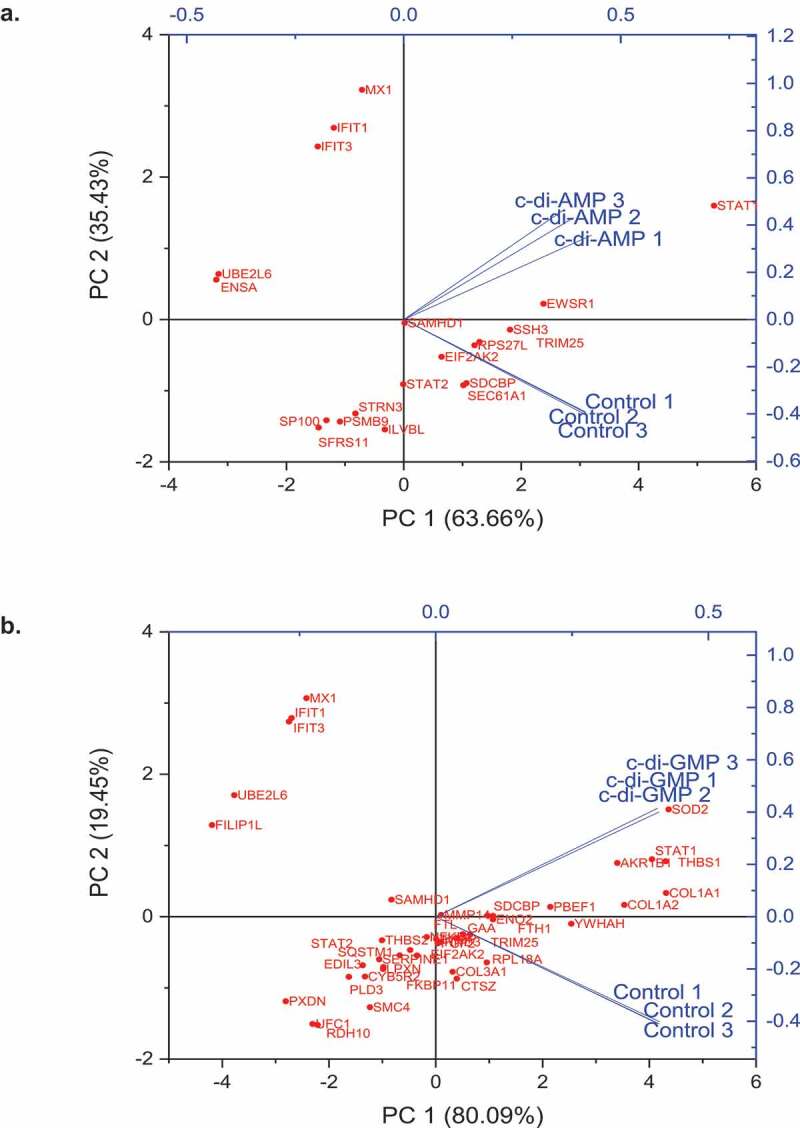
Multivariate analysis shows similar mapping of modulation of expressed proteins by both c-di-AMP and c-di-GMP. Four of the proteins (MX1, IFIT 1, IFIT3 and UBE2L6) seen in the upper left PC2 axis were also at the top of the VIP plots (Figure S6) and were the most statistically significant upregulated proteins (Figures S7 and S8). PCA analysis and biplots were created in the Origin (Pro), Version 2020 software (originlab corporation, northampton, MA).

Principal component analysis (PCA) was used to determine if the effects of c-di-GMP and c-di-AMP were different from control samples. PCA plots showing the relative mapping of all the significant proteins in treated and control samples are shown in supporting information Figure S5. For multivariate analysis, only proteins shown to be initially significant (Log2 fold change ≥ ± 0.5 and p ≤ 0.05) or exclusively identified in either control or treated HGFs were included. Principal component analysis biplots showed that principal components (PC) 1 and PC 2 accounted for over 99% of proteins that were significantly up- or down-regulated in both c-di-AMP and c-di-GMP treated fibroblasts (Figures 5a and b). Remarkably, in c-di-AMP and c-di-GMP biplots, there was a similar pattern of component mapping in the upper PC2 axis of significantly upregulated proteins (MX1, IFIT1, IFIT3, UBE2L6, SAMHD1). These proteins were amongst the 17 proteins commonly upregulated by both dinucleotides (Table S2). Conversely, Filamin A Interacting Protein 1 Like (FILIP1L) and SOD2, exclusively upregulated by c-di-GMP, were mapped in the upper PC2 and PC1 regions of the c-di-GMP biplot, respectively (Figure 5b). In addition, we analyzed the variable importance plots (VIP) scores of all upregulated and downregulated proteins following the treatments. VIP scores are based on the Partial least squares discriminant analysis (PLS-DA) model, which is a supervised classification method used to identify the relative importance of variables in proteomics or metabolomics data [24]. As expected, it was observed that proteins related to inflammation, including IFIT1, IFIT3, ISG15, UBE2L6, MX1, and STAT1, were similarly upregulated by both c-di-AMP and c-di-GMP (Figure S6). Four of these proteins (IFIT1, IFIT3, ISG15 and MX1) were amongst the topmost statistically significant proteins upregulated in both c-di-AMP and c-di-GMP treated HGFs by t-test (p < 0.0001) see Figures S7 and S8). In addition to the above four proteins, ENSA, SAMHD1, STAT1, SOD2, and FILIP1L seen in the PCA biplots were also prominentl at the top 50 c-di-AMP and c-di-GMP heatmaps (see Figures 3a and b).

### Functional analysis of proteins modulated by CDNs

**Figure 6.**
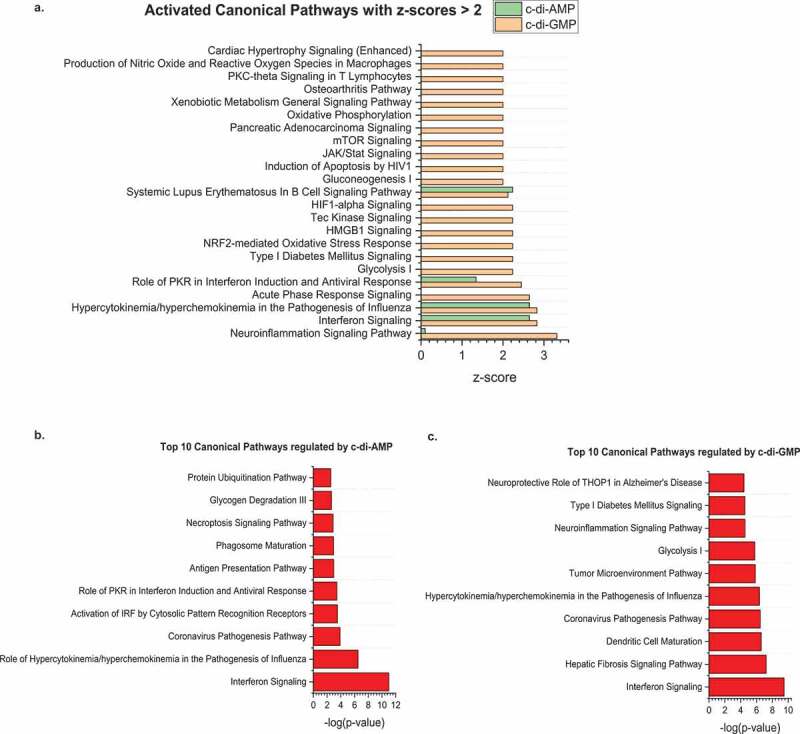
Enrichment analysis of ingenuity canonical pathways that are regulated by c-di-GMP and c-di-AMP. **(A)** Activated canonical pathways with z-scores ≥ 2. A positive z-score of 2 represents two standard deviations above the mean. **(B)** Top 10 ingenuity canonical pathways significantly regulated by c-di-AMP. **(C)** Top 10 ingenuity canonical pathways significantly regulated by c-di-GMP. The functional analyses and enrichment were generated by IPA (QIAGEN Inc., https://www.qiagenbioinformatics.com/products/ingenuity-pathway-analysis). p values which are represented as -log10 (p values) below the graph represents the magnitude of changes of the entire network of all identified proteins. Data were plotted using the Origin (Pro), Version 2020 software (OriginLab Corporation, Northampton, MA).

Furthermore, to illuminate the effects of c-di-AMP and c-di-GMP on various biological signaling pathways, a functional pathway analysis of the differentially expressed proteins in the different treatment groups was conducted using the ingenuity pathway analysis (IPA) functional network core analysis. A total of 150 ingenuity canonical pathways with a p value ≤ 0.05 were regulated by c-di-GMP in HGFs whilst 31 pathways were regulated by c-di-AMP. Amongst these signaling pathways, 21 pathways were regulated by both c-di-AMP and c-di-GMP (see Figure 6a and Table 1). In c-di-GMP and c-di-AMP treated HGFs, interferon signaling was the most significantly regulated canonical pathway with a -log p value of 9.49 and 11.0, respectively, and a positive activation z-score of greater than 2 in both instances. A positive z-score of 2 represents two standard deviations above the mean of the entire sample set. Other canonical pathways with a positive z-score ≥ 2 are shown in Figure 6a, whilst the top 10 most significantly regulated pathways (p < 0.05) for each treatment condition are shown in Figure 6b (c-di-AMP) and Figure 6c (c-di-GMP). Additionally, the graphical summary of each of the pathway analysis clearly demonstrates the important specific proteins involved in either activating (orange lines/shapes) or inhibiting (blue lines/shapes) various biological responses or pathways (Figures S9A and 9B). However, a deeper analysis of the interferon signaling network showed subtle differences between c-di-GMP and c-di-AMP (see Figures S10A and S10B). For instance, IRF9 played a major role in the c-di-AMP interferon signaling network but was not involved in the c-di-GMP network. Similarly, TAP1 featured prominently in the c-di-GMP Interferon signaling but did not play a visible role in the c-di-AMP network (see Figures S10A and S10B). Likewise, there were few differences in proteins involved in several other commonly regulated signaling pathways in the different CDNs (see Table 1).

**Table 1.** Pathways significantly regulated by c-di-AMP and c-di-GMP with -log p > 1.3 (-log p > 1.3 = p < 0.05)

S/No	Ingenuity Canonical Pathway	c-di-GMP (-log p-value)	c-di-GMP (z-score)	Molecules in c-di-GMP treated fibroblasts	c-di-AMP (-log p-value)	c-di-AMP (z-score)	Molecules in c-di-AMP treated fibroblasts
1	Interferon signaling	9.49	2.828	IFI35,IFIT1,IFIT3,ISG15,MX1,STAT1,STAT2,TAP1	11.0	2.646	IFI35,IFIT1,IFIT3,IRF9,ISG15,MX1,STAT1,STAT2
2	Hypercytokinemia/hyperchemokinemia in the pathogenesis of influenza	6.39	2.828	EIF2AK2,IFIT3,ISG15,MX1,NFKB2,PYCARD, STAT1,STAT2	6.52	2.646	EIF2AK2,IFIT3,IRF9,ISG15,MX1,STAT1,STAT2
3	Coronavirus pathogenesis pathway	6.48	1.265	ACE,CASP3,FAU,NFKB2,PYCARD,RPS26,SERPINE1,STAT1,STAT2, TRIM25	3.9	−1.633	IRF9,RPS26,RPS27L,STAT1,STAT2,TRIM25
4	Activation of IRF by Cytosolic Pattern Recognition Receptors	2.81	1	ISG15,NFKB2,STAT1, STAT2	3.51	1	IRF9,ISG15,STAT1, STAT2
5	Role of PKR in interferon induction and antiviral response	4.37	2.449	BID,CASP3,EIF2AK2,NFKB2,PYCARD,STAT1, STAT2	3.44	1.342	EIF2AK2,IRF9,PDGFRB,STAT1,STAT2
6	Antigen presentation pathway	3.61	N	HLA-A,HLA-B,PSMB9,TAP1	2.98	N	CANX,HLA-B,PSMB9
7	Phagosome maturation	2.89		CTSZ,HLA-A,HLA-B,RAB7A,TAP1,TUBG1	2.96	N	ATP6V0C,CANX,DCTN4,HLA-B,VPS18
8	Necroptosis signaling pathway	2.8	1.633	EIF2AK2,PYCARD,STAT1,STAT2,TNFRSF11B, TOMM20	2.88	1.342	EIF2AK2,IRF9,STAT1,STAT2,TOMM20
9	Glycogen degradation III	2.28	N	GAA,TYMP	2.64	N	GAA,TYMP
10	Protein ubiquitination pathway	2.22	N	HLA-A,HLA-B, PSMB9,PSME2,TAP1, UBE2L6,UCHL5	2.54	N	HLA-B, PSMB9, PSME2, THOP1,UBE2C,UBE2L6
11	EIF2 signaling	2.69	−0.447	EIF2AK2,FAU,RALB,RPL18,RPL18A,RPL7,RPS26	2.22	N	EIF2AK2,EIF2S3, RPLP1,RPS26,RPS27L
12	Role of JAK1, JAK2 and TYK2 in interferon signaling	3.06	N	NFKB2,STAT1,STAT2	2.18	N	STAT1,STAT2
13	Insulin secretion signaling pathway	1.35	0.447	DLAT,PLCB4,SEC61A1, STAT1,STAT2	2.07	0.447	EIF2S3,SEC61A1,SSR4,STAT1,STAT2
14	PDGF signaling	1.51	N	EIF2AK2,RALB,STAT1	2.01	N	EIF2AK2,PDGFRB, STAT1
15	T cell exhaustion signaling pathway	1.9	N	HLA-A,HLA-B, RALB,STAT1,STAT2	1.90	N	HLA-B, IRF9, STAT1,STAT2
16	Systemic Lupus Erythematosus in B cell signaling pathway	2.8	2.121	IFIT3,IL6ST,ISG15,MAP4K4,NFKB2,RALB,STAT1,STAT2	1.86	2.236	IFIT3,IRF9,ISG15,STAT1,STAT2
17	Sumoylation pathway	2.04	N	NFKB2,PML,RND3, SP100	1.80	N	PML,RFC2,SP100
18	NER pathway	1.32	N	CETN2,POLR2A,TOP2A	1.80	N	CETN2,POLR2A,RFC2
19	Heme degradation	1.51	N	HMOX1	1.69	N	HMOX1
20	Eumelanin biosynthesis	1.41	N	DDT	1.60	N	MIF
21	Iron homeostasis signaling pathway	2.33	N	ACO1,FTH1,FTL,HMOX1,TFRC	1.47	N	ATP6V0C,HMOX1, PDGFRB

*N = z-score was not determined/indeterminate by IPA

## Discussion

Our findings clearly revealed that proteins that play a major role in interferon signaling and innate immune responses were upregulated in CDN stimulated human gingival fibroblasts (Table S2 and Figure 5). For instance, STAT1, a major transcription activator for several innate and adaptive immune processes, was upregulated by the CDNs [25]. Inflammatory cytokines such as (TNF-α) activate upregulation of STAT1 gene expression in gingival fibroblasts [26] and inhibition of STAT1 decreases macrophage infiltration [27]. Other proteins such as ISG15 and SAMHD1, which are key members of the interferon production network, were also upregulated by several folds. NFKB2, although upregulated by only c-di-GMP, plays an essential role in the NF-κB pathway [28]. Activation of the NF-κB pathway by inflammatory mediators (proinflammatory cytokines) or by periodontitis-associated bacteria (*Fusobacterium nucleatum, Porphyromonas gingivalis*) leads to transcriptional induction of inflammatory cytokines and other mediators in gingival fibroblasts [28]. The preponderance of interferon and cytokine signaling in CDN treated HGFs is expected as we showed that STING and TBK1 were expressed in fibroblasts (see SI, Figures S3 and S4) and it has already been established by others that bacterial CDNs activate STING [3,29,30]. Consequently, several studies have suggested the use of CDNs as vaccine adjuvants because of these immunostimulatory effects [31–34]. SERPINEB2, a member of the clade B family of serine protease inhibitors was the second most upregulated protein (12 folds) following the exposure to c-di-GMP. SERPINB2 has cytoprotective properties in cells and interacts with several components of the ubiquitin-proteasome system [35,36]. Lee et al. [36] showed that knockout of SERPINB2 led to significant reduction in the activity of the ubiquitin-proteasome system in addition to dysregulated autophagy when compared to wild-type cells [36]. Moreover, studies have also shown that SERPINB2 regulates TH1/TH2 adaptive immune response in inflammatory [37], viral [38], and parasitic infections [39]. Furthermore, Neilands et al. [40] showed that SERPINB2 specifically inhibited the proteolytic activity of *P. gingivalis*, a key periodontal pathogen. Further studies are required to determine the specific mechanisms behind the proteasome and immune modulation function of SERPINB2. This work also indicated that CDNs regulate DGK and PKN2. While this study used fibroblasts and one cannot readily extrapolate the results to other cell types, it has been shown that DGKs, which convert diacylglycerol (DAG) to phosphatidic acid, modulate T helper cell differentiation in a dose dependent manner [41]. Further studies, using other cell types, such as T-cells, are warranted to decipher if DGKs are modulated by CDNs in different cell types.

The signaling pathways influenced by the CDN treatments were revealing. Essentially, 13 out of 21 (~62%) of the signaling pathways regulated in HGFs exposed to bacterial derived CDNs, were immunity related pathways (Table 1). Additionally, the antigen presentation pathway regulated by both CDNs, is in keeping with upregulation of HLA proteins and the downregulation of DGK, which play crucial roles in T cell modulation [41,42].

In conclusion, our results indicated that in addition to the regulation of interferon signaling and innate immune responses of human gingival fibroblasts by bacterial CDNs (expected for PAMPs), these signaling molecules also regulate other processes, such as necroptosis signaling, iron homeostasis signaling, protein ubiquitination, EIF2 signaling, sumoylation and nucleotide excision repair pathways.

It is therefore clear that cyclic dinucleotides play more extensive regulatory roles in mammalian cells than currently characterized and we hope that the scientific community will help elucidate the details of how CDNs affect these numerous pathways, which is obviously an endeavor too big to be undertaken by a single laboratory.

## Supplementary Material

Supplemental Material

## Data Availability

All raw LC-MS/MS data can be found in the Mass Spectrometry Interactive Virtual Environment (http://massive.ucsd.edu) with the ID: MSV000087090/

## References

[1] Woodward JJ, Iavarone AT, Portnoy DA. c-di-AMP secreted by intracellular *Listeria monocytogenes* activates a host type I interferon response. Science. 2010;328(5986):1703–10. 20508090 10.1126/science.1189801 PMC3156580

[2] Corrigan RM, Abbott JC, Burhenne H, et al. c-di-AMP is a new second messenger in staphylococcus aureus with a role in controlling cell size and envelope stress. PLoS Pathog. 2011 Sep;7(9):e1002217. 21909268 10.1371/journal.ppat.1002217 PMC3164647

[3] Barker JR, Koestler BJ, Carpenter VK, et al. STING-dependent recognition of cyclic di-AMP mediates type I interferon responses during Chlamydia trachomatis infection. MBio. 2013;4(3):e00018–13. 23631912 10.1128/mBio.00018-13 PMC3663186

[4] Kamegaya T, Kuroda K, Hayakawa Y. Identification of a Streptococcus pyogenes SF370 gene involved in production of c-di-AMP. Nagoya J Med Sci. 2011 Feb;73(1–2):49–57. 21614937 PMC11254362

[5] Krasteva PV, Sondermann H. Versatile modes of cellular regulation via cyclic dinucleotides. Nat Chem Biol. 2017;13(4):350–359. 03. 28328921 10.1038/nchembio.2337 PMC5773344

[6] Gursoy UK, Gürsoy M, Könönen E, et al. Cyclic dinucleotides in oral bacteria and in oral biofilms. Front Cell Infect Microbiol. 2017;7:273. 28680857 10.3389/fcimb.2017.00273 PMC5478684

[7] Elmanfi S, Zhou J, Sintim HO, et al. Regulation of gingival epithelial cytokine response by bacterial cyclic dinucleotides. J Oral Microbiol. 2019;11(1):1538927. 30598733 10.1080/20002297.2018.1538927 PMC6263105

[8] Sun L, Wu J, Du F, et al. Cyclic GMP-AMP synthase is a cytosolic DNA sensor that activates the type I interferon pathway. Science. 2013;339(6121):786–791. 23258413 10.1126/science.1232458 PMC3863629

[9] Sun L, Xing Y, Chen X, et al. Coronavirus papain-like proteases negatively regulate antiviral innate immune response through disruption of STING-mediated signaling. PLoS One. 2012;7(2):e30802. 22312431 10.1371/journal.pone.0030802 PMC3270028

[10] Li XD, Wu J, Gao D, et al. Pivotal roles of cGAS-cGAMP signaling in antiviral defense and immune adjuvant effects. Science. 2013;341(6152):1390–1394. 23989956 10.1126/science.1244040 PMC3863637

[11] Zhong B, Yang Y, Li S, et al. The adaptor protein MITA links virus-sensing receptors to IRF3 transcription factor activation. Immunity. 2008;29(4):538–550. 18818105 10.1016/j.immuni.2008.09.003

[12] McFarland AP, Luo S, Ahmed-Qadri F, et al. Sensing of bacterial cyclic dinucleotides by the oxidoreductase RECON promotes NF-κB activation and shapes a proinflammatory antibacterial state. Immunity. 2017;46(3):433–445. 3 21. 28329705 10.1016/j.immuni.2017.02.014 PMC5404390

[13] Prantner D, Perkins DJ, Vogel SN. AMP-activated kinase (AMPK) promotes innate immunity and antiviral defense through modulation of stimulator of interferon genes (STING) signaling. J Biol Chem. 2017;292(1):292–304. 27879319 10.1074/jbc.M116.763268 PMC5217687

[14] Sooreshjani MA, Gursoy UK, Aryal UK,et al. Proteomic analysis of RAW macrophages treated with cGAMP or c-di-GMP reveals differentially activated cellular pathways. RSC Adv. 2018;8:36840–36851. 10.1039/c8ra04603d PMC9089301 35558957

[15] Elmanfi S, Sintim HO, Zhou J, et al. Activation of gingival fibroblasts by bacterial cyclic dinucleotides and lipopolysaccharide. Pathogens. 2020;9(10):792. 10.3390/pathogens9100792 PMC7600373 32993127

[16] Smith PC, Martínez C, Martínez J, et al. Role of fibroblast populations in periodontal wound healing and tissue remodeling. Front Physiol. 2019;10:270. 31068825 10.3389/fphys.2019.00270 PMC6491628

[17] Uehara A, Takada H. Functional TLRs and NODs in human gingival fibroblasts. J Dent Res. 2007;86(3):249–254. 17314257 10.1177/154405910708600310

[18] Tzach-Nahman R, Nashef R, Fleissig O, et al. Oral fibroblasts modulate the macrophage response to bacterial challenge. Sci Rep. 2017;7(1):11516. 09. 28912533 10.1038/s41598-017-11771-3 PMC5599598

[19] Aryal UK, Hedrick V, Onyedibe KI, et al. Global proteomic analyses of STING-positive and -negative macrophages reveal STING and Non-STING differentially regulated cellular and molecular pathways. Proteomics Clin Appl. 2020;14(3):e1900109. 32065729 10.1002/prca.201900109

[20] Opoku-Temeng C, Onyedibe KI, Aryal UK, et al. Proteomic analysis of bacterial response to a 4-hydroxybenzylidene indolinone compound, which re-sensitizes bacteria to traditional antibiotics. J Proteomics. 2019 06;202:103368. 31028946 10.1016/j.jprot.2019.04.018

[21] Cox J, Mann M. MaxQuant enables high peptide identification rates, individualized p.p.b.-range mass accuracies and proteome-wide protein quantification. Nat Biotechnol. 2008;26(12):1367–1372. 19029910 10.1038/nbt.1511

[22] Tyanova S, Temu T, Sinitcyn P, et al. The perseus computational platform for comprehensive analysis of (prote)omics data. Nat Methods. 2016;13(9):731–740. 27348712 10.1038/nmeth.3901

[23] Chang D, Whiteley AT, Bugda Gwilt K, et al. Extracellular cyclic dinucleotides induce polarized responses in barrier epithelial cells by adenosine signaling. Proc Natl Acad Sci U S A. 2020;117(44):27502–27508. 11 03. 33087577 10.1073/pnas.2015919117 PMC7959571

[24] Karp NA, Griffin JL, Lilley KS. Application of partial least squares discriminant analysis to two-dimensional difference gel studies in expression proteomics. Proteomics. 2005;5(1):81–90. 15744836 10.1002/pmic.200400881

[25] Mogensen TH. IRF and STAT transcription factors - from basic biology to roles in infection, protective immunity, and primary immunodeficiencies. Front Immunol. 2018;9:3047. 30671054 10.3389/fimmu.2018.03047 PMC6331453

[26] Davanian H, Båge T, Lindberg J, et al. Signaling pathways involved in the regulation of TNFα-induced toll-like receptor 2 expression in human gingival fibroblasts. Cytokine. 2012;57(3):406–416. 22227093 10.1016/j.cyto.2011.12.008

[27] Wei W, Xiao X, Li J, et al. Activation of the STAT1 pathway accelerates periodontitis in. J Dent Res. 2019;98(9):1027–1036. 08. 31329047 10.1177/0022034519858063 PMC6651763

[28] Liu T, Zhang L, Joo D, et al. NF-κB signaling in inflammation. Signal Transduct Target Ther. 2017;2(1):17023. 10.1038/sigtrans.2017.23 PMC5661633 29158945

[29] Morehouse BR, Govande AA, Millman A, et al. STING cyclic dinucleotide sensing originated in bacteria. Nature. 2020;586(7829):429–433. 10. 32877915 10.1038/s41586-020-2719-5 PMC7572726

[30] Burdette DL, Monroe KM, Sotelo-Troha K, et al. STING is a direct innate immune sensor of cyclic di-GMP. Nature. 2011;478(7370):515–518. 21947006 10.1038/nature10429 PMC3203314

[31] Karaolis DK, Means TK, Yang D, et al. Bacterial c-di-GMP is an immunostimulatory molecule. J Immunol. 2007;178(4):2171–2181. 17277122 10.4049/jimmunol.178.4.2171

[32] Gogoi H, Mansouri S, Jin L. The age of cyclic dinucleotide vaccine adjuvants. Vaccines (Basel). 2020;8(3):453. 10.3390/vaccines8030453 PMC7563944 32823563

[33] Chandra D, Quispe-Tintaya W, Jahangir A, et al. STING ligand c-di-GMP improves cancer vaccination against metastatic breast cancer. Cancer Immunol Res. 2014;2(9):901–910. 24913717 10.1158/2326-6066.CIR-13-0123 PMC4264585

[34] Sintim HO, Mikek CG, Wang M, et al. Interrupting cyclic dinucleotide-cGAS-STING axis with small molecules. Medchemcomm. 2019;10(12):1999–2023. 32206239 10.1039/c8md00555a PMC7069516

[35] Akiyama H, Ikeda K, Kondo H, et al. Microglia express the type 2 plasminogen activator inhibitor in the brain of control subjects and patients with Alzheimer’s disease. Neurosci Lett. 1993;164(1–2):233–235. 8152607 10.1016/0304-3940(93)90899-v

[36] Lee JA, Yerbury JJ, Farrawell N, et al. SerpinB2 (PAI-2) modulates proteostasis via binding misfolded proteins and promotion of cytoprotective inclusion formation. PLoS One. 2015;10(6):e0130136. 26083412 10.1371/journal.pone.0130136 PMC4470917

[37] Schroder WA, Le TT, Major L, et al. A physiological function of inflammation-associated SerpinB2 is regulation of adaptive immunity. J Immunol. 2010;184(5):2663–2670. 20130210 10.4049/jimmunol.0902187

[38] Major LD, Partridge TS, Gardner J, et al. Induction of SerpinB2 and Th1/Th2 modulation by SerpinB2 during lentiviral infections in vivo. PLoS One. 2013;8(2):e57343. 23460840 10.1371/journal.pone.0057343 PMC3583835

[39] Schroder WA, Gardner J, Le TT, et al. SerpinB2 deficiency modulates Th1⁄Th2 responses after schistosome infection. Parasite Immunol. 2010;32(11–12):764–768. 21086717 10.1111/j.1365-3024.2010.01241.x

[40] Neilands J, Bikker FJ, Kinnby B. PAI-2/SerpinB2 inhibits proteolytic activity in a P. gingivalis-dominated multispecies bacterial consortium. Arch Oral Biol. 2016;70:1–8. 27295389 10.1016/j.archoralbio.2016.05.016

[41] Yang J, Wang HX, Xie J, et al. DGK α and ζ Activities Control T. Front Immunol. 2019;10:3048. 32010133 10.3389/fimmu.2019.03048 PMC6974463

[42] Jung IY, Kim YY, Yu HS, et al. CRISPR/Cas9-mediated knockout of DGK improves antitumor activities of human T cells. Cancer Res. 2018;78(16):4692–4703. 08 29967261 10.1158/0008-5472.CAN-18-0030

