# Supplementary material for: Global proteomics of fibroblast cells treated with bacterial cyclic dinucleotides, c-di-GMP and c-di-AMP
Source: J Oral Microbiol. 2021 Dec 29;14(1):2003617. doi: 10.1080/20002297.2021.2003617 (PMC8725719; doi:10.1080/20002297.2021.2003617)
Supplement: Supplemental Material [file ZJOM_A_2003617_SM5937.docx]

**Global proteomics of fibroblast cells treated with bacterial cyclic dinucleotides, c-di-GMP and c-di-AMP**

**Supporting Information**


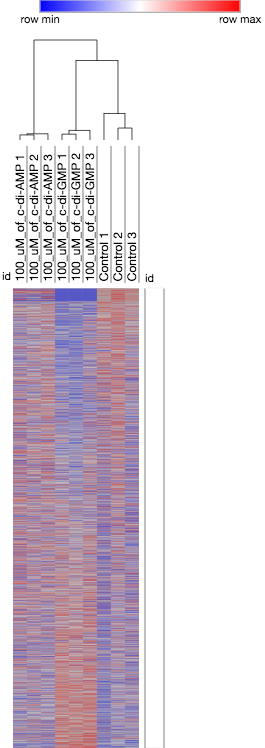


**Figure S1.** Heatmap showing hierarchical clustering of proteins found in the control and the c-di-AMP or c-di-GMP treatment. Data show distinct clustering into three groups. Maximum intensities are in red, while lower intensities are in blue. Hierarchical clustering and heatmaps were plotted with Morpheus, Version 2020 software (<https://software.broadinstitute.org/morpheus>).

**D.**


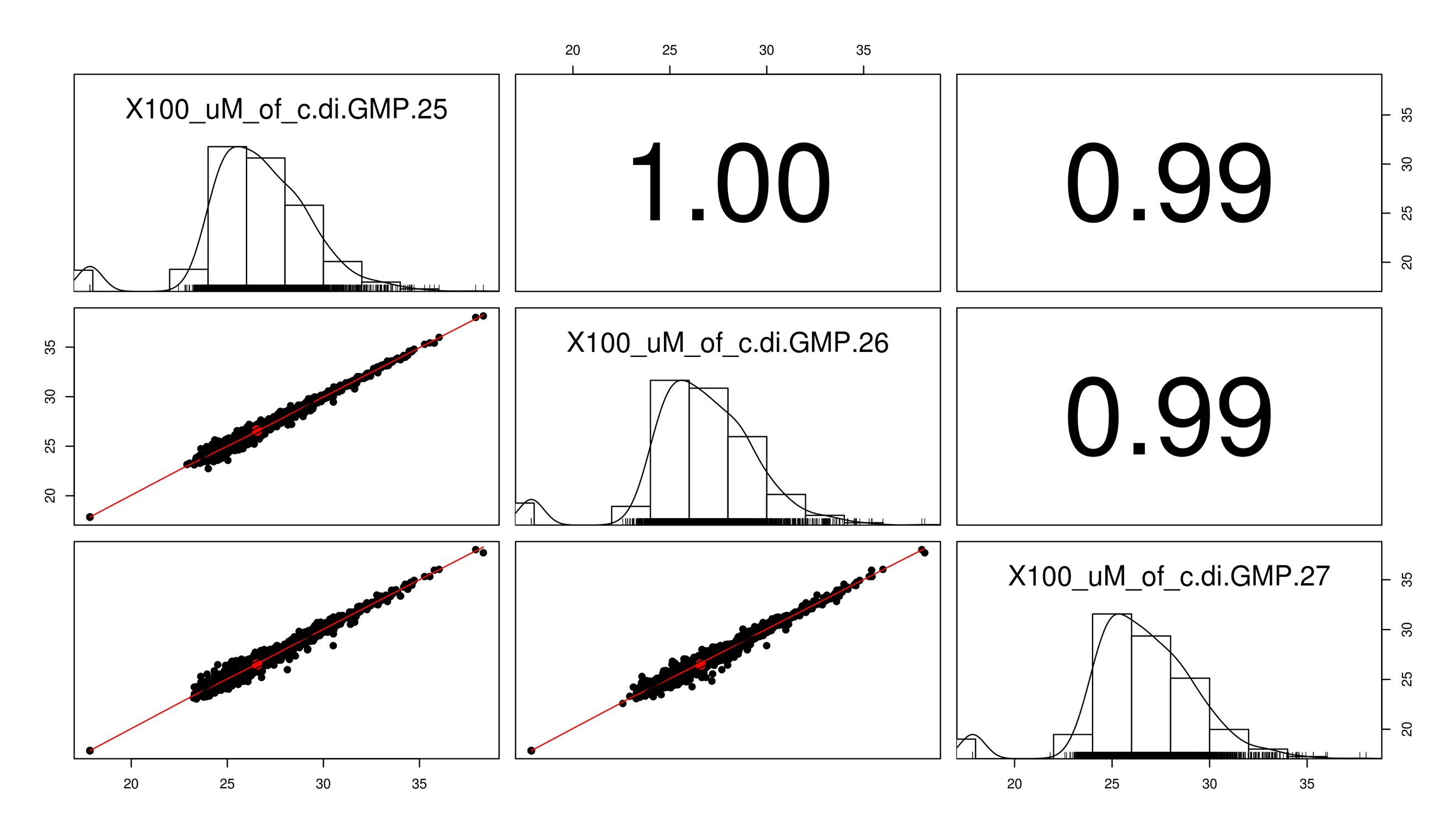

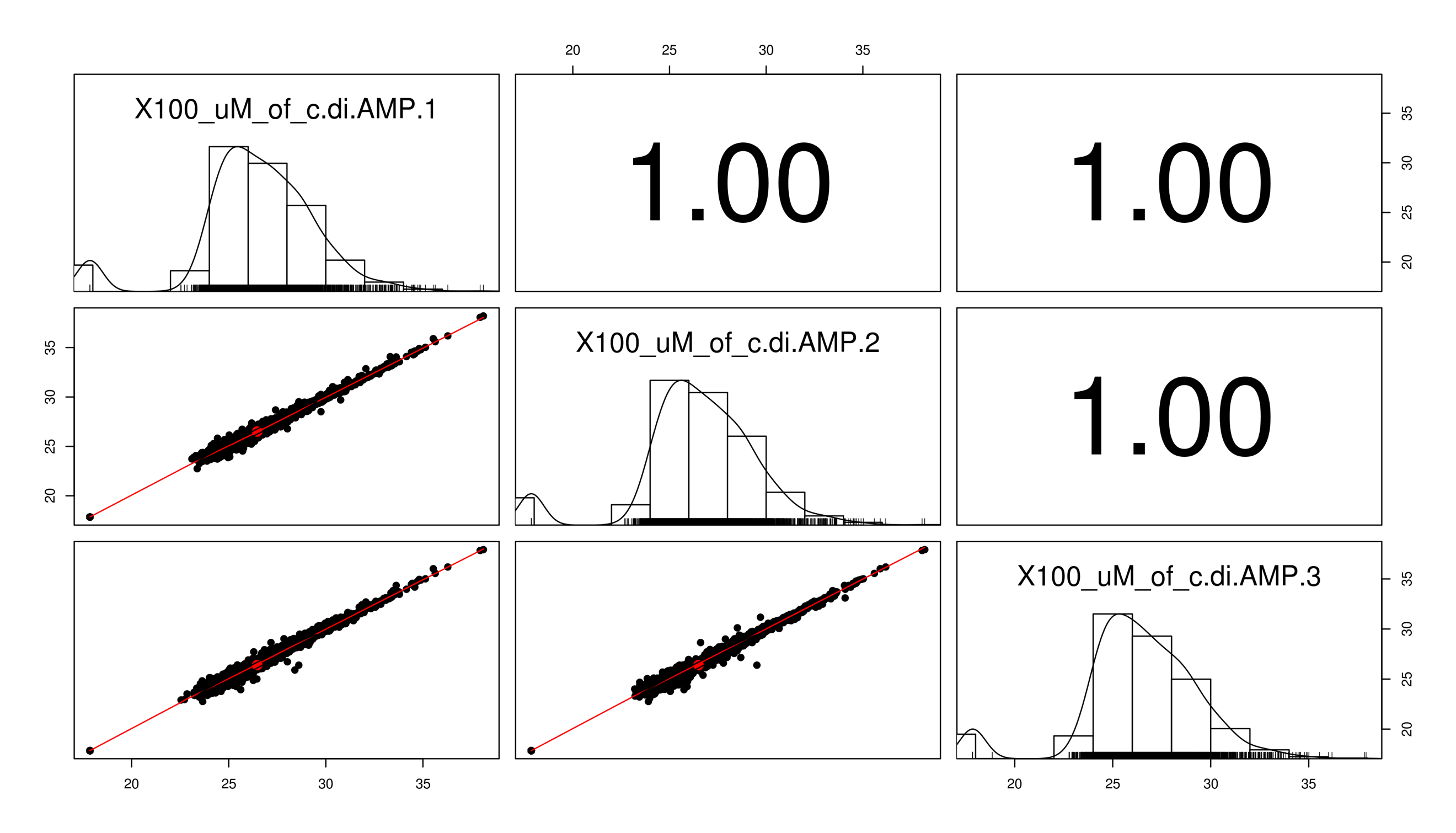

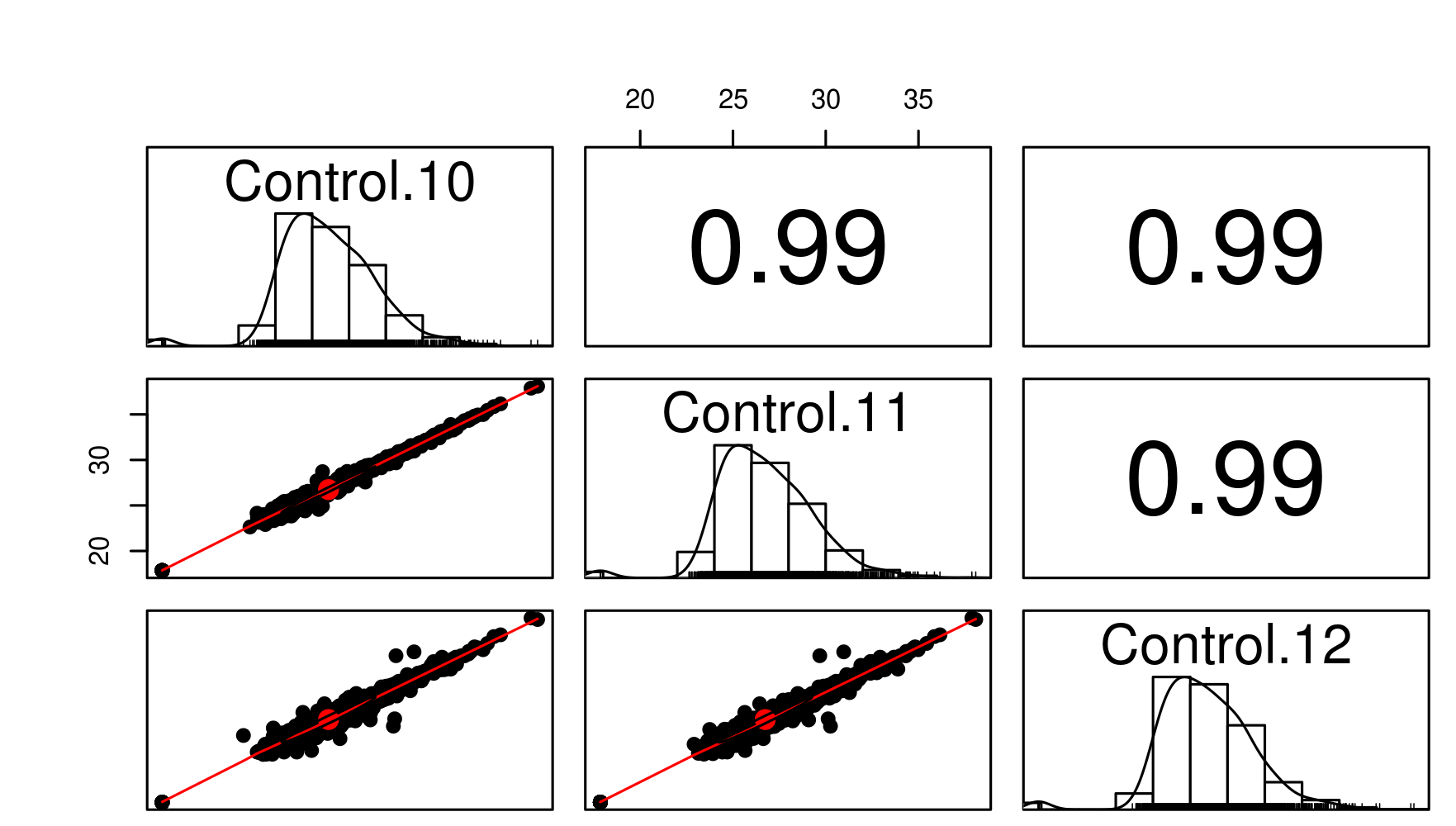


**C.**

**A.**

**B.**


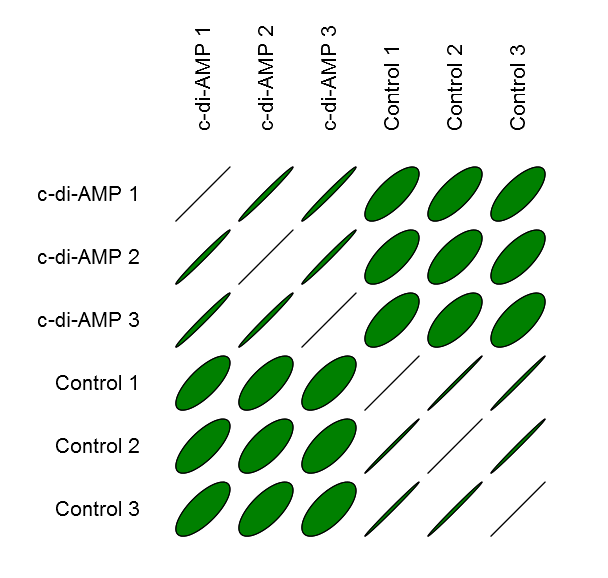

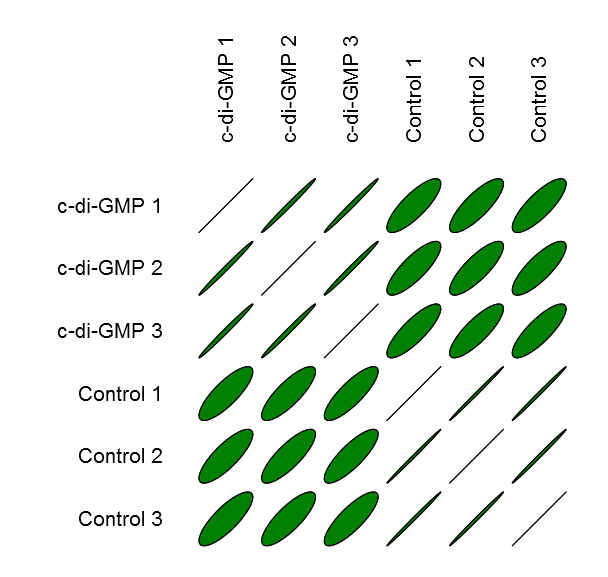


**E.**

**Figure S2.** Scatter plots showing good correlation amongst the biological replicates **(A-C)** and correlation plots showing good correlation between samples (**D** and **E)**

**Analysis of STING/TBK1/IRF3 pathway in human gingival fibroblasts**

To ascertain that HGFs have the capacity to respond to stimulation by c-di-AMP and c-di-GMP we used immunoblot assays to check for the presence of STING (42 kDa), TBK1 (84 kDa) and IRF3 (47 kDa) in HGFs. These proteins, which play the most critical roles in the CDN triggered STING/TBK1/IRF3 signaling pathway were found in HGFs at both 2 h and 24 h time points**.**


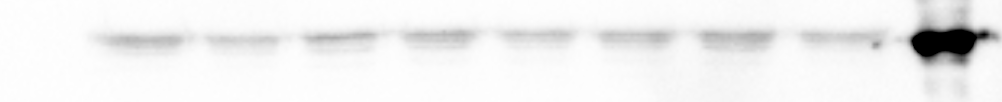

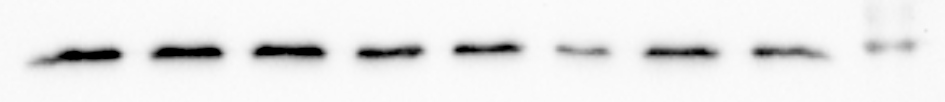

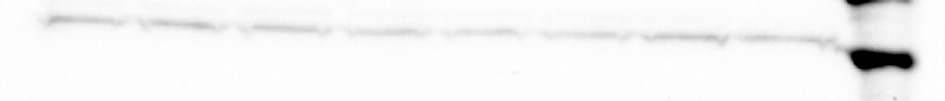


**STING 2h (42kDa)**

**TBK1 2h (84kDa)**

**IRF3 2h (47kDa)**

**ACTIN 2h (42kDa)**

**STING 24h (42kDa)**

**TBK1 24h (84kDa)**

**IRF3 24h (47kDa)**

**ACTIN 24h (42kDa)**


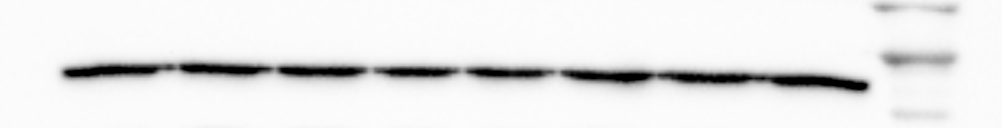

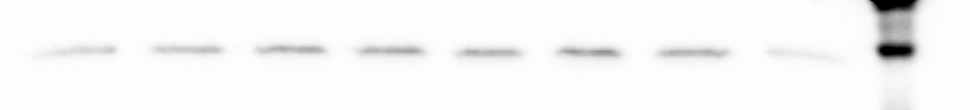

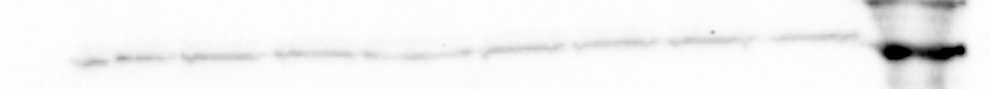

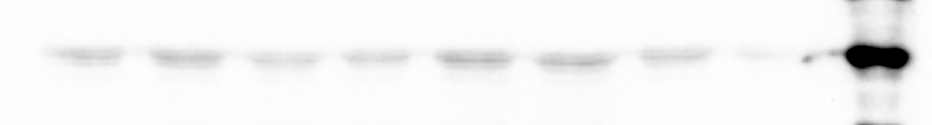

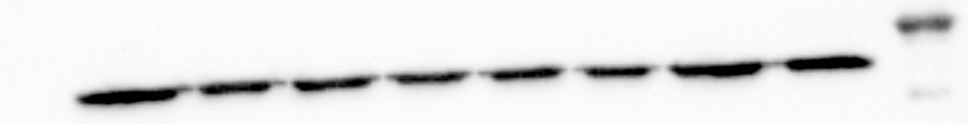


HGF Biological Replicates **Std**

**Figure S3.** Summary immunoblot assay to show STING/TBK1/IRF3 in human gingival fibroblasts. STING, TBK1, and IRF3 were found in all replicates. All original blots and standards are shown in **Figure S4** below.


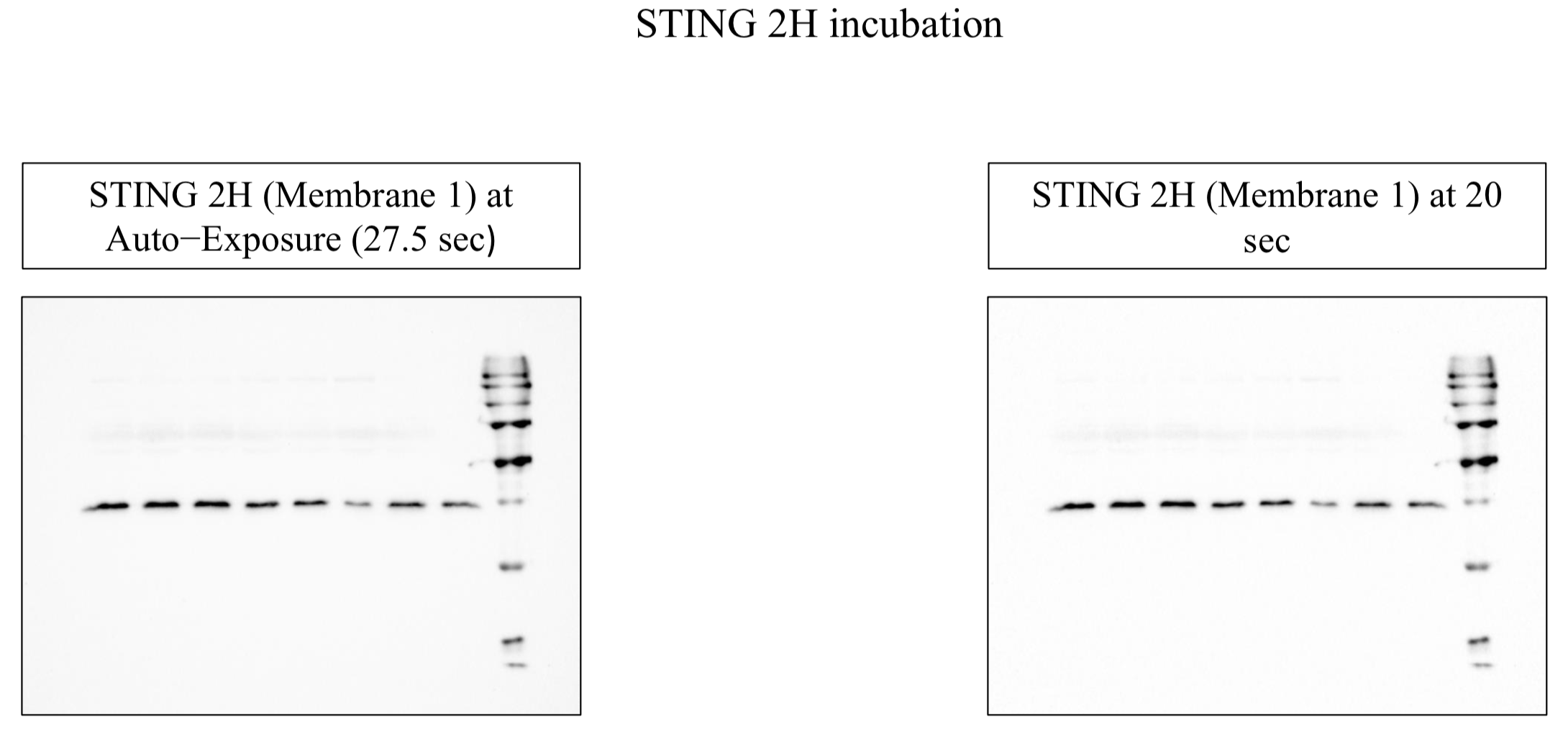


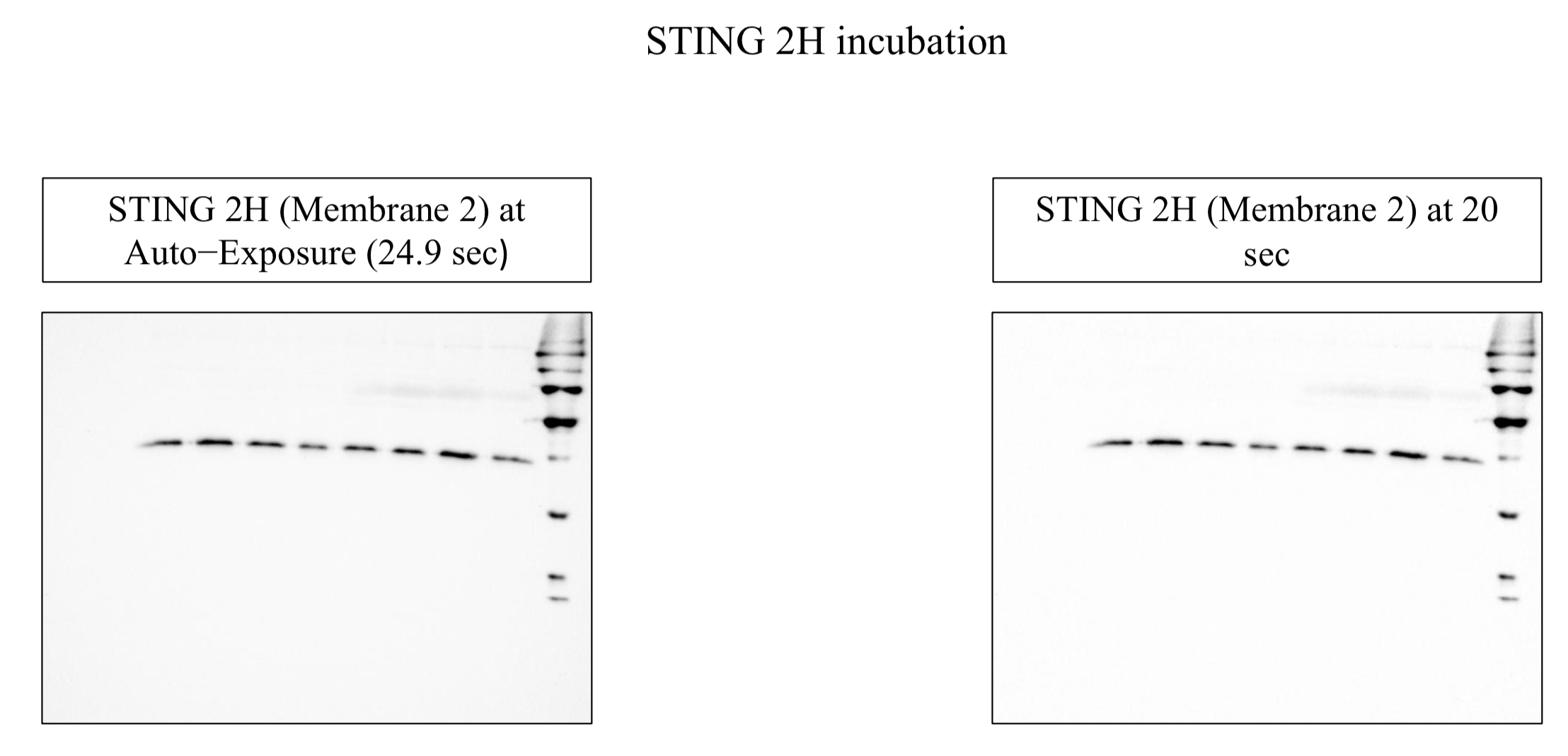


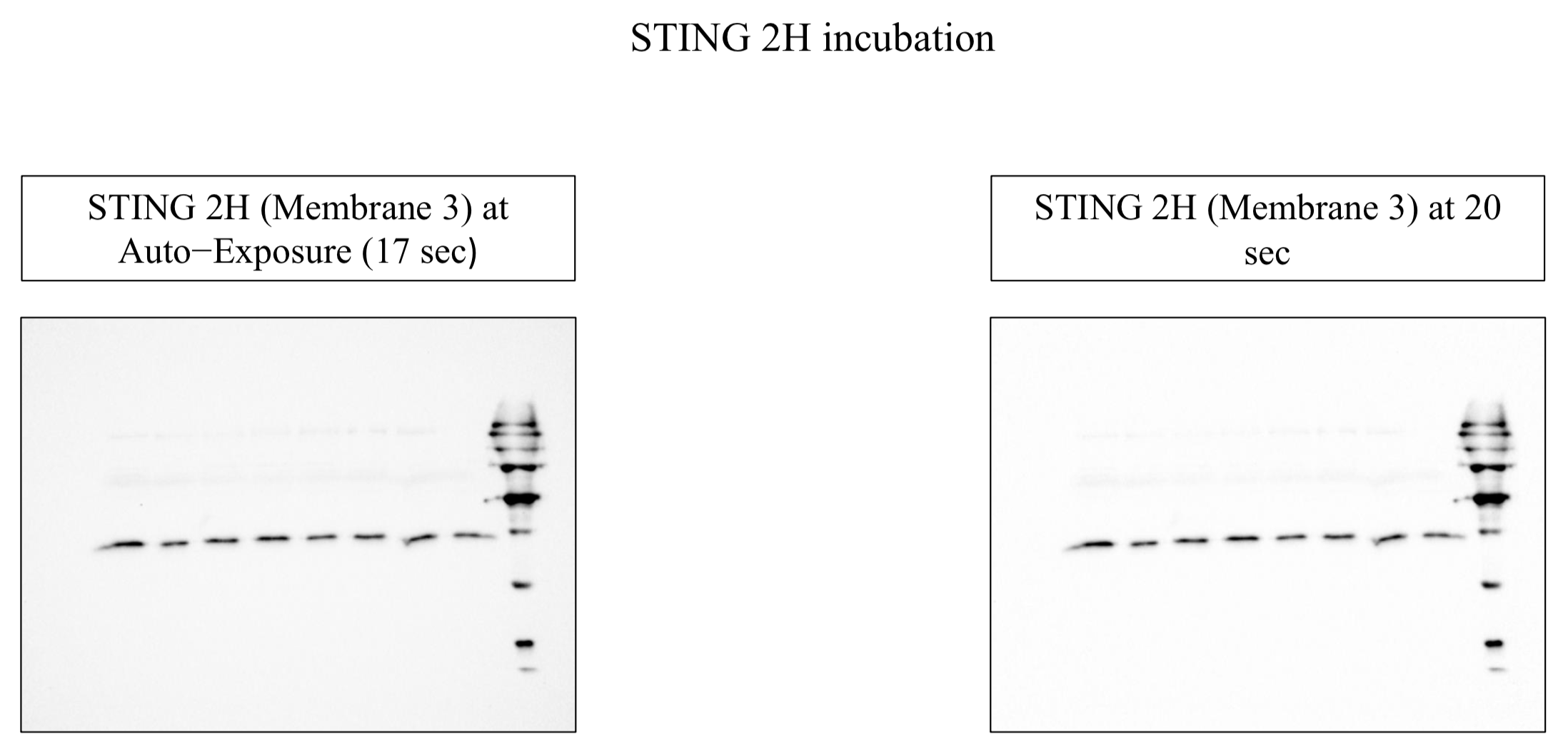


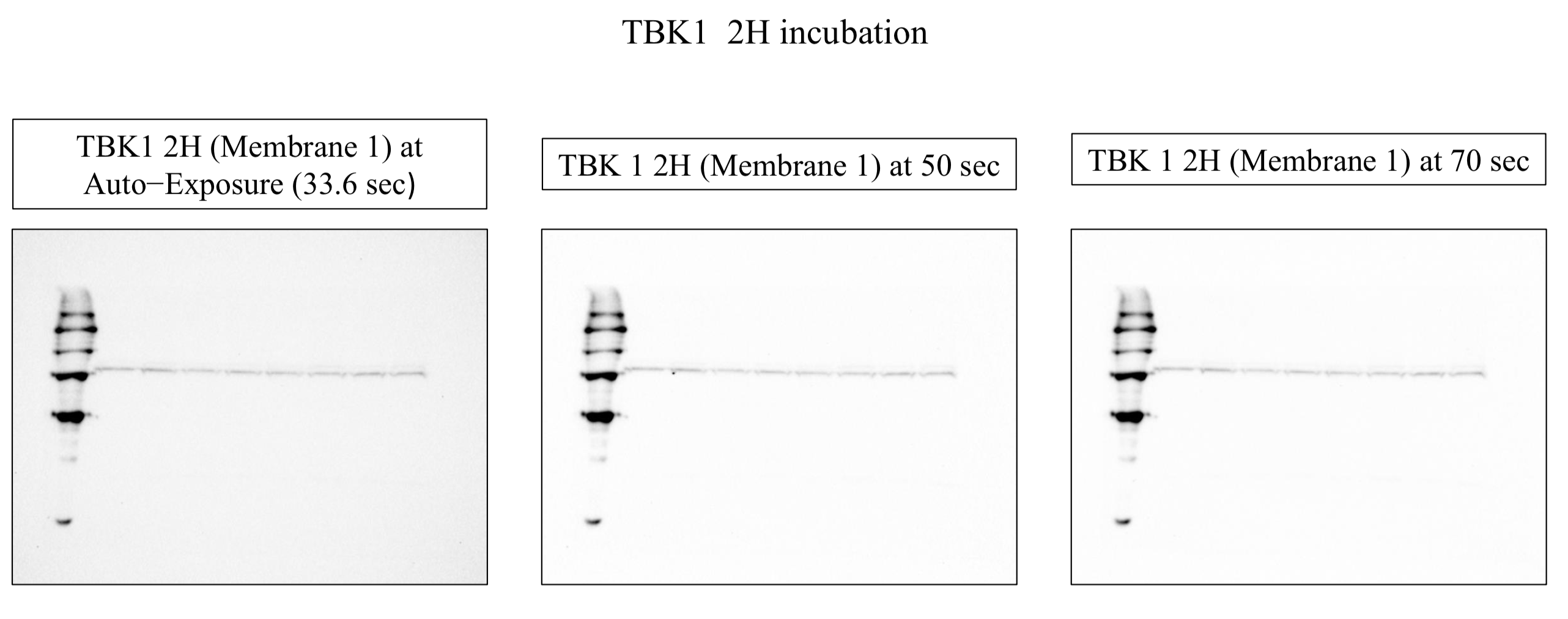


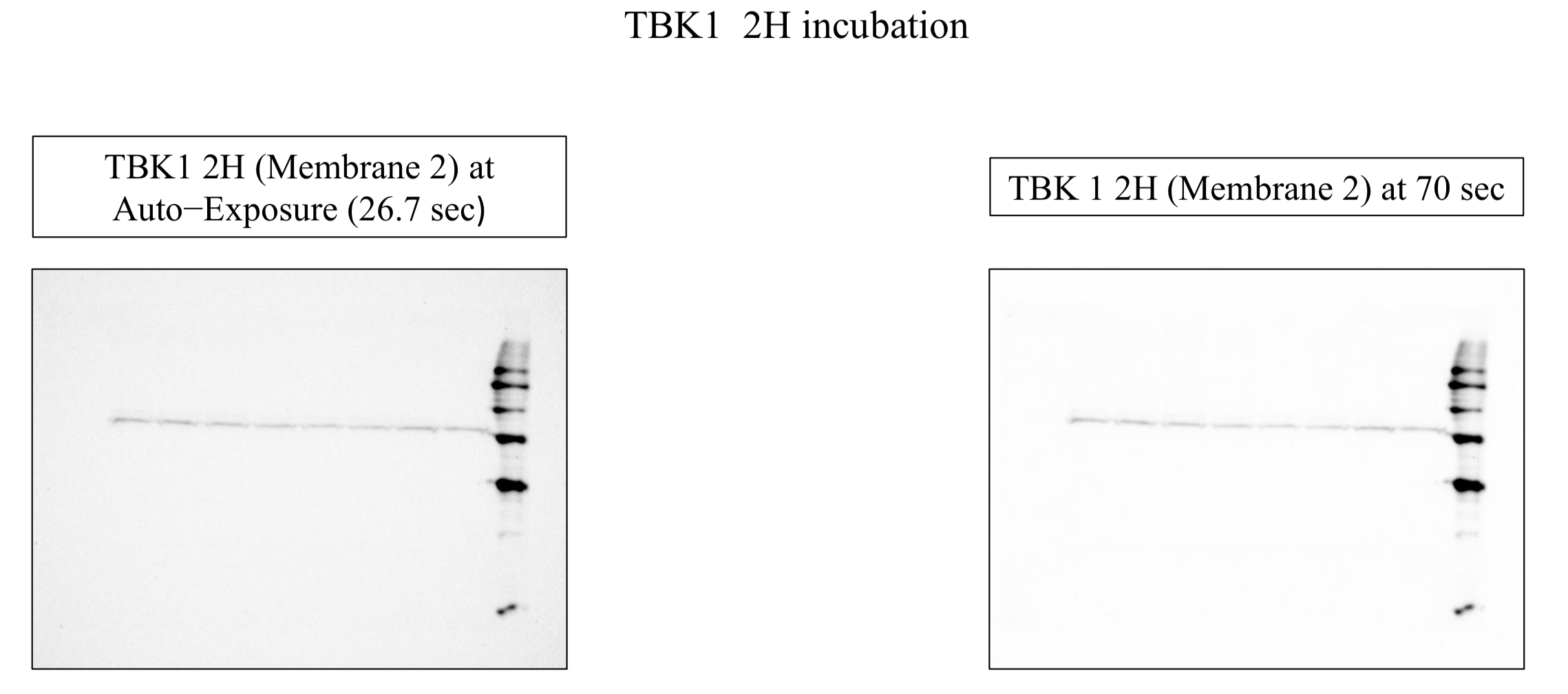


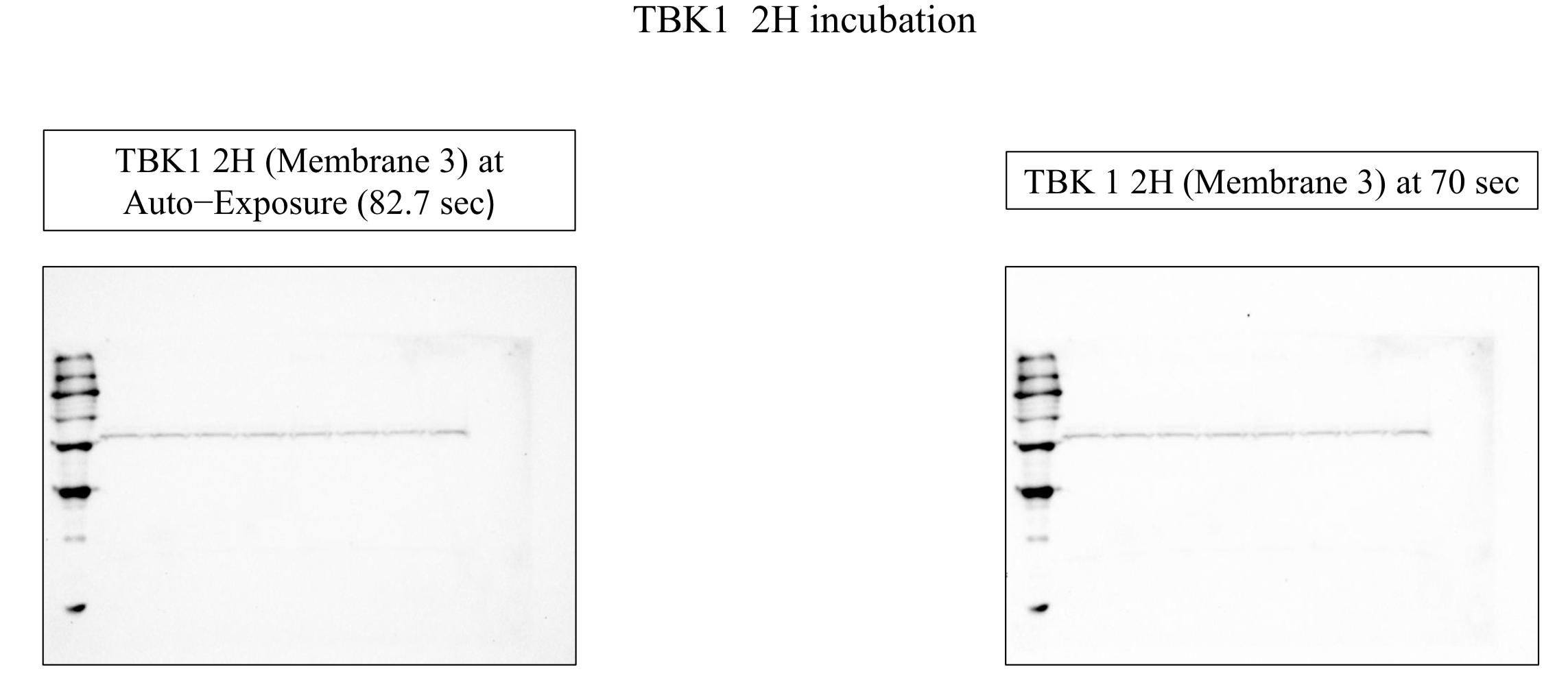


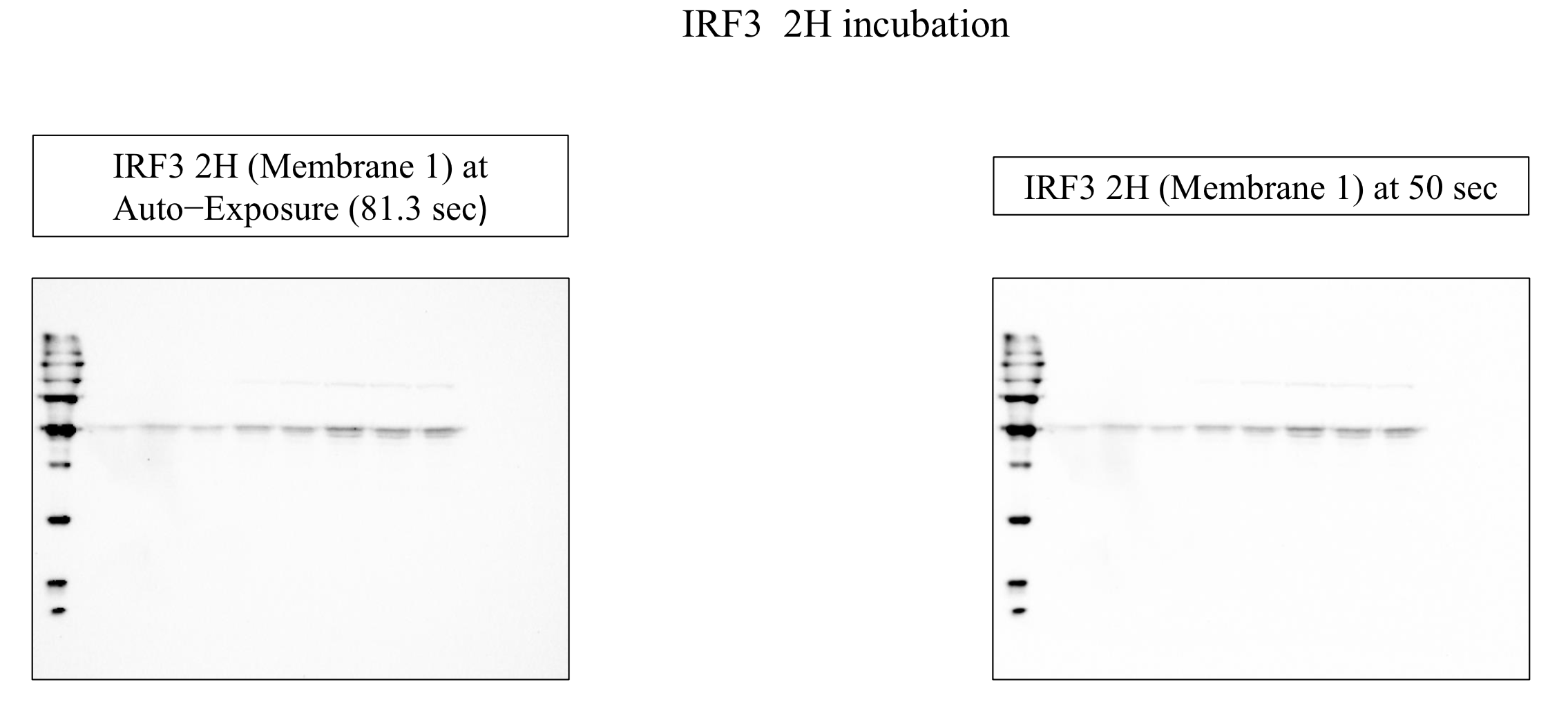


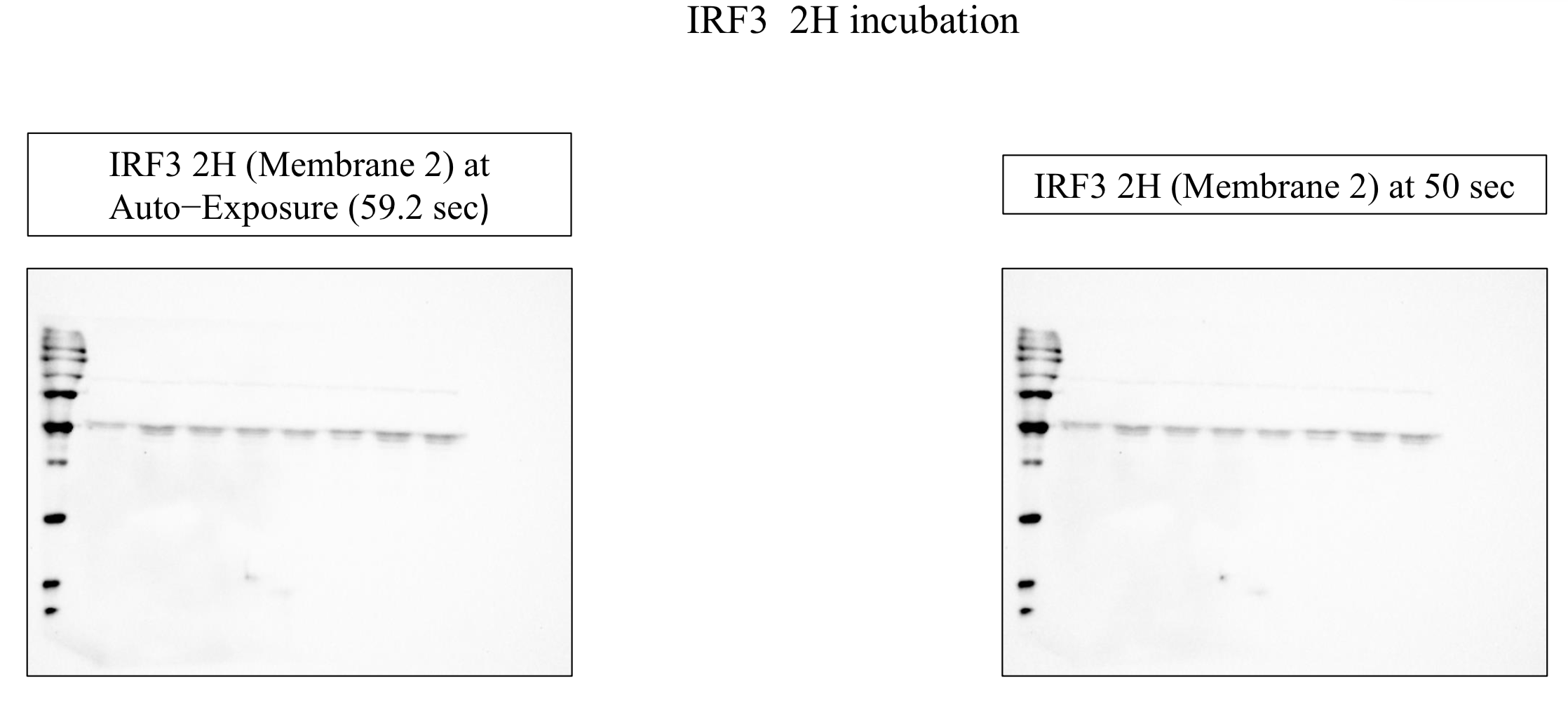


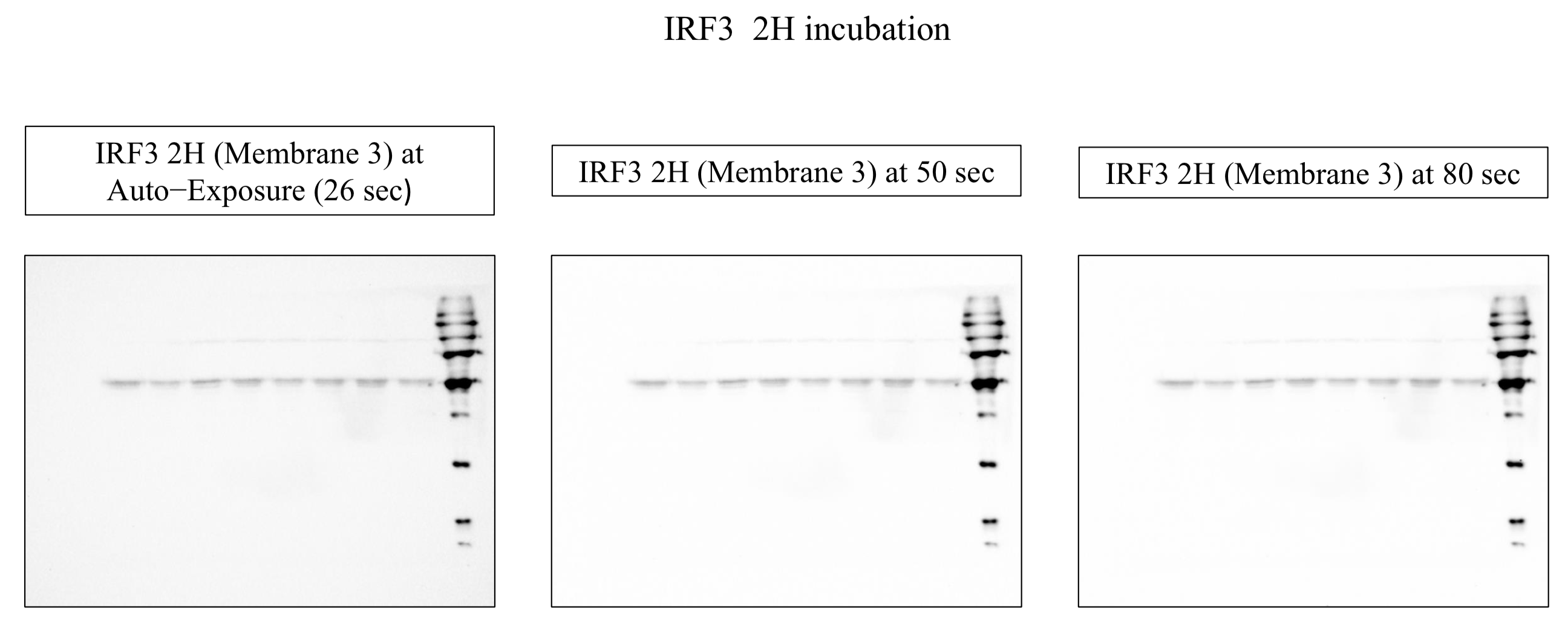


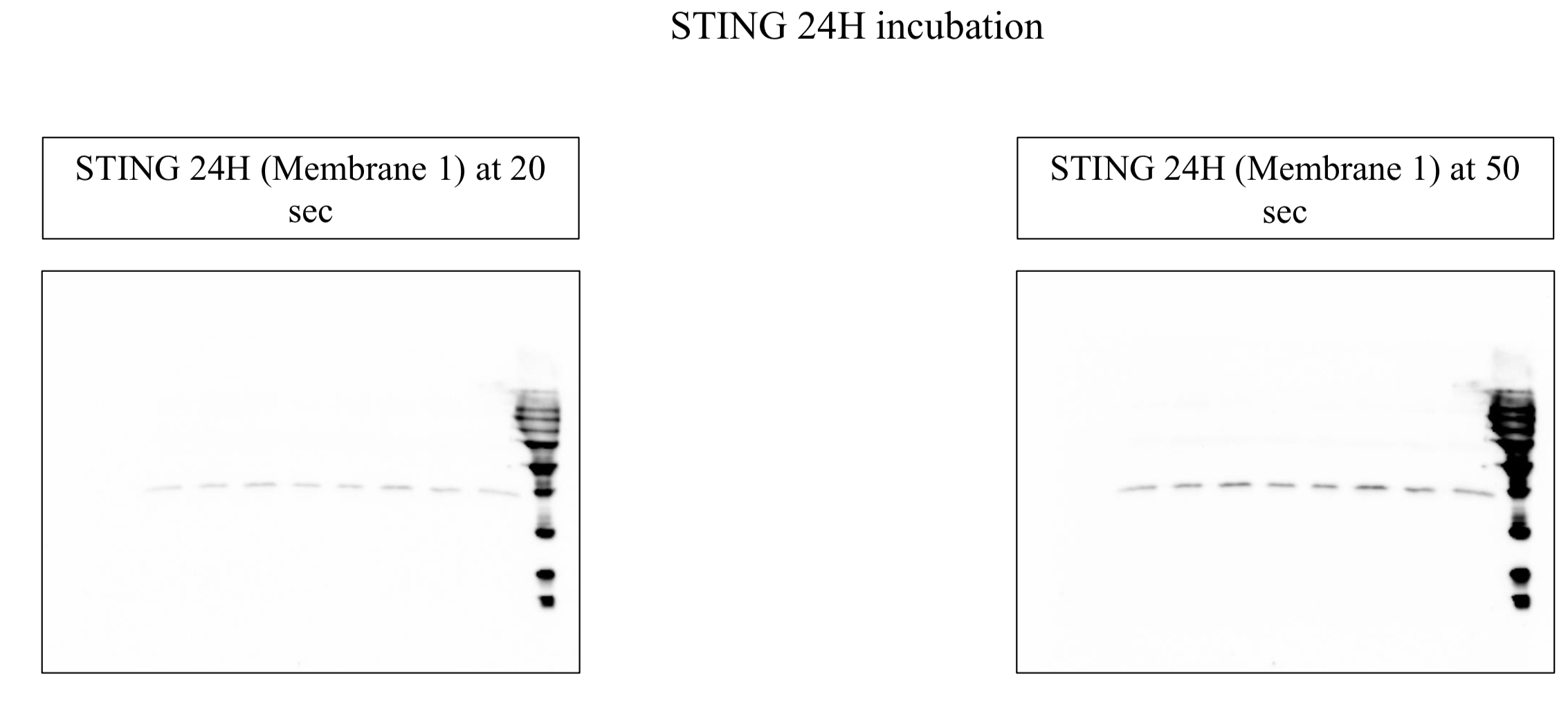


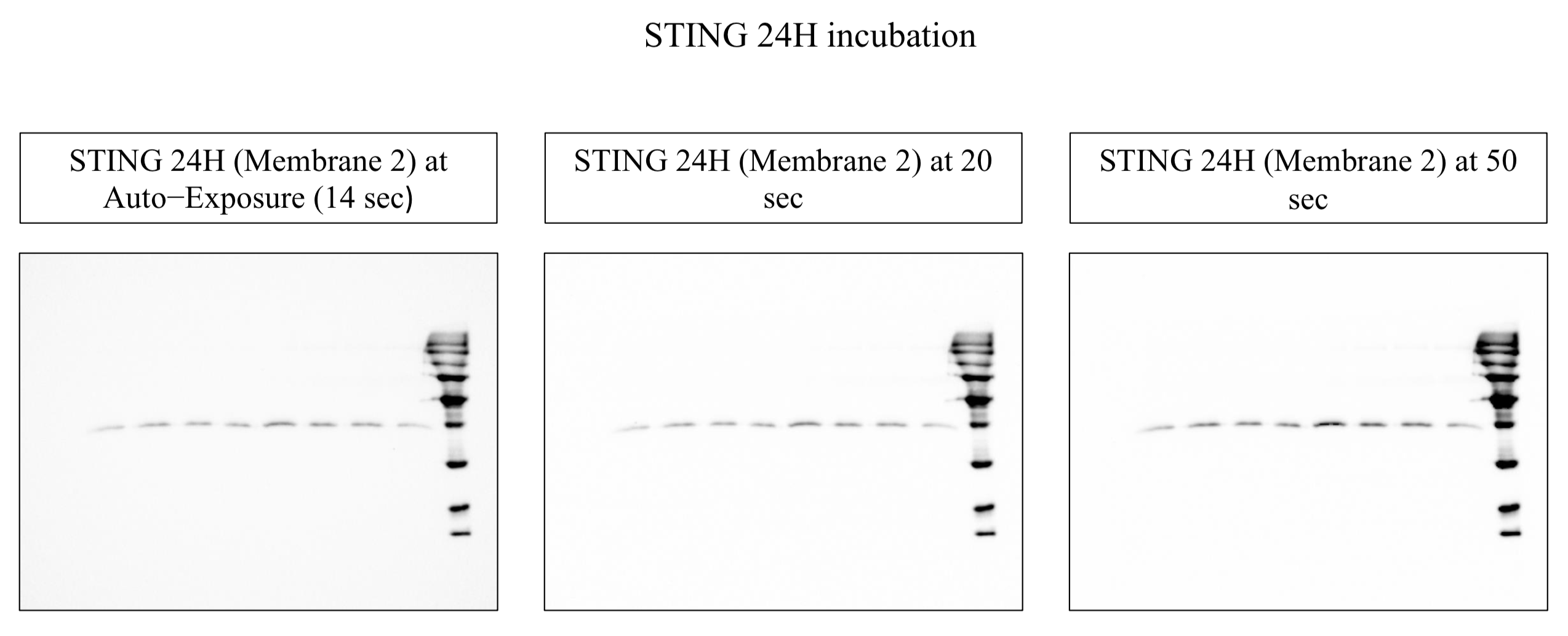


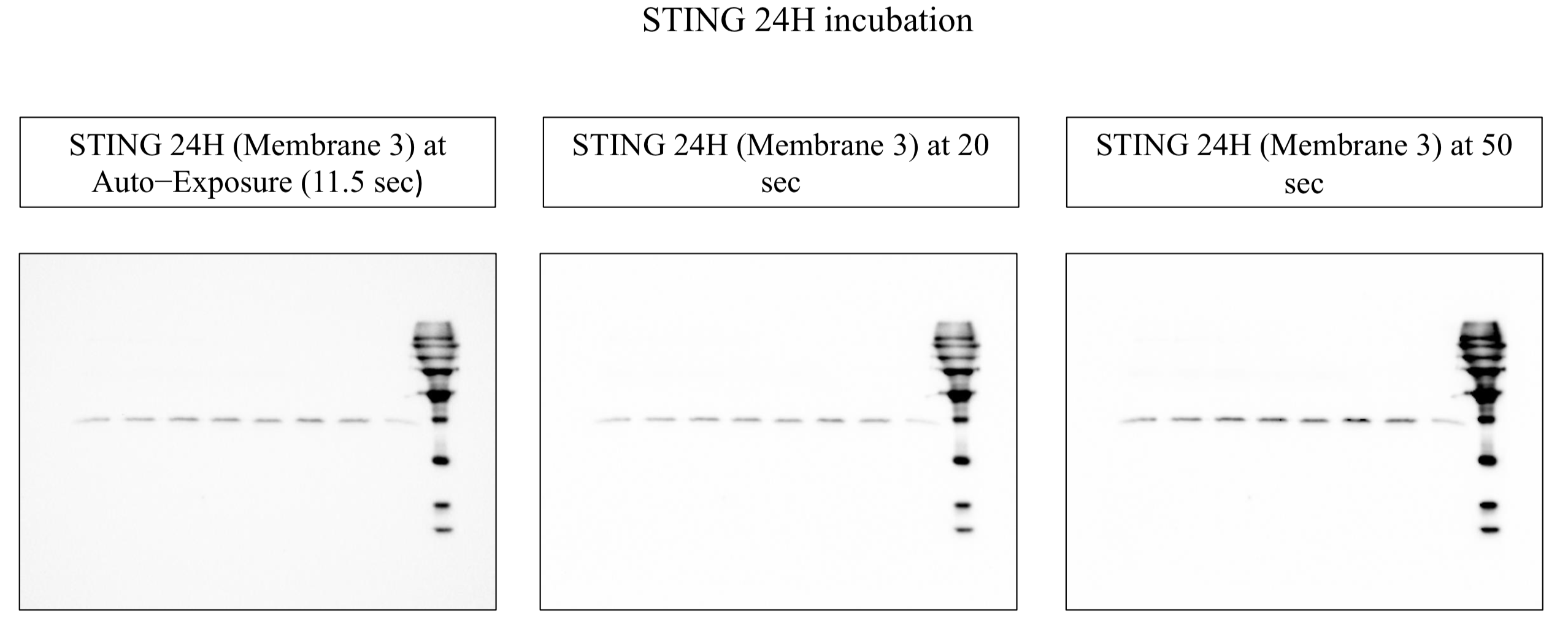


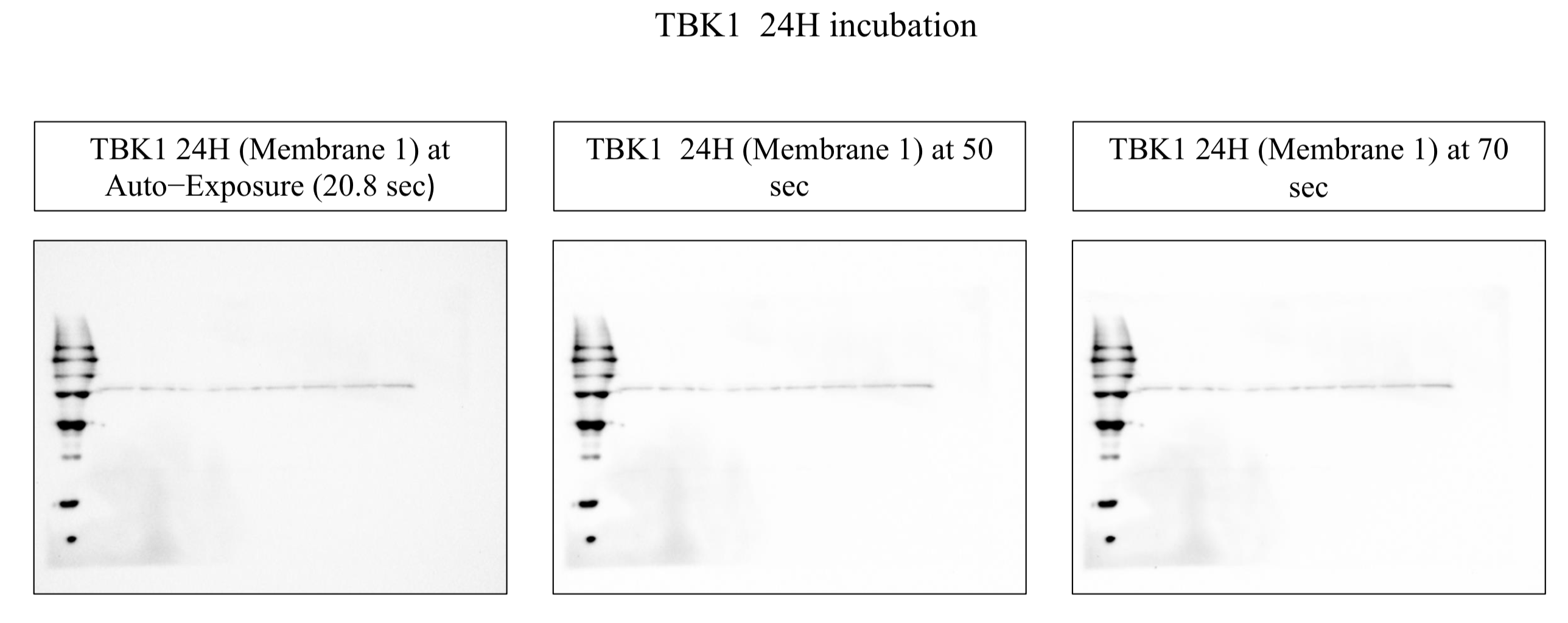


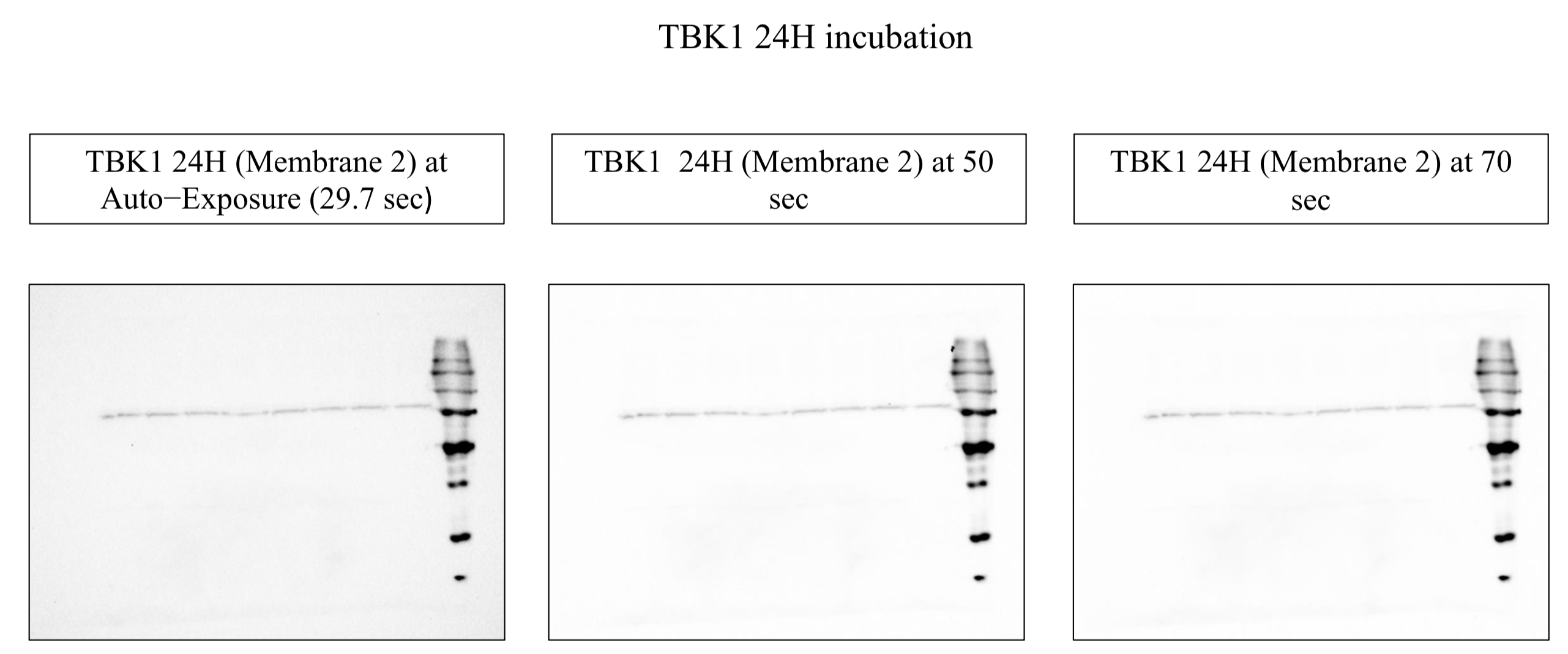


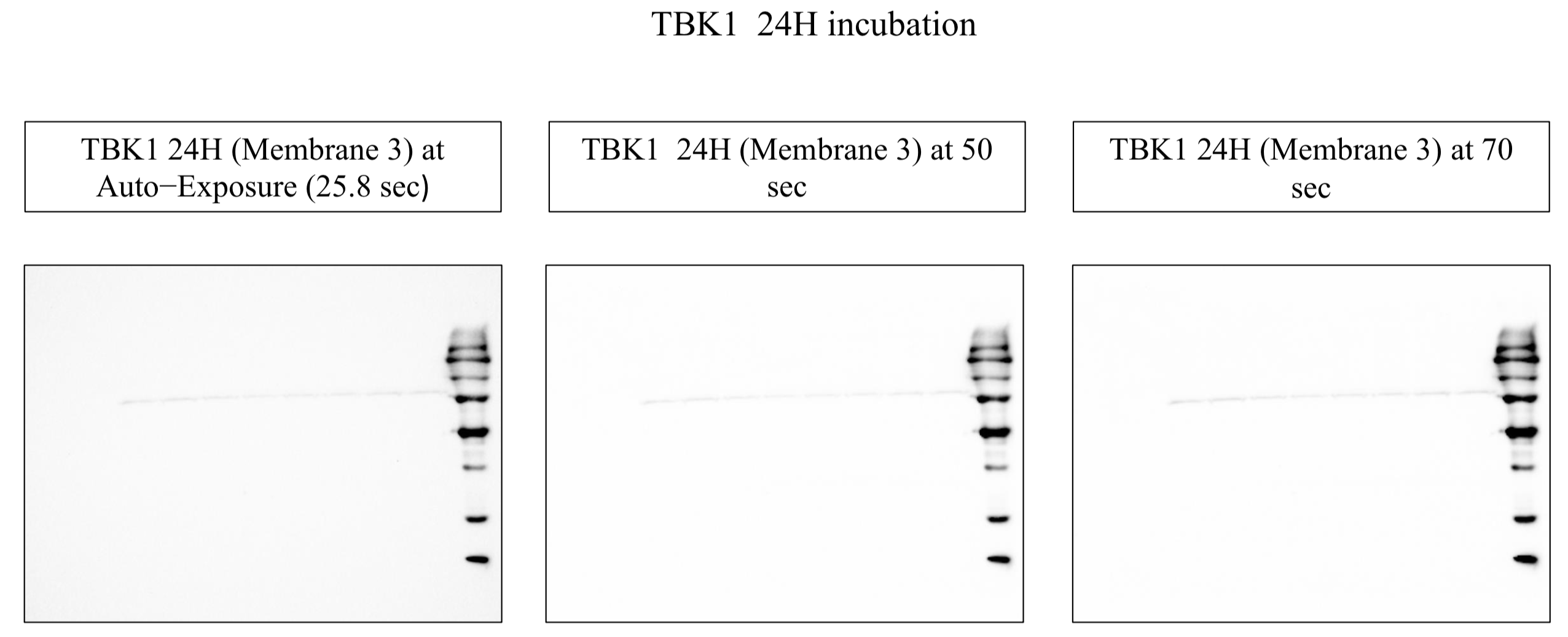


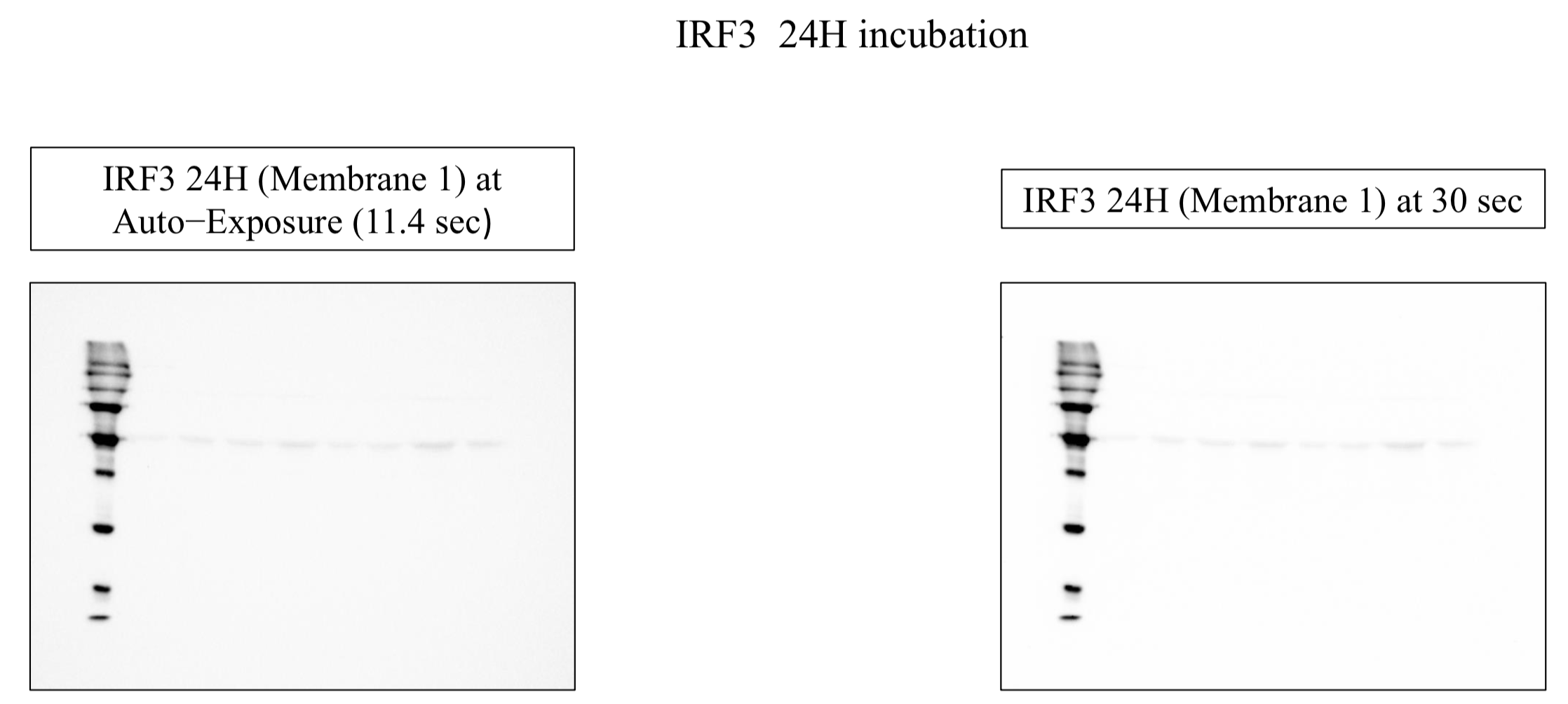


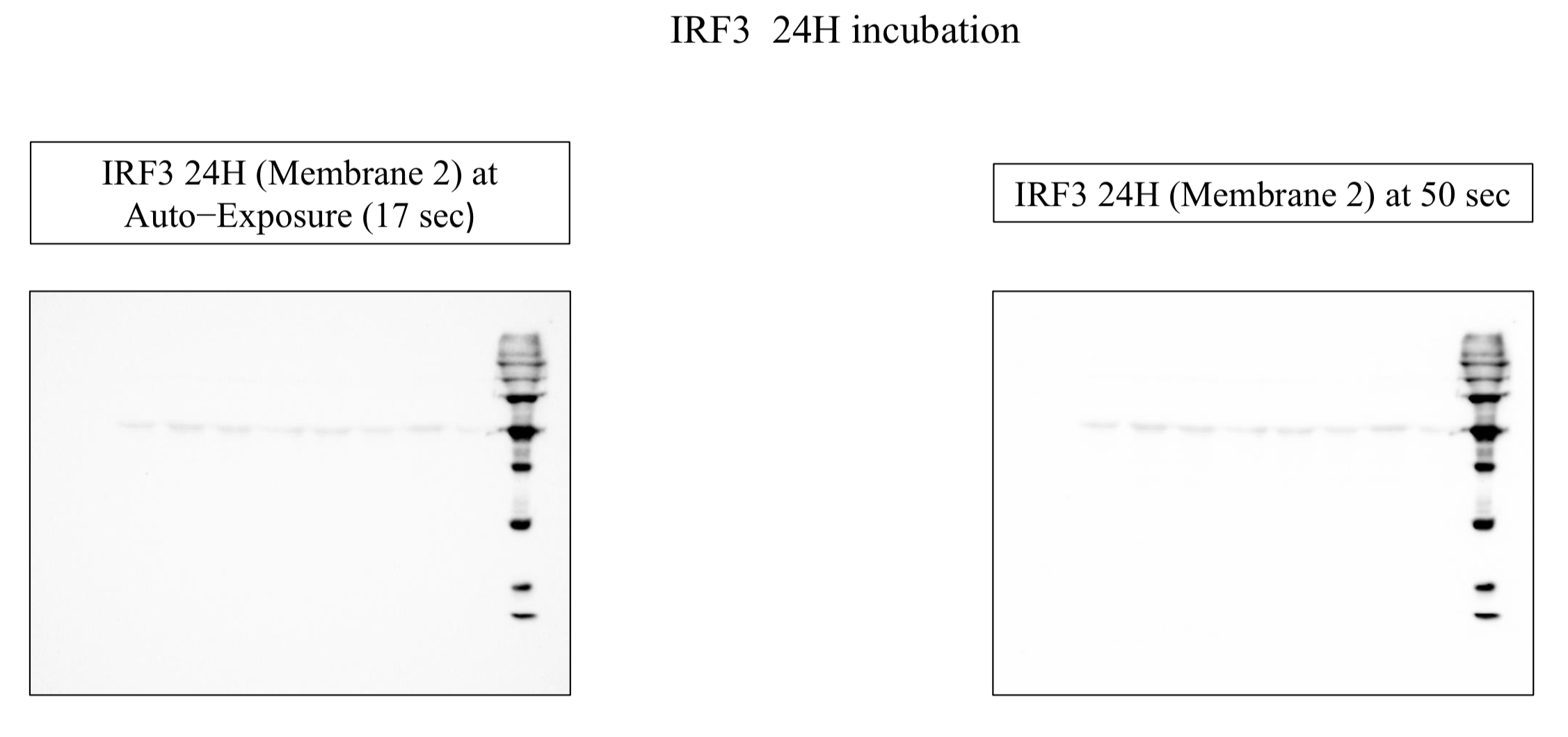


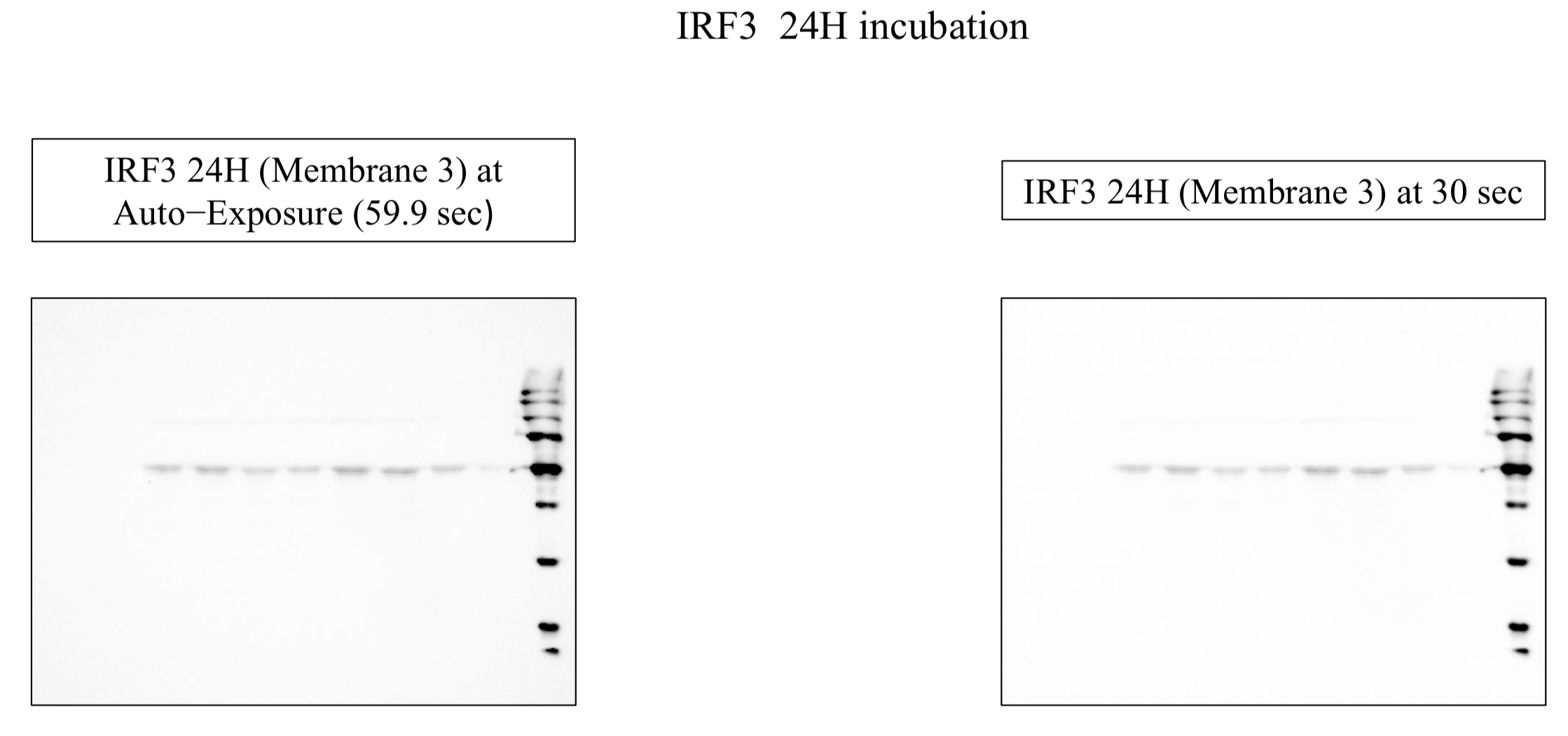


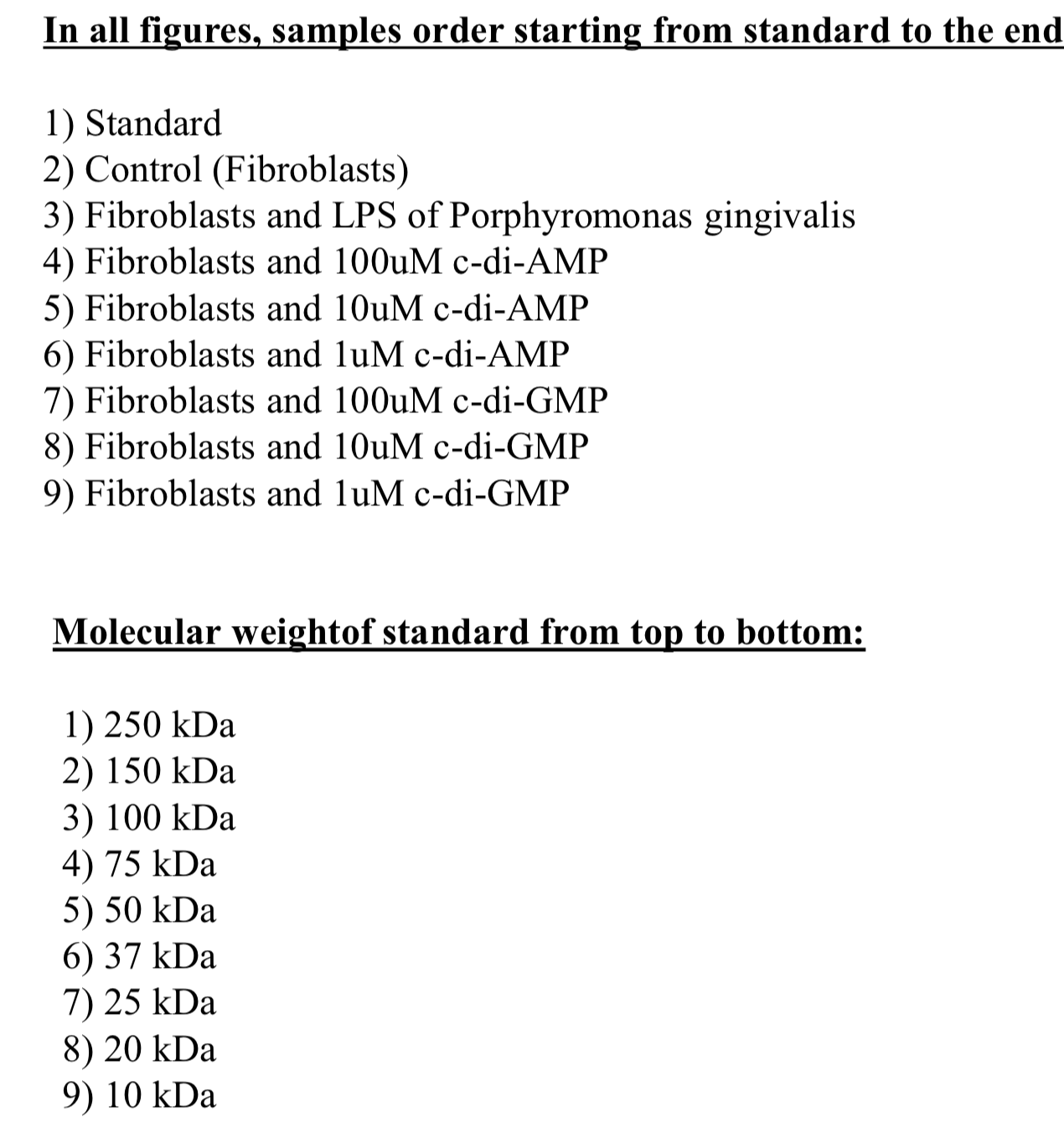


**Figure S4.** Original gel blots of STING/TBK1/IRF3 in human gingival fibroblasts at 2 h and 24 h. STING, TBK1, and IRF3 were found in all biological and technical replicates after treatment with c-di-AMP or c-di-GMP.


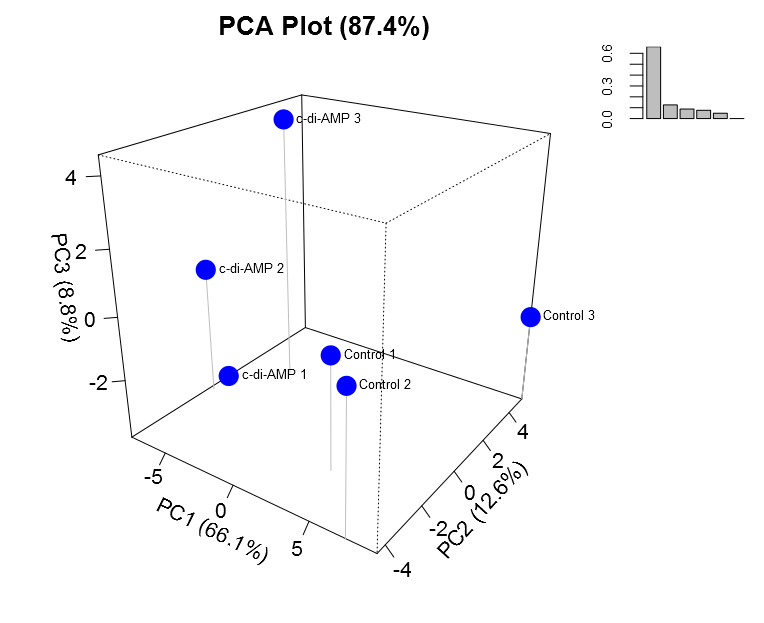

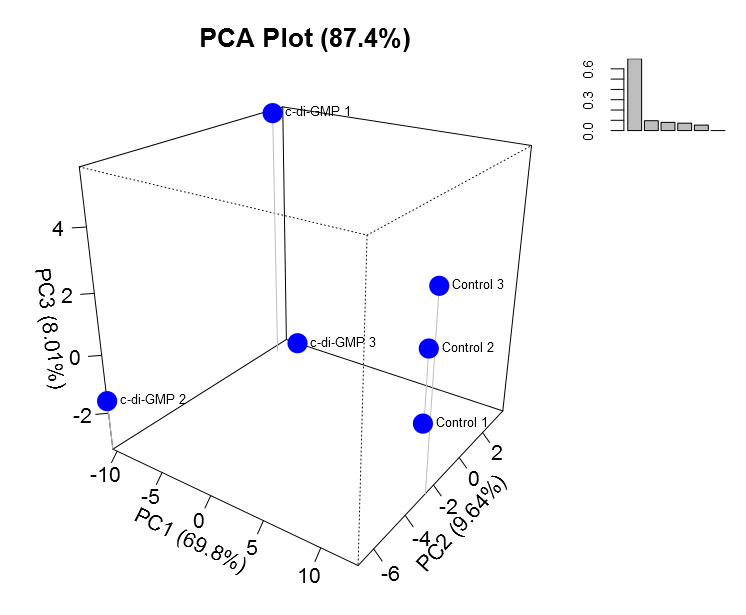


**A.**

**B.**

**Figure S3:** 3D Principal component analysis (PCA) plots showing mapping of differentially expressed proteins in the sample sets**. (A)** c-di-AMP; **(B)** c-di-GMP.

**Figure S5.** PCA plots showing the relative mapping of all the significant proteins in: (**A)** 100 µM c-di-AMP and; **(B)** 100 µM c-di-GMP treated human gingival fibroblasts


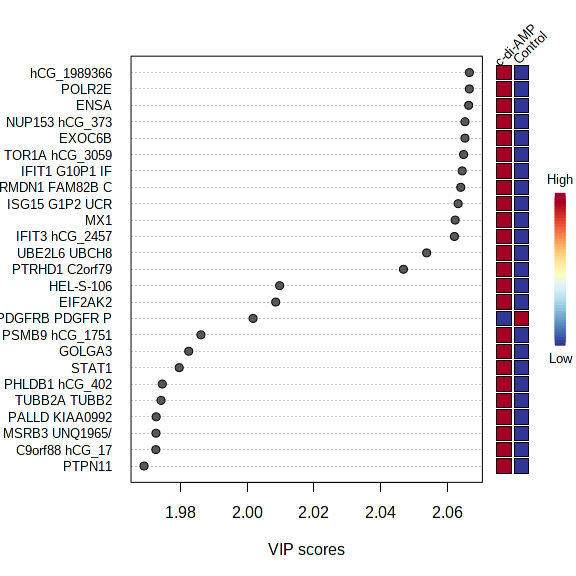

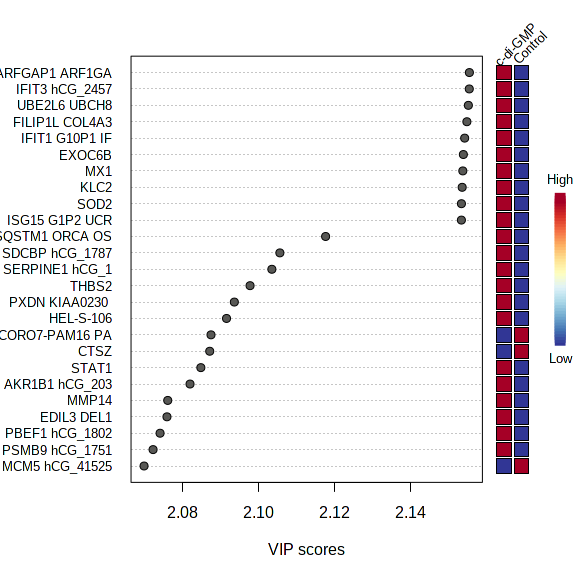


**A.**

**B.**

**Figure S6.** Important variables as identified by PLS-DA. The colored boxes on the right (red = upregulated, blue = downregulated) indicate the relative concentrations of the corresponding protein in each group. IFIT 1, IFIT3, ISG15, EXOC6B, UBE2L6, MX1, STAT1, HEL-S-106, PSMB9 hCG_1751 were similarly upregulated by both c-di-AMP and c-di-GMP with corresponding VIP scores. Four of these (IFIT 1, IFIT3, ISG15 and MX1) were also amongst the topmost statistically significant proteins upregulated in both c-di-AMP and c-di-GMP treated human gingival fibroblasts by t-test (p < 0.0001). See **Figures S7** and **S8.**


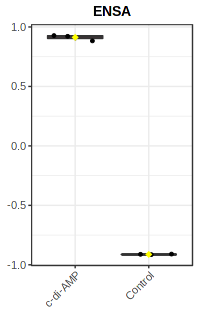

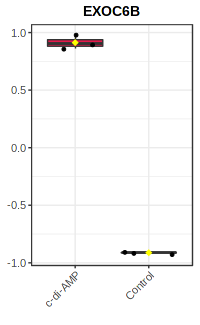

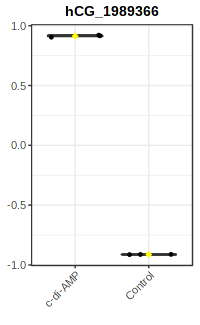

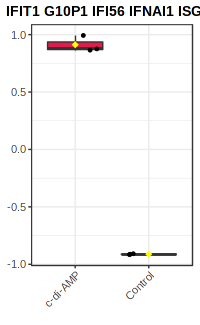

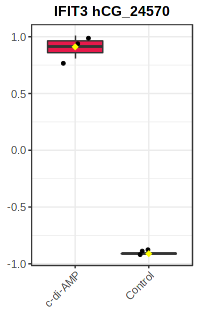

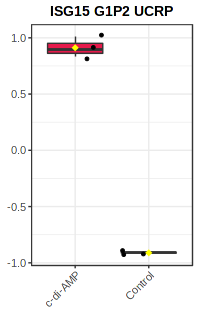

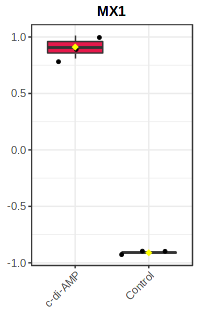

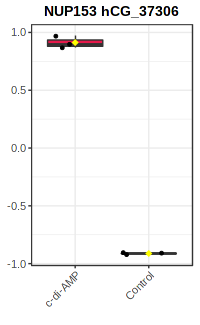

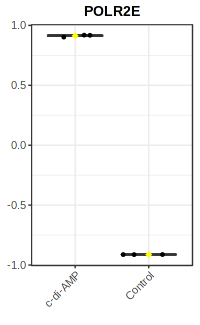

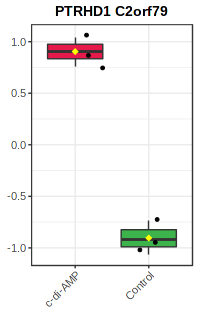

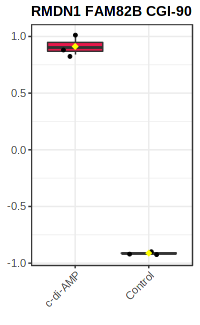

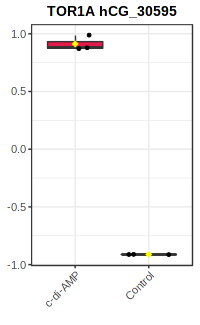


**A.**

**C.**


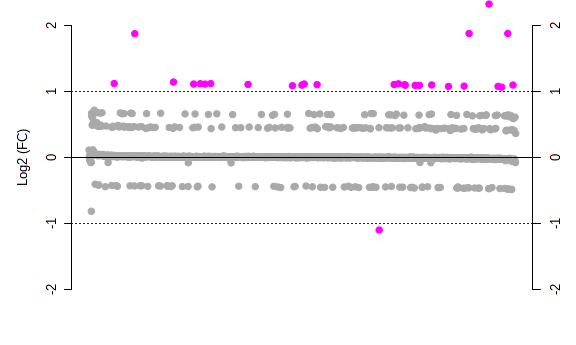

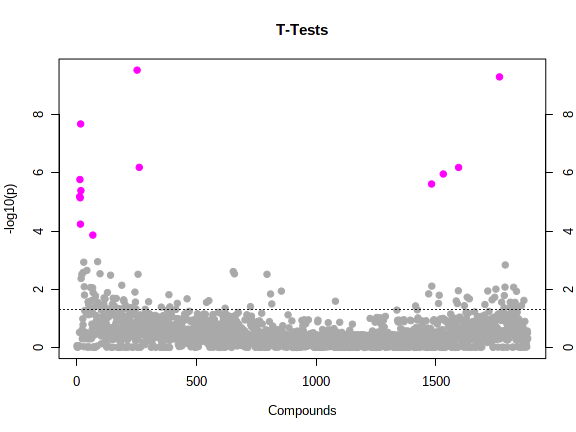


**B.**

**Figure S7.** 100 µM c-di-AMP treatment statistical analysis. **(A)** Fold change analysis with a Log2 fold change threshold of 1 (2 fold increase) to pick out the most affected proteins (magenta). **(B)** T test with p values transformed by –Log10 so that most significant proteins (with smaller p values) are seen higher up on the graph (12 magenta dots). **(C)** Graphs of the most significant proteins (the 12 magenta dots from **(B)**)


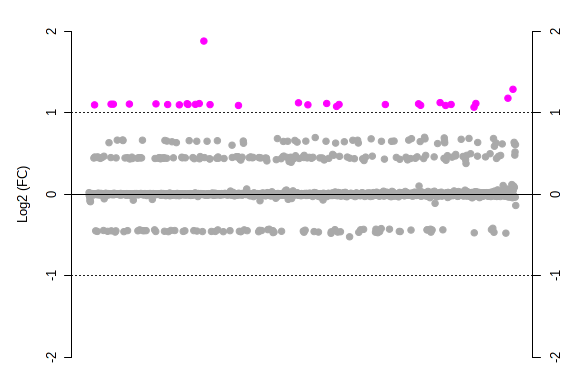

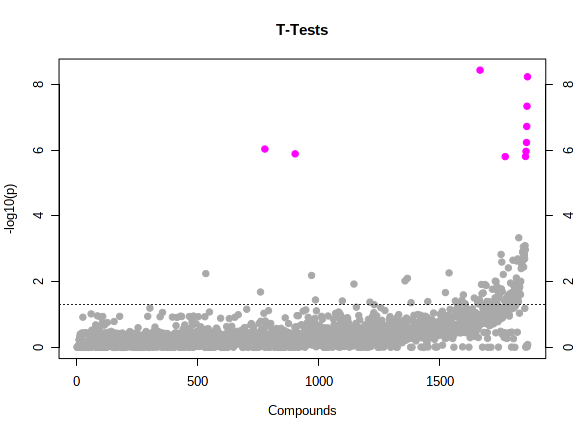


**A.**

**B.**


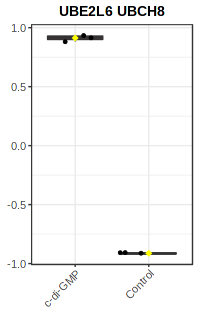

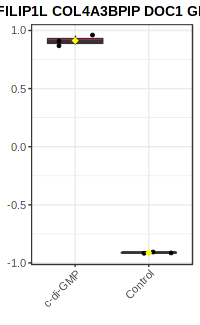

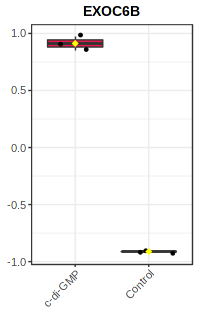

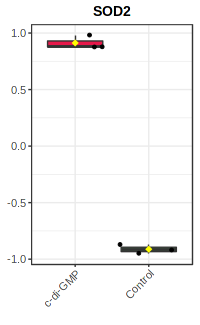

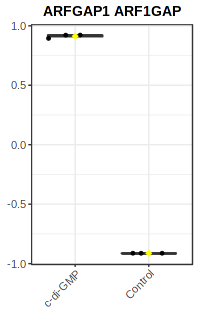

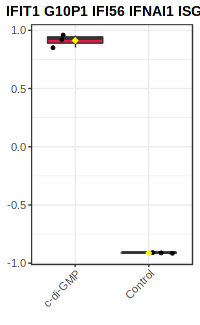

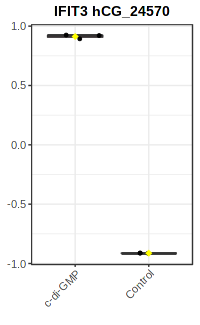

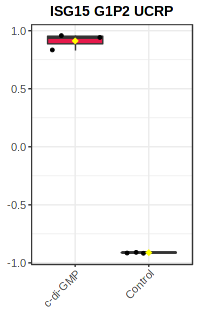

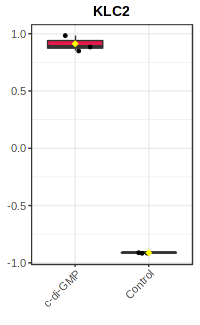

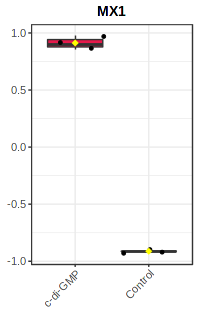


**C.**

**Figure S8.** 100 µM c-di-GMP treatment statistical analysis. **(A)** Fold change analysis with a Log2 fold change threshold of 1 (2 fold increase) to pick out the most affected proteins (magenta). **(B)** T test with p values transformed by –log10 so that most significant proteins (with smaller p values) are seen higher up on the graph (10 magenta dots). **(C)** Graphs of the most significant proteins (the 10 magenta dots from **(B)**)


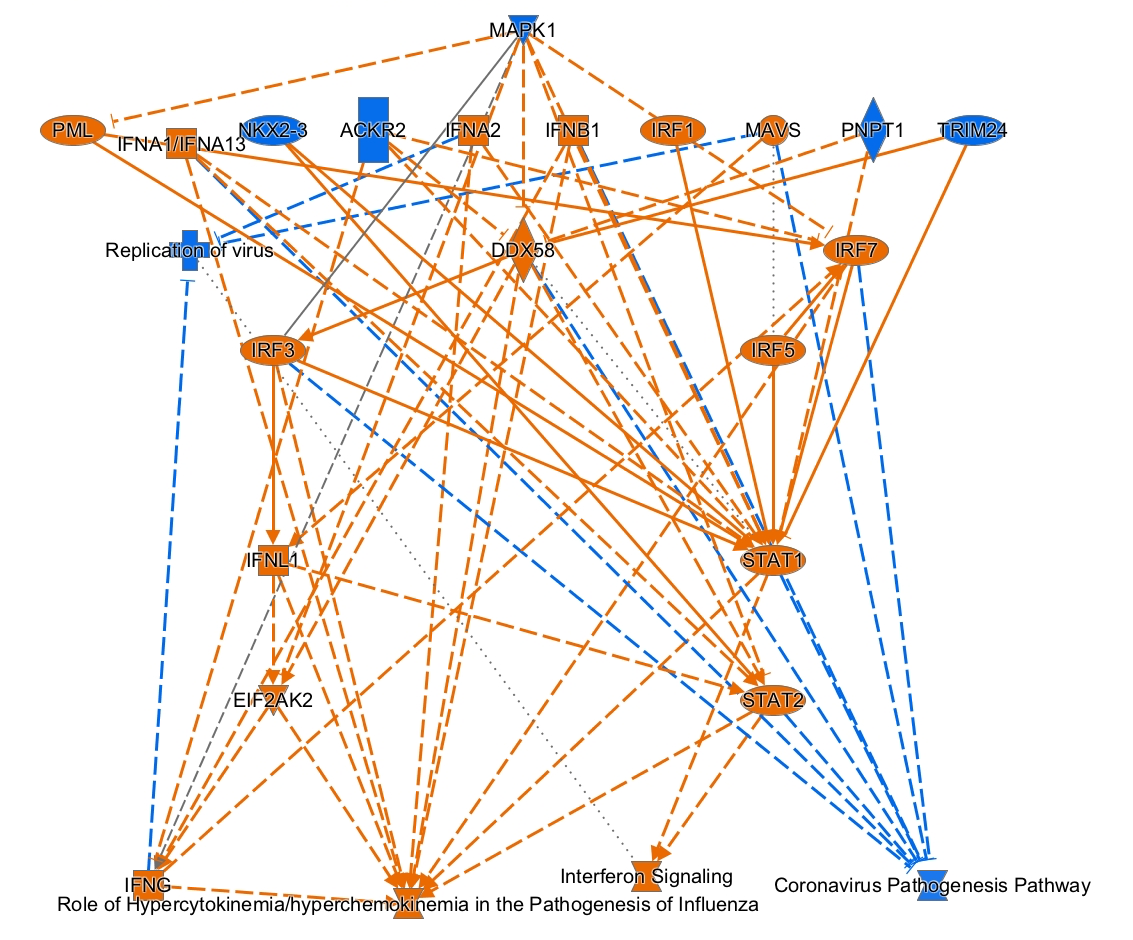


**Graphical summary of c-di-AMP Pathways**

**A.**

**B.**

**Graphical summary of c-di-GMP Pathways**


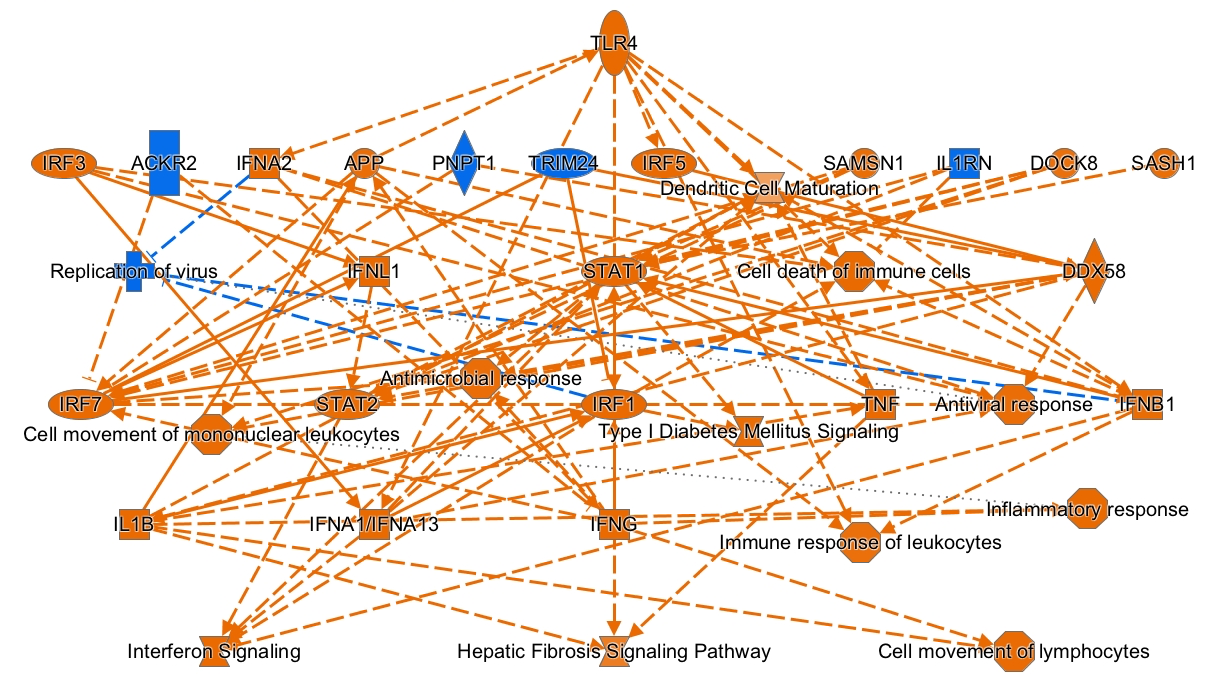


**Figure S9.** Graphical summary of enrichment analysis of ingenuity pathways regulated by (A) c-di-AMP and (B) c-di-GMP. The functional analyses and enrichment were generated using IPA (QIAGEN Inc., https://www.qiagenbioinformatics.com/products/ingenuity-pathway-analysis). Dotted lines indicate indirect relationships while solid lines indicate direct relationships. Orange lines or shapes signify activation whilst blue lines or shapes denote inhibition.

**
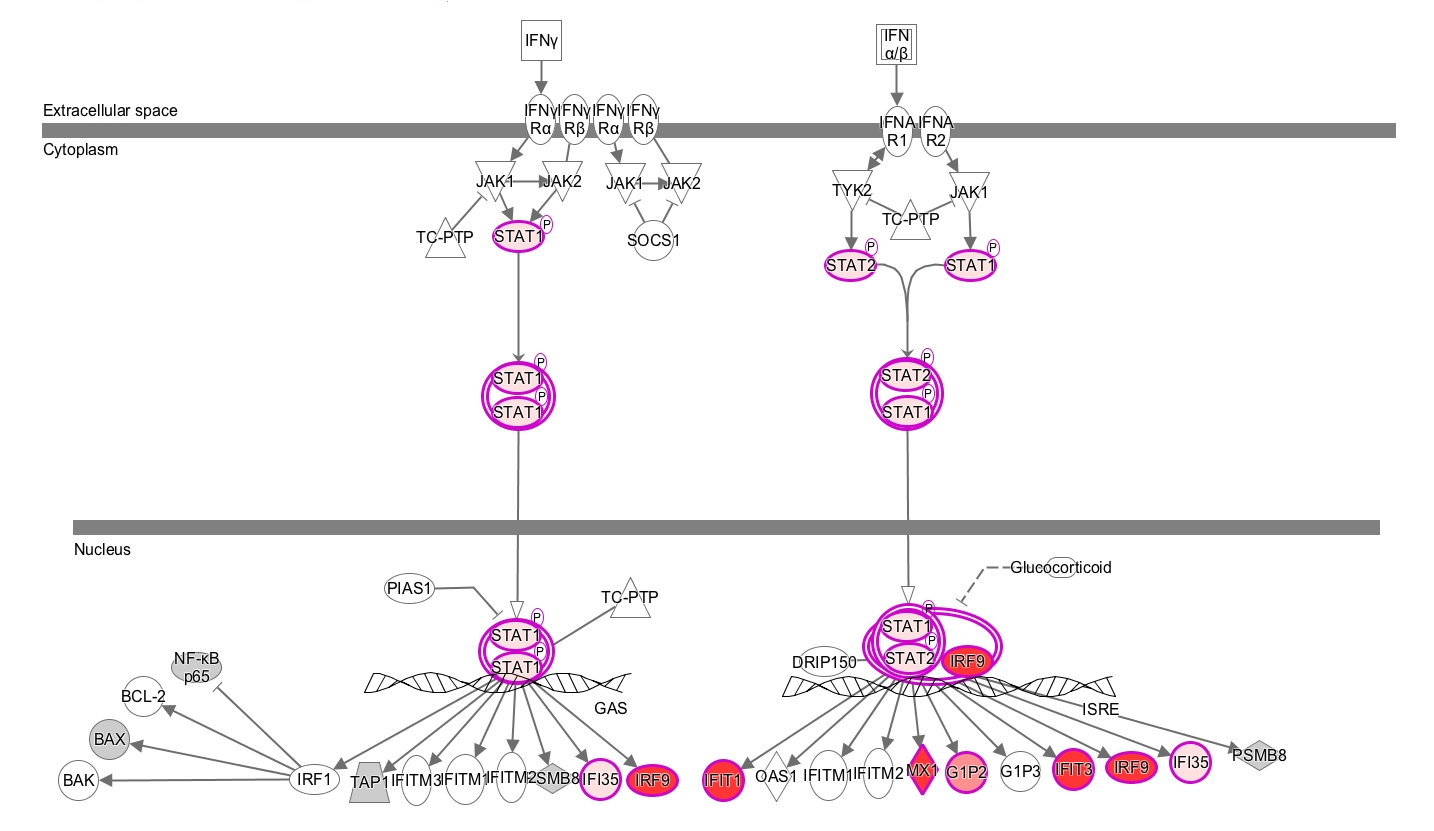
**

**A.**

**B.**

**
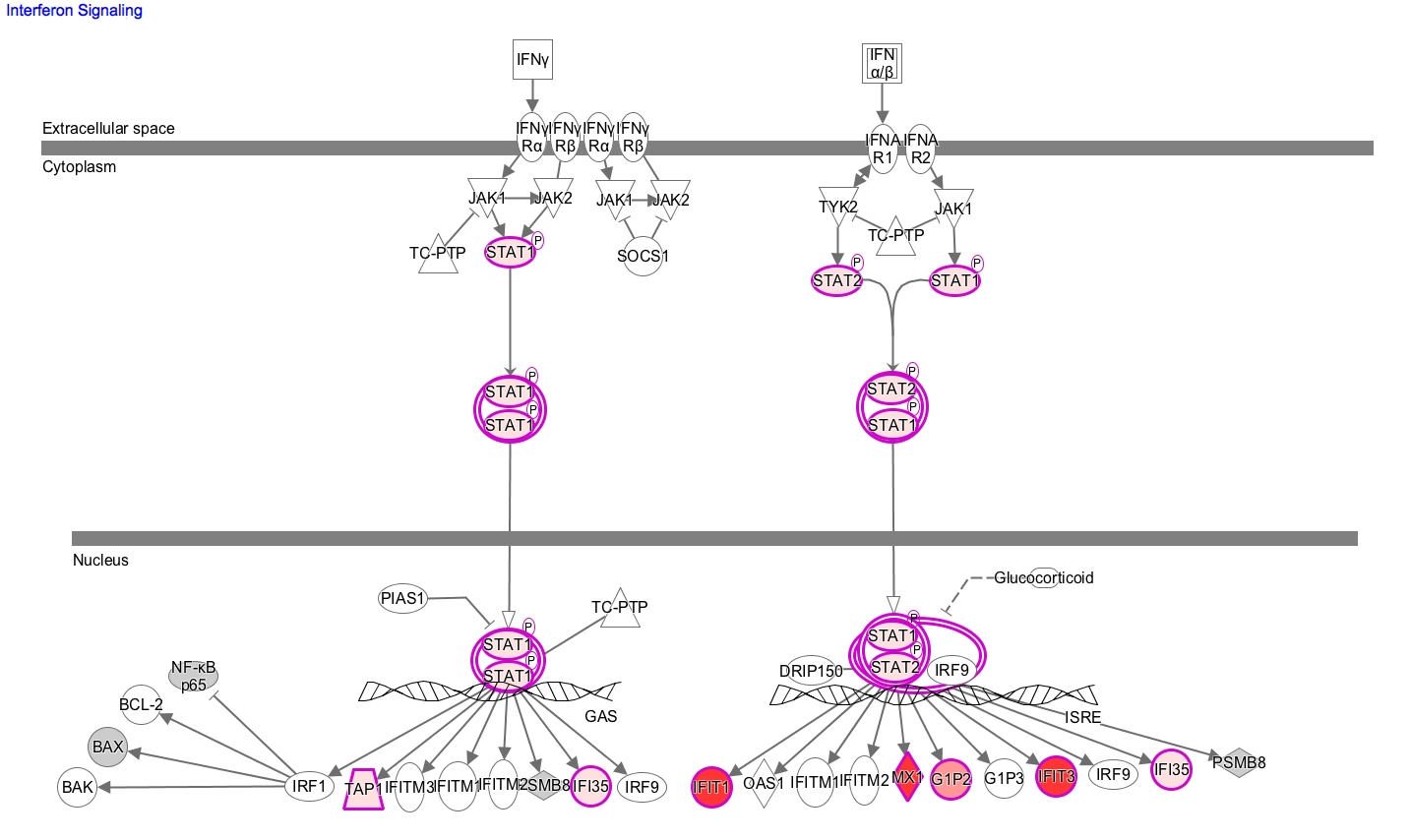
**

**Figure S10.** Similarities and differences in proteins identified in Interferon Signaling Pathway in the treated fibroblasts. **(A)** Proteins enriched from our data (colored nodes) following 100 µM c-di-AMP treatment. **(B)** Proteins enriched from our data (colored nodes) following 100 µM c-di-GMP treatment. Intensity of the red nodes signify the relative level of expression of the upregulated proteins. The functional analyses and enrichment were generated by IPA (QIAGEN Inc., https://www.qiagenbioinformatics.com/products/ingenuity-pathway-analysis)

**Table S1.** List of proteins shown in Venn diagram (Figure 2A) and their distribution in various groups following treatment with either 100 µM c-di-AMP or c-di-GMP

| S/No | Treatment/Venn Diagram group | Protein name |
| --- | --- | --- |
| i. | Proteins included exclusively in 100 µM c-di-AM treated cells | Tropomyosin alpha-3 chain |
|  | | Dynein heavy chain 3; axonemal (Axonemal beta dynein heavy chain 3) (HsADHC3) (Ciliary dynein heavy chain 3) (Dnahc3-b) |
|  |  | Histone deacetylase complex subunit SAP18 |
|  |  | Regulator of microtubule dynamics protein 1 (RMD-1) (hRMD-1) (Protein FAM82B) |
|  |  | Interferon regulatory factor 9 (IRF-9) (IFN-alpha-responsive transcription factor subunit) (ISGF3 p48 subunit) (Interferon-stimulated gene factor 3 gamma) (ISGF-3 gamma) (Transcriptional regulator ISGF3 subunit gamma) |
|  |  | cDNA FLJ60546; highly similar to Homo sapiens bridging integrator 1 (BIN1); transcript variant 3; mRNA |
|  |  | Geranylgeranyl transferase type-2 subunit beta |
|  |  | Epidermal growth factor receptor kinase substrate 8-like protein 2 |
|  |  | Branched-chain-amino-acid aminotransferase (EC 2.6.1.42) |
|  |  | RNA binding motif protein 28 isoform 1 (RNA binding motif protein 28; isoform CRA_a) |
|  |  | ARHGAP35 (Fragment) |
|  |  | Inactive tyrosine-protein kinase PEAK1 (Pseudopodium-enriched atypical kinase 1) (Sugen kinase 269) (Tyrosine-protein kinase SgK269) |
|  |  | Nuclear cap-binding protein subunit 2 (20 kDa nuclear cap-binding protein) (Cell proliferation-inducing gene 55 protein) (NCBP 20 kDa subunit) (CBP20) (NCBP-interacting protein 1) (NIP1) |
|  |  | Vacuolar protein sorting-associated protein 37B (Fragment) |
|  |  |  |
| ii. | Proteins included exclusively in 100 µM c-di-GMP treated cells | Thyroid receptor-interacting protein 11 (TR-interacting protein 11) (TRIP-11) (Clonal evolution-related gene on chromosome 14 protein) (Golgi-associated microtubule-binding protein 210) (GMAP-210) (Trip230) |
|  | | Amyloid-beta precursor protein |
|  |  | Filamin A-interacting protein 1-like (130 kDa GPBP-interacting protein) (90 kDa GPBP-interacting protein) (Protein down-regulated in ovarian cancer 1) (DOC-1) |
|  |  | Protein MGARP (Corneal endothelium-specific protein 1) (CESP-1) (Hypoxia up-regulated mitochondrial movement regulator protein) (Mitochondria-localized glutamic acid-rich protein) (Ovary-specific acidic protein) |
|  |  | 39S ribosomal protein L53; mitochondrial (L53mt) (MRP-L53) (Mitochondrial large ribosomal subunit protein mL53) |
|  |  | 3-hydroxy-3-methylglutaryl coenzyme A synthase (HMG-CoA synthase) (EC 2.3.3.10) |
|  |  | Collagen triple helix repeat-containing protein 1 (Protein NMTC1) |
|  |  | E3 ubiquitin-protein ligase DTX3L (EC 2.3.2.27) (B-lymphoma- and BAL-associated protein) (Protein deltex-3-like) (RING-type E3 ubiquitin transferase DTX3L) (Rhysin-2) (Rhysin2) |
|  |  | H. sapiens ras-related Hrab4 protein |
|  |  | Cyclin G-associated kinase variant (Fragment) |
|  |  | Neuropilin-1 variant (Fragment) |
|  |  | Membrane metallo-endopeptidase |
|  |  | 39S ribosomal protein L24; mitochondrial (L24mt) (MRP-L24) (Mitochondrial large ribosomal subunit protein uL24m) |
|  |  | Receptor-interacting serine/threonine-protein kinase 2 (EC 2.7.11.1) (Tyrosine-protein kinase RIPK2) |
|  |  | Interferon-induced GTP-binding protein Mx2 (Interferon-regulated resistance GTP-binding protein MxB) (Myxovirus resistance protein 2) (p78-related protein) |
|  |  |  |
| iii. | Proteins included exclusively in control cells | Mothers against decapentaplegic homolog (MAD homolog) (Mothers against DPP homolog) (SMAD family member) (Fragment) |
|  | | High mobility group AT-hook 1 |
|  |  | Actin; alpha 2; smooth muscle; aorta (Actin; alpha 2; smooth muscle; aorta; isoform CRA_a) |
|  |  | Contactin-associated protein 1 (Caspr) (Caspr1) (Neurexin IV) (Neurexin-4) (p190) |
|  |  | Chromobox homolog 5 (HP1 alpha homolog; Drosophila); isoform CRA_b (Epididymis luminal protein 25) |
|  |  | Chromosome-associated kinesin KIF4A (Chromokinesin-A) |
|  |  | Translocon-associated protein subunit gamma |
|  |  | Protein PRR14L (Proline rich 14-like protein) |
|  |  | 60S ribosomal protein L36a-like (Large ribosomal subunit protein eL42-like) |
|  |  | GTPBP4 protein (Fragment) |
|  |  | Small nuclear ribonucleoprotein G (snRNP-G) |
|  |  | Tumor protein p53-inducible protein 11 (Fragment) |
|  |  | Sushi domain containing 2; isoform CRA_a (Testicular tissue protein Li 190) |
|  |  | Transmembrane protein 14C variant (Fragment) |
|  |  | Transmembrane protein 205 (Fragment) |
|  |  | Beta-4 tubulin (Fragment) |
|  |  | LIM and senescent cell antigen-like-containing domain protein 1 (Particularly interesting new Cys-His protein 1) (PINCH-1) (Renal carcinoma antigen NY-REN-48) |
|  |  | cDNA FLJ50574; highly similar to ATP-dependent RNA helicase DDX24 |
|  |  | Protein C10 |
|  |  | U6 snRNA-associated Sm-like protein LSm7 |
|  |  | cDNA FLJ50501; highly similar to GRAM domain-containing protein 3 |
|  |  | WD repeat-containing protein 5 (BMP2-induced 3-kb gene protein) |
|  |  | Acyl-protein thioesterase 1 (Fragment) |
|  |  | Secretory carrier-associated membrane protein (Secretory carrier membrane protein) |
|  |  | Methylosome subunit pICln |
|  |  | Transforming acidic coiled-coil-containing protein 3 |
|  |  | Sodium/calcium exchanger 1 |
|  |  | Ribulose-phosphate 3-epimerase (EC 5.1.3.1) (Ribulose-5-phosphate-3-epimerase) |
|  |  | FLJ00144 protein (Fragment) |
|  |  | Palmitoyl-protein thioesterase 1 |
|  |  | Cyclin-dependent kinases regulatory subunit |
|  |  | cDNA; FLJ96555 |
|  |  | Neuronal protein |
|  |  | N(4)-(beta-N-acetylglucosaminyl)-L-asparaginase (EC 3.5.1.26) (Aspartylglucosaminidase) (Glycosylasparaginase) (N4-(N-acetyl-beta-glucosaminyl)-L-asparagine amidase) [Cleaved into: Glycosylasparaginase alpha chain; Glycosylasparaginase beta chain] |
|  |  | Transcription elongation factor A protein-like 3 (TCEA-like protein 3) (Transcription elongation factor S-II protein-like 3) |
|  |  | Gamma-tubulin complex component 3 (GCP-3) (hGCP3) (Gamma-ring complex protein 104 kDa) (h104p) (hGrip104) (Spindle pole body protein Spc98 homolog) (hSpc98) |
|  |  | Proto-oncogene tyrosine-protein kinase Src (EC 2.7.10.2) (Proto-oncogene c-Src) (pp60c-src) (p60-Src) |
|  |  | Cleft lip and palate associated transmembrane protein 1 isoform 2 (Cleft lip and palate associated transmembrane protein 1; isoform CRA_b) |
|  |  | Transcription activator BRG1 |
|  |  | Diacylglycerol kinase (DAG kinase) (EC 2.7.1.107) |
|  |  | cDNA FLJ52943; highly similar to Zinc transporter SLC39A7 |
|  |  | Phosphofurin acidic cluster sorting protein 1; isoform CRA_a |
|  |  | Zinc finger; NFX1-type containing 1; isoform CRA_b |
|  |  | Peripheral plasma membrane protein CASK |
|  |  | cDNA FLJ78024 |
|  |  | cDNA FLJ58024; highly similar to NADH-ubiquinone oxidoreductase 20 kDa subunit; mitochondrial |
|  |  | Torsin-4A (Torsin family 4 member A) |
|  |  | Protein LBH |
|  |  | Protein bicaudal D homolog 2 (Bic-D 2) |
|  |  | Activating signal cointegrator 1 complex subunit 3 (EC 3.6.4.12) (ASC-1 complex subunit p200) (ASC1p200) (Helicase; ATP binding 1) (Trip4 complex subunit p200) |
|  |  | Nuclear pore complex protein Nup214 |
|  |  | cDNA; FLJ96877 |
|  |  | cDNA FLJ46798 fis; clone TRACH3031660; highly similar to cAMP-dependent protein kinase type II-beta regulatory subunit |
|  |  | Ribonucloprotein (Fragment) |
|  |  | Ribosome biogenesis protein WDR12 (WD repeat-containing protein 12) |
|  |  | Starch-binding domain-containing protein 1 (Genethonin-1) (Glycophagy cargo receptor STBD1) |
|  |  | Ribosome biogenesis regulatory protein homolog |
|  |  | Ceramide synthase 2 (Fragment) |
|  |  | Protein pelota homolog (EC 3.1.-.-) |
|  |  | Oxysterol-binding protein-related protein 3 (ORP-3) (OSBP-related protein 3) |
|  |  | C-terminal-binding protein 2 (CtBP2) |
|  |  | 3-hydroxymethyl-3-methylglutaryl-Coenzyme A lyase (Hydroxymethylglutaricaciduria); isoform CRA_b (cDNA FLJ16378 fis; clone TKIDN2016399; highly similar to Hydroxymethylglutaryl-CoA lyase; mitochondrial) |
|  |  | Ankyrin-1 (ANK-1) (Ankyrin-R) (Erythrocyte ankyrin) |
|  |  | Core-binding factor; beta subunit; isoform CRA_b |
|  |  | Large neutral amino acids transporter small subunit 1 (4F2 light chain) (4F2 LC) (4F2LC) (CD98 light chain) (Integral membrane protein E16) (E16) (L-type amino acid transporter 1) (hLAT1) (Solute carrier family 7 member 5) (y+ system cationic amino acid transporter) |
|  |  | Dihydrofolate reductase (EC 1.5.1.3) (cDNA; FLJ93028; Homo sapiens dihydrofolate reductase (DHFR); mRNA) |
|  |  | Piezo-type mechanosensitive ion channel component 1 (Membrane protein induced by beta-amyloid treatment) (Mib) (Protein FAM38A) |
|  |  | ADP-ribosylation factor GTPase-activating protein 2 (Fragment) |
|  |  | Methionine aminopeptidase (EC 3.4.11.18) |
|  |  | E3 ubiquitin-protein ligase TRIP12 (EC 2.3.2.26) (E3 ubiquitin-protein ligase for Arf) (ULF) (HECT-type E3 ubiquitin transferase TRIP12) (Thyroid receptor-interacting protein 12) (TR-interacting protein 12) (TRIP-12) |
|  |  | TXNRD2 protein |
|  |  | Methylmalonyl-CoA mutase variant c.613_615delGAA |
|  |  | Splicing factor 3B subunit 5 (SF3b5) (Pre-mRNA-splicing factor SF3b 10 kDa subunit) |
|  |  | Retinal rod rhodopsin-sensitive cGMP 3';5'-cyclic phosphodiesterase subunit delta |
|  |  | [F-actin]-monooxygenase MICAL2 |
|  |  | cDNA FLJ57657; highly similar to Charged multivesicular body protein 1a |
|  |  | MKI67 FHA domain-interacting nucleolar phosphoprotein (Nucleolar phosphoprotein Nopp34) (Nucleolar protein interacting with the FHA domain of pKI-67) (hNIFK) |
|  |  | Mitochondrial import inner membrane translocase subunit Tim23 |
|  |  | cDNA FLJ57133; highly similar to Bifunctional purine biosynthesis protein PURH |
|  |  | ATPase family AAA domain-containing protein 1 (EC 3.6.1.3) (Thorase) |
|  |  | Guanine nucleotide-binding protein-like 3 (E2-induced gene 3 protein) (Novel nucleolar protein 47) (NNP47) (Nucleolar GTP-binding protein 3) (Nucleostemin) |
|  |  | Actin filament-associated protein 1 (110 kDa actin filament-associated protein) (AFAP-110) |
|  |  | Golgi SNAP receptor complex member 2 (Fragment) |
|  |  | 39S ribosomal protein L4; mitochondrial (Fragment) |
|  |  | Chromosome 10 open reading frame 70; isoform CRA_b |
|  |  | Glycerol-3-phosphate phosphatase (G3PP) (EC 3.1.3.21) (Aspartate-based ubiquitous Mg(2+)-dependent phosphatase) (AUM) (EC 3.1.3.48) (Phosphoglycolate phosphatase) (PGP) |
|  |  | SGCB protein |
|  |  | NADH dehydrogenase (Ubiquinone) 1 beta subcomplex; 3; 12kDa; isoform CRA_a |
|  |  | cDNA FLJ54538; highly similar to Transportin-2 |
|  |  | Serine/threonine-protein kinase N2 (EC 2.7.11.13) (PKN gamma) (Protein kinase C-like 2) (Protein-kinase C-related kinase 2) |
|  |  | Lysophospholipid acyltransferase 7 (Fragment) |
|  |  | ADAM10 protein |
|  |  | cDNA FLJ56344; highly similar to Implantation-associated protein |
|  |  | E3 ubiquitin-protein ligase MARCH5 (EC 2.3.2.27) (Membrane-associated RING finger protein 5) (Membrane-associated RING-CH protein V) (MARCH-V) (Mitochondrial ubiquitin ligase) (MITOL) (RING finger protein 153) (RING-type E3 ubiquitin transferase MARCH5) |
|  |  | cDNA FLJ75882; highly similar to Homo sapiens spastic paraplegia 20; spartin (Troyer syndrome) (SPG20); mRNA |
|  |  | cDNA FLJ37014 fis; clone BRACE2010203; highly similar to Zinc phosphodiesterase ELAC protein 2 |
|  |  | cDNA; FLJ93255; highly similar to Homo sapiens solute carrier family 7 (cationic amino acidtransporter; y+ system); member 1 (SLC7A1); mRNA |
|  |  | Sorting nexin-27 |
|  |  | cDNA PSEC0119 fis; clone PLACE1002376; highly similar to GPI transamidase component PIG-S |
|  |  | Endothelial protein C receptor (Fragment) |
|  |  | cDNA FLJ56545; highly similar to ATP-dependent RNA helicase DDX50 |
|  |  | Pyruvate carboxylase (EC 6.4.1.1) |
|  |  | CCR4-NOT transcription complex; subunit 3; isoform CRA_a |
|  |  | Active breakpoint cluster region-related protein (cDNA FLJ54747; highly similar to Active breakpoint cluster region-related protein) |
|  |  | cDNA FLJ78524; highly similar to Homo sapiens SMILE protein (SMILE); mRNA |
|  |  | FYVE and coiled-coil domain-containing protein 1 (Zinc finger FYVE domain-containing protein 7) |
|  |  | ADP-ribosylation factor-like protein 6-interacting protein 4 (Fragment) |
|  |  | cDNA FLJ31479 fis; clone NT2NE2001634; moderately similar to NADH-UBIQUINONE OXIDOREDUCTASE 9 KD SUBUNIT |
|  |  | ER lumen protein-retaining receptor 1 (KDEL endoplasmic reticulum protein retention receptor 1) (KDEL receptor 1) (Putative MAPK-activating protein PM23) |
|  |  | Lysophosphatidylserine lipase ABHD12 (EC 3.1.-.-) (2-arachidonoylglycerol hydrolase ABHD12) (Abhydrolase domain-containing protein 12) (hABHD12) (Monoacylglycerol lipase ABHD12) (EC 3.1.1.23) (Oxidized phosphatidylserine lipase ABHD12) (EC 3.1.-.-) |
|  |  | cDNA FLJ50791; highly similar to Nitrilase homolog 1 |
|  |  | Replication factor C subunit 4 (Activator 1 37 kDa subunit) (A1 37 kDa subunit) (Activator 1 subunit 4) (Replication factor C 37 kDa subunit) (RF-C 37 kDa subunit) (RFC37) |
|  |  | Pumilio homolog 1 (Fragment) |
|  |  | Golgi-associated PDZ and coiled-coil motif-containing protein (CFTR-associated ligand) (Fused in glioblastoma) (PDZ protein interacting specifically with TC10) (PIST) |
|  |  | Apolipoprotein B mRNA editing enzyme catalytic polypeptide-like 3B (Apolipoprotein B mRNA editing enzyme; catalytic polypeptide-like 3B; isoform CRA_c) |
|  |  | TBC1 domain family member 17 (Fragment) |
|  |  | Tyrosine-protein kinase BAZ1B (EC 2.7.10.2) (Bromodomain adjacent to zinc finger domain protein 1B) (Williams syndrome transcription factor) (Williams-Beuren syndrome chromosomal region 10 protein) (Williams-Beuren syndrome chromosomal region 9 protein) (hWALp2) |
|  |  | Peroxisomal carnitine O-octanoyltransferase |
|  |  | Protein phosphatase 1; regulatory (Inhibitor) subunit 8 |
|  |  | cDNA FLJ56289; highly similar to Homo sapiens sperm specific antigen 2 (SSFA2); mRNA |
|  |  | Mitochondrial ribosomal protein S7; isoform CRA_a |
|  |  | Protein regulator of cytokinesis 1; isoform CRA_e |
|  |  | Nesprin-3 (KASH domain-containing protein 3) (KASH3) (Nuclear envelope spectrin repeat protein 3) |
|  |  | Torsin-1B (Torsin ATPase-1B) (EC 3.6.4.-) (Torsin family 1 member B) |
|  |  | cDNA FLJ56673; highly similar to Homo sapiens adipocyte-specific adhesion molecule (ASAM); mRNA |
|  |  | Solute carrier family 25 member 4 isoform 1 (Fragment) |
|  |  | Protein ABHD11 (EC 3.-.-.-) (Alpha/beta hydrolase domain-containing protein 11) (Abhydrolase domain-containing protein 11) (Williams-Beuren syndrome chromosomal region 21 protein) |
|  |  | cDNA FLJ60304; highly similar to Rab GTPase-binding effector protein 1 |
|  |  | ER membrane protein complex subunit 3 (Transmembrane protein 111) |
|  |  | EH domain-binding protein 1-like protein 1 |
|  |  | Kinesin-like protein KIF20A (GG10_2) (Mitotic kinesin-like protein 2) (MKlp2) (Rab6-interacting kinesin-like protein) (Rabkinesin-6) |
|  |  | Cyclin-dependent kinase 5 isoform 2 (Fragment) |
|  |  | Methyltransferase-like 26 |
|  |  | cDNA FLJ57941; highly similar to Nucleoside diphosphate-linked moiety X motif16 |
|  |  | 39S ribosomal protein L13; mitochondrial (L13mt) (MRP-L13) (Mitochondrial large ribosomal subunit protein uL13m) |
|  |  | RUN and FYVE domain-containing protein 1 (FYVE-finger protein EIP1) (La-binding protein 1) (Rab4-interacting protein) (Zinc finger FYVE domain-containing protein 12) |
|  |  | Fibronectin type III domain containing 3A; isoform CRA_b |
|  |  | DOCK5 (Fragment) |
|  |  | Mitotic-spindle organizing protein 2B |
|  |  | Serine palmitoyltransferase; long chain base subunit 2; isoform CRA_a |
|  |  | cDNA FLJ75725; highly similar to Homo sapiens vesicle transport through interaction with t-SNAREs homolog 1B (yeast) (VTI1B); mRNA |
|  |  | cDNA; FLJ94423; highly similar to Homo sapiens mitochondrial ribosomal protein L23 (MRPL23); nuclear gene encoding mitochondrial protein; mRNA |
|  |  | cDNA FLJ75454; highly similar to Homo sapiens arrestin; beta 1 (ARRB1); transcript variant 1; mRNA |
|  |  | cDNA FLJ76707; highly similar to Homo sapiens transmembrane protein 87A; mRNA |
|  |  | Ribosome production factor 2 homolog (Fragment) |
|  |  | MRPL1 protein (Fragment) |
|  |  | Cytidine 5'-monophosphate N-acetylneuraminic acid synthetase variant (Fragment) |
|  |  | Microtubule-associated proteins 1A/1B light chain 3A (Autophagy-related protein LC3 A) (Autophagy-related ubiquitin-like modifier LC3 A) (MAP1 light chain 3-like protein 1) (MAP1A/MAP1B light chain 3 A) (MAP1A/MAP1B LC3 A) (Microtubule-associated protein 1 light chain 3 alpha) |
|  |  | Uncharacterized protein DKFZp686E23276 (Fragment) |
|  |  | cDNA FLJ37759 fis; clone BRHIP2023888 |
|  |  | Carboxypeptidase D (EC 3.4.17.22) (Metallocarboxypeptidase D) (gp180) |
|  |  | Abl interactor 2 |
|  |  | Uridine-cytidine kinase 2 |
|  |  | C-Mpl binding protein (La-related protein 4) |
|  |  | MRPS9 protein (Fragment) |
|  |  | Bax inhibitor 1 (Fragment) |
|  |  | Plasma alpha-L-fucosidase (EC 3.2.1.51) (Alpha-L-fucoside fucohydrolase 2) (Alpha-L-fucosidase 2) |
|  |  | Aldehyde dehydrogenase 1 family member L1 isoform 3 (Fragment) |
|  |  | Sorting nexin-4 |
|  |  | Retinol dehydrogenase 14 (All-trans and 9-cis); isoform CRA_a (cDNA; FLJ95921; Homo sapiens retinol dehydrogenase 14 (all-trans and 9-cis)(RDH14); mRNA) |
|  |  | Tumor necrosis factor receptor type 1-associated DEATH domain protein (TNFR1-associated DEATH domain protein) (TNFRSF1A-associated via death domain) |
|  |  | 3-hydroxyisobutyryl-CoA hydrolase; mitochondrial (EC 3.1.2.4) (3-hydroxyisobutyryl-coenzyme A hydrolase) |
|  |  | Structural maintenance of chromosomes flexible hinge domain-containing protein 1 (SMC hinge domain-containing protein 1) (EC 3.6.1.-) |
|  |  | DNA repair protein XRCC1 |
|  |  | 28S ribosomal protein S17; mitochondrial (Fragment) |
|  |  | CGI-111 protein |
|  |  | Putative hydroxypyruvate isomerase (Fragment) |
|  |  | Carboxymethylenebutenolidase homolog (EC 3.1.-.-) |
|  |  | cDNA FLJ60156; highly similar to Vacuolar protein sorting protein 52 |
|  |  | Phosphorylated adapter RNA export protein (RNA U small nuclear RNA export adapter protein) |
|  |  | Carboxypeptidase (EC 3.4.16.-) |
|  |  | E3 ubiquitin-protein ligase RNF114 (Fragment) |
|  |  | 39S ribosomal protein L38; mitochondrial (L38mt) (MRP-L38) (Mitochondrial large ribosomal subunit protein mL38) |
|  |  | Frequenin homolog (Drosophila); isoform CRA_a |
|  |  | cDNA FLJ75682; highly similar to Homo sapiens armadillo repeat containing; X-linked 3 (ARMCX3); transcript variant 1; mRNA |
|  |  | Mitochondrial Rho GTPase 2 (MIRO-2) (hMiro-2) (EC 3.6.5.-) (Ras homolog gene family member T2) |
|  |  | Mitochondrial ribosomal protein S2; isoform CRA_a |
|  |  | DNAJC1 protein (Fragment) |
|  |  | Mannose-P-dolichol utilization defect 1 protein |
|  |  | 28S ribosomal protein S23; mitochondrial |
|  |  | cDNA FLJ55135; highly similar to Di-N-acetylchitobiase |
|  |  | E3 ubiquitin-protein ligase RNF181 |
|  |  | Queuosine salvage protein |
|  |  | Processing of 1; ribonuclease P/MRP subunit (S. cerevisiae) |
|  |  | Telomere length regulation protein TEL2 homolog (Protein clk-2 homolog) (hCLK2) |
|  |  |  |
| iv. | Common proteins in 100 µM c-di-AMP treated and control cells | Hepatopoietin PCn127 |
|  | | RPLP1 protein |
|  |  | Protein disulfide-isomerase A3 (Fragment) |
|  |  | Titin |
|  |  | Tetraspanin |
|  |  | Tropomyosin 1 (Alpha); isoform CRA_a |
|  |  | Pumilio homolog 3 (HBV X-transactivated gene 5 protein) (HBV XAg-transactivated protein 5) (Minor histocompatibility antigen HA-8) (HLA-HA8) |
|  |  | cDNA; FLJ79464; highly similar to Homo sapiens aquarius (Fragment) |
|  |  | Telomerase RNA component interacting RNase (EC 3.1.13.-) (Exoribonuclease TRIR) |
|  |  | Eukaryotic translation elongation factor 1 alpha (Fragment) |
|  |  | Cytochrome b-c1 complex subunit 8 (Complex III subunit 8) (Complex III subunit VIII) (Ubiquinol-cytochrome c reductase complex 9.5 kDa protein) (Ubiquinol-cytochrome c reductase complex ubiquinone-binding protein QP-C) |
|  |  | Golgin subfamily B member 1 (372 kDa Golgi complex-associated protein) (GCP372) (Giantin) (Macrogolgin) |
|  |  | NADH dehydrogenase (Ubiquinone) 1 alpha subcomplex; 3; 9kDa; isoform CRA_e (NADH dehydrogenase [ubiquinone] 1 alpha subcomplex subunit 3) (NDUFA3 protein) (cDNA FLJ76508; highly similar to Homo sapiens NADH dehydrogenase (ubiquinone) 1 alpha subcomplex; 3; 9kDa (NDUFA3); mRNA) |
|  |  | Lysophospholipase II; isoform CRA_f (Testicular tissue protein Li 3) |
|  |  | cAMP-regulated phosphoprotein 19 |
|  |  | cDNA FLJ42854 fis; clone BRHIP2008607; highly similar to Breast cancer anti-estrogen resistance protein 1 |
|  |  | Kinesin-like protein KIF11 (Kinesin-like protein 1) (Kinesin-like spindle protein HKSP) (Kinesin-related motor protein Eg5) (Thyroid receptor-interacting protein 5) (TR-interacting protein 5) (TRIP-5) |
|  |  | NADH dehydrogenase (Ubiquinone) 1 alpha subcomplex; 4; 9kDa; isoform CRA_b |
|  |  | Cytochrome b-c1 complex subunit 9 (Complex III subunit 9) (Complex III subunit X) (Cytochrome c1 non-heme 7 kDa protein) (Ubiquinol-cytochrome c reductase complex 7.2 kDa protein) |
|  |  | RAP1A; member of RAS oncogene family |
|  |  | Condensin complex subunit 1 |
|  |  | Deoxyribose-phosphate aldolase (DERA) (EC 4.1.2.4) (2-deoxy-D-ribose 5-phosphate aldolase) (Phosphodeoxyriboaldolase) (Deoxyriboaldolase) |
|  |  | Calcium-binding protein 39 |
|  |  | Heat shock 27kDa protein family; member 7 (Cardiovascular); isoform CRA_c (Heat shock protein beta-7) (cDNA FLJ34956 fis; clone NTONG2003158; highly similar to Heat shock 27kD protein family; member 7) |
|  |  | DUF1681 domain-containing protein (Fragment) |
|  |  | Acylglycerol kinase; mitochondrial |
|  |  | Protein phosphatase inhibitor 2 family member B (PPP1R2 family member B) (Protein phosphatase 1; regulatory subunit 2 pseudogene 3) (Protein phosphatase inhibitor 2-like protein 3) |
|  |  | ATP-dependent Clp protease proteolytic subunit; mitochondrial (EC 3.4.21.92) (Endopeptidase Clp) |
|  |  | SHC-transforming protein 1 |
|  |  | Exocyst complex component 5 (Exocyst complex component Sec10) (hSec10) |
|  |  | Exocyst complex component 8 (Exocyst complex 84 kDa subunit) |
|  |  | GPI ethanolamine phosphate transferase 1 |
|  |  | ATPase family AAA domain-containing protein 3A (Fragment) |
|  |  | UBE2C protein (Ubiquitin-conjugating enzyme E2C) (Ubiquitin-conjugating enzyme E2C; isoform CRA_e) |
|  |  | NADH dehydrogenase [ubiquinone] 1 beta subcomplex subunit 9 |
|  |  | Exocyst complex component 2 |
|  |  | Serine/threonine-protein phosphatase PGAM5; mitochondrial (EC 3.1.3.16) (Bcl-XL-binding protein v68) (Phosphoglycerate mutase family member 5) |
|  |  | rRNA/tRNA 2'-O-methyltransferase fibrillarin-like protein 1 (EC 2.1.1.-) (Protein-glutamine methyltransferase) |
|  |  | Replication factor C subunit 2 (Activator 1 40 kDa subunit) (A1 40 kDa subunit) (Activator 1 subunit 2) (Replication factor C 40 kDa subunit) (RF-C 40 kDa subunit) (RFC40) |
|  |  | Selenide; water dikinase 1 (EC 2.7.9.3) (Selenium donor protein 1) (Selenophosphate synthase 1) |
|  |  | Chromosome 11 open reading frame 58 |
|  |  | HCG2003792; isoform CRA_b |
|  |  | DNA repair protein RAD50 (hRAD50) (EC 3.6.-.-) |
|  |  | Pleckstrin homology-like domain; family B; member 1; isoform CRA_c |
|  |  | Ras-related protein Rab-34; isoform NARR (Fragment) |
|  |  | Rho guanine nucleotide exchange factor (GEF) 7; isoform CRA_b |
|  |  | V-type proton ATPase 16 kDa proteolipid subunit (V-ATPase 16 kDa proteolipid subunit) (Vacuolar proton pump 16 kDa proteolipid subunit) |
|  |  | Nudix (Nucleoside diphosphate linked moiety X)-type motif 1; isoform CRA_a |
|  |  | Esterase OVCA2 (EC 3.1.2.-) (Ovarian cancer-associated gene 2 protein) |
|  |  | Glycylpeptide N-tetradecanoyltransferase (EC 2.3.1.97) |
|  |  | Ubiquitin-conjugating enzyme E2 A |
|  |  | Protein diaphanous homolog 3 (Diaphanous-related formin-3) (DRF3) (MDia2) |
|  |  | Integrin alpha-2 |
|  |  | cDNA FLJ56802; highly similar to LanC-like protein 2 |
|  |  | Neurabin-2 (Neurabin-II) (Protein phosphatase 1 regulatory subunit 9B) (Spinophilin) |
|  |  | Protein MEMO1 (C21orf19-like protein) (Hepatitis C virus NS5A-transactivated protein 7) (HCV NS5A-transactivated protein 7) (Mediator of ErbB2-driven cell motility 1) (Mediator of cell motility 1) (Memo-1) |
|  |  | AP2-associated protein kinase 1 (Fragment) |
|  |  | Nucleoporin 153kDa; isoform CRA_a |
|  |  | Wolfram syndrome 1 isoform 1 (Fragment) |
|  |  | U6 snRNA-associated Sm-like protein LSm1 (Fragment) |
|  |  | 26S proteasome non-ATPase regulatory subunit 10 |
|  |  | Rab3 GTPase-activating protein non-catalytic subunit (RGAP-iso) (Rab3 GTPase-activating protein 150 kDa subunit) (Rab3-GAP p150) (Rab3-GAP150) (Rab3-GAP regulatory subunit) |
|  |  | Calcium uniporter protein; mitochondrial (HsMCU) (Coiled-coil domain-containing protein 109A) |
|  |  | Lamin B receptor; isoform CRA_a |
|  |  | cDNA FLJ51512; highly similar to Periodic tryptophan protein 1 homolog |
|  |  | cDNA FLJ56173; highly similar to U4/U6.U5 tri-snRNP-associated protein 2 |
|  |  | UBX domain-containing protein 6 (UBX domain-containing protein 1) |
|  |  | Phosphopantothenate--cysteine ligase (EC 6.3.2.51) (Phosphopantothenoylcysteine synthetase) (PPC synthetase) |
|  |  | Disks large homolog 1 |
|  |  | GPR177 protein (Fragment) |
|  |  | Actin-like protein (Fragment) |
|  |  | DNA mismatch repair protein Msh6 (hMSH6) (G/T mismatch-binding protein) (GTBP) (GTMBP) (MutS protein homolog 6) (MutS-alpha 160 kDa subunit) (p160) |
|  |  | CCR4-NOT transcription complex subunit 1 (CCR4-associated factor 1) (Negative regulator of transcription subunit 1 homolog) (NOT1H) (hNOT1) |
|  |  | Complex I assembly factor ACAD9; mitochondrial (Acyl-CoA dehydrogenase family member 9) (ACAD-9) (EC 1.3.8.-) |
|  |  | Syntaxin 5A; isoform CRA_c (cDNA FLJ57518; highly similar to Syntaxin-5) |
|  |  | Condensin complex subunit 2 |
|  |  | Molybdopterin synthase catalytic subunit (EC 2.8.1.12) (Molybdenum cofactor synthesis protein 2 large subunit) (Molybdenum cofactor synthesis protein 2B) (MOCS2B) |
|  |  | Mothers against decapentaplegic homolog (MAD homolog) (Mothers against DPP homolog) (SMAD family member) |
|  |  | Histone-lysine N-methyltransferase SETD7 (EC 2.1.1.-) (Histone H3-K4 methyltransferase SETD7) (H3-K4-HMTase SETD7) (Lysine N-methyltransferase 7) (SET domain-containing protein 7) (SET7/9) |
|  |  | Anoctamin-6 (Small-conductance calcium-activated nonselective cation channel) (SCAN channel) (Transmembrane protein 16F) |
|  |  | Vacuolar-sorting protein SNF8 (ELL-associated protein of 30 kDa) (ESCRT-II complex subunit VPS22) (hVps22) |
|  |  | Deoxyribonuclease II (Fragment) |
|  |  | TBC1 domain family member 23 (HCV non-structural protein 4A-transactivated protein 1) |
|  |  | Plasminogen receptor (KT) (Plg-R(KT)) |
|  |  | ClpX caseinolytic peptidase X homolog (E. coli); isoform CRA_a |
|  |  | Chitobiosyldiphosphodolichol beta-mannosyltransferase (Fragment) |
|  |  | Elongation factor Ts; mitochondrial (EF-Ts) (EF-TsMt) |
|  |  | DNA-directed RNA polymerases I; II; and III subunit RPABC1 |
|  |  | Tetratricopeptide repeat domain 1 variant (Fragment) |
|  |  | NFU1 iron-sulfur cluster scaffold homolog; mitochondrial (HIRA-interacting protein 5) |
|  |  | TOM1-like protein 2 (Target of Myb-like protein 2) |
|  |  | Translation initiation factor eIF-2B subunit gamma (eIF-2B GDP-GTP exchange factor subunit gamma) |
|  |  | TOMM7 protein (Translocase of outer mitochondrial membrane 7 homolog (Yeast)) (Translocase of outer mitochondrial membrane 7 homolog (Yeast); isoform CRA_b) |
|  |  | Kinetochore protein Spc25 (hSpc25) |
|  |  | Protein phosphatase 1; regulatory (Inhibitor) subunit 13 like; isoform CRA_a |
|  |  | RWD domain-containing protein 1 (Fragment) |
|  |  | cDNA FLJ54557; highly similar to helicase MOV-10 |
|  |  | Serine threonine kinase 39 isoform B (Fragment) |
|  |  | DIS3 |
|  |  | Gasdermin domain containing 1; isoform CRA_d (Gasdermin-D) |
|  |  | Tripartite motif-containing 32; isoform CRA_a |
|  |  | Ribosomal protein S6 kinase (EC 2.7.11.1) |
|  |  | Torsin family 1; member A (Torsin A); isoform CRA_a (cDNA FLJ56343; highly similar to Torsin A) |
|  |  | Leucine rich repeat containing 40 (Testicular tissue protein Li 109) |
|  |  | Rab GTPase-activating protein 1 (GAP and centrosome-associated protein) (Rab6 GTPase-activating protein GAPCenA) |
|  |  | Uncharacterized protein DKFZp547A0616 (Fragment) |
|  |  | Protein NipSnap homolog 2 (NipSnap2) (Glioblastoma-amplified sequence) |
|  |  | cDNA; FLJ93570; highly similar to Homo sapiens phosphoribosyl pyrophosphate synthetase-associated protein 2 (PRPSAP2); mRNA |
|  |  | DEAD-box corepressor DP103 |
|  |  | E3 ubiquitin-protein ligase Itchy homolog (Itch) (EC 2.3.2.26) (Atrophin-1-interacting protein 4) (AIP4) (HECT-type E3 ubiquitin transferase Itchy homolog) (NFE2-associated polypeptide 1) (NAPP1) |
|  |  | Nuclear pore complex protein Nup85 (85 kDa nucleoporin) (FROUNT) (Nucleoporin Nup75) (Nucleoporin Nup85) (Pericentrin-1) |
|  |  | cDNA FLJ58014; highly similar to Homo sapiens programmed cell death 4; transcript variant 1; mRNA |
|  |  | cDNA FLJ61262; highly similar to Ubiquitin-protein ligase E3C |
|  |  | Bumetanide-sensitive Na-K-Cl cotransporter (Solute carrier family 12 (Sodium/potassium/chloride transporters); member 2; isoform CRA_b) |
|  |  | Phosphoinositide phospholipase C (EC 3.1.4.11) (Fragment) |
|  |  | Mycophenolic acid acyl-glucuronide esterase; mitochondrial (EC 3.1.1.93) (Alpha/beta hydrolase domain-containing protein 10) (Abhydrolase domain-containing protein 10) |
|  |  | Inositol 1;4;5-trisphosphate receptor type 3 (IP3 receptor isoform 3) (IP3R 3) (InsP3R3) (Type 3 inositol 1;4;5-trisphosphate receptor) (Type 3 InsP3 receptor) |
|  |  | 39S ribosomal protein L39; mitochondrial (Fragment) |
|  |  | L-aminoadipate-semialdehyde dehydrogenase-phosphopantetheinyl transferase (EC 2.7.8.7) (4'-phosphopantetheinyl transferase) (Alpha-aminoadipic semialdehyde dehydrogenase-phosphopantetheinyl transferase) (AASD-PPT) (LYS5 ortholog) |
|  |  | mRNA cap guanine-N7 methyltransferase |
|  |  |  |
| v. | Common proteins in 100 µM c-di-GMP treated and control cells | Reticulon-4 (Foocen) (Neurite outgrowth inhibitor) (Nogo protein) (Neuroendocrine-specific protein) (NSP) (Neuroendocrine-specific protein C homolog) (RTN-x) (Reticulon-5) |
|  | | Protocadherin gamma-A9 (PCDH-gamma-A9) |
|  |  | Cell migration-inducing and hyaluronan-binding protein (EC 3.2.1.35) |
|  |  | cDNA FLJ34004 fis; clone FCBBF1000232; highly similar to Cytochrome P450 51A1 |
|  |  | Matrix metalloproteinase 1 preproprotein variant (Fragment) |
|  |  | ADP-ribosylation factor 5 (ADP-ribosylation factor 5; isoform CRA_a) (cDNA; FLJ92389; Homo sapiens ADP-ribosylation factor 5 (ARF5); mRNA) |
|  |  | Solute carrier family 38; member 2; isoform CRA_b |
|  |  | Copper transport protein ATOX1 |
|  |  | cDNA FLJ57046; highly similar to Lysosomal alpha-glucosidase |
|  |  | U2 snRNP-associated SURP motif-containing protein (140 kDa Ser/Arg-rich domain protein) (U2-associated protein SR140) |
|  |  | Thioredoxin-related transmembrane protein 1 (Thioredoxin domain-containing protein 1) (Transmembrane Trx-related protein) |
|  |  | Chromosome 6 open reading frame 55; isoform CRA_b (Vacuolar protein sorting-associated protein VTA1 homolog) |
|  |  | Septin-8 |
|  |  | Tripartite motif-containing 25 variant (Fragment) |
|  |  | Mitochondrial fission 1 protein (FIS1 homolog) (hFis1) (Tetratricopeptide repeat protein 11) (TPR repeat protein 11) |
|  |  | Metaxin-2 |
|  |  | Phosphatidylinositol 4-kinase type 2-alpha (EC 2.7.1.67) (Phosphatidylinositol 4-kinase type II-alpha) |
|  |  | High glucose-regulated protein 8 |
|  |  | Nesprin-1 (Enaptin) (KASH domain-containing protein 1) (KASH1) (Myocyte nuclear envelope protein 1) (Myne-1) (Nuclear envelope spectrin repeat protein 1) (Synaptic nuclear envelope protein 1) (Syne-1) |
|  |  | Histone acetyltransferase type B catalytic subunit (EC 2.3.1.48) (Histone acetyltransferase 1) |
|  |  | ADP-ribosylation factor GTPase-activating protein 1 (ARF GAP 1) (ADP-ribosylation factor 1 GTPase-activating protein) (ARF1 GAP) (ARF1-directed GTPase-activating protein) |
|  |  | cDNA FLJ61541; highly similar to Homo sapiens PDZ and LIM domain 5 (PDLIM5); transcript variant 2; mRNA |
|  |  | cDNA FLJ58827; highly similar to UBX domain-containing protein 2 |
|  |  | cDNA FLJ51641; highly similar to Nicastrin |
|  |  | 60S ribosomal protein L22-like 1 |
|  |  | cDNA FLJ60347; highly similar to Homo sapiens hydroxyacylglutathione hydrolase (HAGH); transcript variant 1; mRNA |
|  |  | E3 ubiquitin-protein ligase TRIM21 (EC 2.3.2.27) (52 kDa Ro protein) (52 kDa ribonucleoprotein autoantigen Ro/SS-A) (RING finger protein 81) (RING-type E3 ubiquitin transferase TRIM21) (Ro(SS-A)) (Sjoegren syndrome type A antigen) (SS-A) (Tripartite motif-containing protein 21) |
|  |  | CB1 cannabinoid receptor-interacting protein 1 (CRIP-1) |
|  |  | Ragulator complex protein LAMTOR2 (Endosomal adaptor protein p14) (Late endosomal/lysosomal Mp1-interacting protein) (Late endosomal/lysosomal adaptor and MAPK and MTOR activator 2) (Mitogen-activated protein-binding protein-interacting protein) (MAPBP-interacting protein) (Roadblock domain-containing protein 3) |
|  |  | Glycogen [starch] synthase (EC 2.4.1.11) |
|  |  | NEDD8-activating enzyme E1 catalytic subunit |
|  |  | Kinesin light chain 2 (KLC 2) |
|  |  | cDNA FLJ78231; highly similar to Homo sapiens TSC22 domain family; member 1 (TSC22D1); transcript variant 2; mRNA |
|  |  | Selenoprotein F |
|  |  | DCC-interacting protein 13-alpha (Dip13-alpha) (Adapter protein containing PH domain; PTB domain and leucine zipper motif 1) |
|  |  | DNA topoisomerase 2 (EC 5.6.2.2) (Fragment) |
|  |  | cDNA FLJ61635; highly similar to Homo sapiens likely ortholog of mouse immediate early response; erythropoietin 4 (LEREPO4); mRNA |
|  |  | cDNA; FLJ93607 |
|  |  | Tumor necrosis factor receptor superfamily member 11B (Osteoclastogenesis inhibitory factor) (Osteoprotegerin) |
|  |  | Nucleoporin Nup37 (p37) (Nup107-160 subcomplex subunit Nup37) |
|  |  | cDNA; FLJ96027; highly similar to Homo sapiens reversion-inducing-cysteine-rich protein with kazal motifs (RECK); mRNA |
|  |  | Trans-Golgi network integral membrane protein 2 (Trans-Golgi network glycoprotein 46) (TGN38 homolog) (hTGN46) (Trans-Golgi network glycoprotein 48) (hTGN48) (Trans-Golgi network glycoprotein 51) (hTGN51) (Trans-Golgi network protein 2) |
|  |  | Translocase of inner mitochondrial membrane 9 homolog (Yeast); isoform CRA_a |
|  |  | EF-hand domain-containing protein D1 (EF-hand domain-containing protein 1) (Swiprosin-2) |
|  |  | Retinol dehydrogenase 11 isoform 1 (Fragment) |
|  |  | Arf-GAP with SH3 domain; ANK repeat and PH domain-containing protein 1 (Development and differentiation enhancing factor 1; isoform CRA_b) |
|  |  | ER membrane protein complex subunit 8 (Fragment) |
|  |  | Ectonucleoside triphosphate diphosphohydrolase 1 (NTPDase 1) (EC 3.6.1.5) (Ecto-ATP diphosphohydrolase 1) (Ecto-ATPDase 1) (Ecto-ATPase 1) (Ecto-apyrase) (Lymphoid cell activation antigen) (CD antigen CD39) |
|  |  | Ectopic P granules protein 5 homolog |
|  |  | Mitogen-activated protein kinase kinase kinase kinase 4 (Fragment) |
|  |  | Ras-related GTP binding C; isoform CRA_a |
|  |  | Argininosuccinate lyase isoform 1 (Argininosuccinate lyase; isoform CRA_b) |
|  |  | IL6ST isoform 4 |
|  |  | Tripartite motif-containing protein 16 (cDNA FLJ31464 fis; clone NT2NE2001337; highly similar to Tripartite motif-containing protein 16) |
|  |  | Nucleolar protein 6 (Nucleolar RNA-associated protein) (Nrap) |
|  |  | Nuclear pore complex protein Nup88 (88 kDa nucleoporin) (Nucleoporin Nup88) |
|  |  | Sorting nexin 5 variant (Fragment) |
|  |  | Beta-1-syntrophin (59 kDa dystrophin-associated protein A1 basic component 1) (DAPA1B) (BSYN2) (Syntrophin-2) (Tax interaction protein 43) (TIP-43) |
|  |  | DnaJ homolog subfamily C member 10 (EC 1.8.4.-) (Endoplasmic reticulum DNA J domain-containing protein 5) (ER-resident protein ERdj5) (ERdj5) (Macrothioredoxin) (MTHr) |
|  |  | Coiled-coil domain containing 22; isoform CRA_a |
|  |  | Gamma-tubulin complex component 2 (GCP-2) (hGCP2) (Gamma-ring complex protein 103 kDa) (h103p) (hGrip103) (Spindle pole body protein Spc97 homolog) (hSpc97) |
|  |  | Golgi transport 1 homolog B (S. cerevisiae); isoform CRA_c (Vesicle transport protein GOT1B) |
|  |  | Guanine nucleotide binding protein |
|  |  | TNFRSF6 protein (Fragment) |
|  |  | Retinol dehydrogenase 10 (All-trans); isoform CRA_a |
|  |  | Protein transport protein SEC23 |
|  |  | Serpin peptidase inhibitor; clade B (Ovalbumin); member 9; isoform CRA_a (Testicular tissue protein Li 180) |
|  |  | cDNA; FLJ94074; highly similar to Homo sapiens caspase 1; apoptosis-related cysteine protease(interleukin 1; beta; convertase) (CASP1); transcript variantalpha; mRNA |
|  |  | Ubiquitin-like protein 4A |
|  |  | PRRC2A |
|  |  | Cation-transporting ATPase (EC 7.2.2.-) |
|  |  | cDNA FLJ51688; highly similar to Cleavage stimulation factor 50 kDa subunit |
|  |  | Splicing factor; arginine/serine-rich 15 |
|  |  | E3 ubiquitin-protein ligase KCMF1 (EC 2.3.2.27) (FGF-induced in gastric cancer) (Potassium channel modulatory factor) (PCMF) (RING-type E3 ubiquitin transferase KCMF1) (ZZ-type zinc finger-containing protein 1) |
|  |  | Echinoderm microtubule-associated protein-like 1 |
|  |  | Glycerol kinase (GK) (Glycerokinase) (EC 2.7.1.30) (ATP:glycerol 3-phosphotransferase) |
|  |  | RNA-binding protein fox-1 homolog 2 (Fragment) |
|  |  | SNW1 protein |
|  |  | NADH dehydrogenase 1 alpha subcomplex assembly factor 2 isoform 1 (Fragment) |
|  |  | GTPase-activating protein and VPS9 domain-containing protein 1 |
|  |  | cDNA FLJ58182; highly similar to Protein CYR61 |
|  |  | WD repeat-containing protein 26 |
|  |  | Mitochondrial peptide methionine sulfoxide reductase (EC 1.8.4.11) (Peptide-methionine (S)-S-oxide reductase) (Peptide Met(O) reductase) (Protein-methionine-S-oxide reductase) (PMSR) |
|  |  | Multivesicular body subunit 12A |
|  |  | cDNA FLJ50983; highly similar to Homo sapiens lysocardiolipin acyltransferase (LYCAT); transcript variant 1; mRNA |
|  |  | cDNA FLJ10529 fis; clone NT2RP2000965; highly similar to Targeting protein for Xklp2 |
|  |  | Ras-related protein Rab-22A (Rab-22) |
|  |  | cDNA FLJ75500; highly similar to Homo sapiens EH domain binding protein 1; mRNA |
|  |  | Serine/threonine-protein kinase 10 (EC 2.7.11.1) (Lymphocyte-oriented kinase) |
|  |  | Mitochondrial ribosomal protein L49; isoform CRA_b |
|  |  | TRPM8 channel-associated factor 1 (TRP channel-associated factor 1) |
|  |  | Tetratricopeptide repeat protein 9C (Fragment) |
|  |  | Glutaryl CoA dehydrogenase |
|  |  | cDNA FLJ75083; highly similar to Homo sapiens amine oxidase (flavin containing) domain 2 (AOF2);mRNA |
|  |  | cDNA FLJ58285; highly similar to Homo sapiens pre-B-cell leukemia transcription factor interacting protein 1 (PBXIP1); mRNA |
|  |  | Nucleolar protein 16 (HBV pre-S2 trans-regulated protein 3) |
|  |  | Thioredoxin-related transmembrane protein 4 (Thioredoxin domain-containing protein 13) |
|  |  | ATP synthase subunit gamma |
|  |  | CDC2L2 protein (Fragment) |
|  |  | Ubiquitin carboxyl-terminal hydrolase 15 (EC 3.4.19.12) (Deubiquitinating enzyme 15) (Ubiquitin thioesterase 15) (Ubiquitin-specific-processing protease 15) (Unph-2) (Unph4) |
|  |  | Chondrosarcoma-associated protein 2 |
|  |  | cDNA FLJ53837; moderately similar to Homo sapiens acyl-Coenzyme A binding domain containing 5 (ACBD5); mRNA |
|  |  | Transcriptional enhancer factor TEF-1 |
|  |  | Kinesin-like protein KIF13A |
|  |  |  |
| vi. | Common proteins in both 100 µM c-di-AMP and 100 µM c-di-GMP treated cells | Interferon-induced GTP-binding protein Mx1 (Interferon-induced protein p78) (IFI-78K) (Interferon-regulated resistance GTP-binding protein MxA) (Myxoma resistance protein 1) (Myxovirus resistance protein 1) [Cleaved into: Interferon-induced GTP-binding protein Mx1; N-terminally processed] |
|  |  | Interferon-induced protein with tetratricopeptide repeats 1 (IFIT-1) (Interferon-induced 56 kDa protein) (IFI-56K) (P56) |
|  |  | Interferon-induced protein with tetratricopeptide repeats 3; isoform CRA_a (Interferon-induced protein with tetratricopeptide repeats 3; isoform CRA_b) (cDNA FLJ75638; highly similar to Homo sapiens interferon-induced protein with tetratricopeptide repeats 3 (IFIT3); mRNA) |
|  |  | Ubiquitin/ISG15-conjugating enzyme E2 L6 (EC 2.3.2.23) (E2 ubiquitin-conjugating enzyme L6) (Retinoic acid-induced gene B protein) (RIG-B) (UbcH8) (Ubiquitin carrier protein L6) (Ubiquitin-protein ligase L6) |
|  |  | Alpha-endosulfine |
|  |  | cDNA FLJ75060; highly similar to Homo sapiens 2'-5'-oligoadenylate synthetase 2; 69/71kDa; mRNA |
|  |  | cDNA FLJ77542; highly similar to Homo sapiens YME1-like 1 (S. cerevisiae) (YME1L1); transcript variant 3; mRNA |

**Table S2.** List of upregulated proteins and their distribution in various groups following treatment with either 100 µM c-di-AMP or c-di-GMP

| S/No. | Treatment Group | Protein Name |
| --- | --- | --- |
| i. | Common proteins upregulated in both 100 µM c-di-AMP or 100µM c-di-GMP treated cells | Ubiquitin-like protein ISG15 (Interferon-induced 15 kDa protein) (Interferon-induced 17 kDa protein) (IP17) (Ubiquitin cross-reactive protein) (hUCRP) |
|  | | Deoxynucleoside triphosphate triphosphohydrolase SAMHD1 (dNTPase) (EC 3.1.5.-) (Dendritic cell-derived IFNG-induced protein) (DCIP) (Monocyte protein 5) (MOP-5) (SAM domain and HD domain-containing protein 1) (hSAMHD1) |
|  |  | MHC class I antigen |
|  |  | cDNA FLJ78682; highly similar to Homo sapiens 2'-5'-oligoadenylate synthetase 3; 100kDa (OAS3); mRNA |
|  |  | cDNA FLJ53509; highly similar to Galectin-3-binding protein |
|  |  | Signal transducer and activator of transcription |
|  |  | Signal transducer and activator of transcription 1-alpha/beta (Transcription factor ISGF-3 components p91/p84) |
|  |  | Methionine-R-sulfoxide reductase B3 (MsrB3) (EC 1.8.4.12) (EC 1.8.4.14) |
|  |  | E3 ubiquitin/ISG15 ligase TRIM25 (EC 6.3.2.n3) (Estrogen-responsive finger protein) (RING finger protein 147) (RING-type E3 ubiquitin transferase) (EC 2.3.2.27) (RING-type E3 ubiquitin transferase TRIM25) (Tripartite motif-containing protein 25) (Ubiquitin/ISG15-conjugating enzyme TRIM25)(Zinc finger protein147) |
|  |  | cDNA FLJ57805; highly similar to Homo sapiens paraspeckle component 1 (PSPC1); transcript variant alpha; mRNA |
|  |  | cDNA FLJ77542; highly similar to Homo sapiens YME1-like 1 (S. cerevisiae) (YME1L1); transcript variant 3; mRNA |
|  |  | Interferon-induced GTP-binding protein Mx1 (Interferon-induced protein p78) (IFI-78K) (Interferon-regulated resistance GTP-binding protein MxA) (Myxoma resistance protein 1) (Myxovirus resistance protein 1) [Cleaved into: Interferon-induced GTP-binding protein Mx1; N-terminally processed] |
|  |  | Interferon-induced protein with tetratricopeptide repeats 1 (IFIT-1) (Interferon-induced 56 kDa protein) (IFI-56K) (P56) |
|  |  | Interferon-induced protein with tetratricopeptide repeats 3; isoform CRA_a (Interferon-induced protein with tetratricopeptide repeats 3; isoform CRA_b) (cDNA FLJ75638; highly similar to Homo sapiens interferon-induced protein with tetratricopeptide repeats 3 (IFIT3); mRNA) |
|  |  | Ubiquitin/ISG15-conjugating enzyme E2 L6 (EC 2.3.2.23) (E2 ubiquitin-conjugating enzyme L6) (Retinoic acid-induced gene B protein) (RIG-B) (UbcH8) (Ubiquitin carrier protein L6) (Ubiquitin-protein ligase L6) |
|  |  | Alpha-endosulfine |
|  |  | cDNA FLJ75060; highly similar to Homo sapiens 2'-5'-oligoadenylate synthetase 2; 69/71kDa; mRNA |
|  |  |  |
| ii. | Proteins upregulated exclusively in 100 µM c-di-AMP treated cells | UBE2C protein (Ubiquitin-conjugating enzyme E2C) (Ubiquitin-conjugating enzyme E2C; isoform CRA_e) |
|  | | 40S ribosomal protein S27 |
|  |  | Adenosylhomocysteinase (EC 3.3.1.1) |
|  |  | DnaJ (Hsp40) homolog; subfamily B; member 4 variant (Fragment) |
|  |  | eIF2AK2 protein |
|  |  | RNA-binding protein EWS |
|  |  | Nuclear autoantigen Sp-100 (Fragment) |
|  |  | Interferon-induced 35 kDa protein (IFP 35) (Ifi-35) |
|  |  | cDNA FLJ60912; highly similar to Vinexin |
|  |  | Adenosine deaminase; RNA-specific isoform ADAR-a variant (Fragment) |
|  |  | SFRS11 protein (Fragment) |
|  |  | Slingshot homolog 3 (Drosophila); isoform CRA_f |
|  |  | Proteasome subunit beta (EC 3.4.25.1) |
|  |  | Fusion (Involved in t(12;16) in malignant liposarcoma) isoform a variant (Fragment) |
|  |  | Striatin; calmodulin binding protein 3 (cDNA FLJ77970; highly similar to Homo sapiens nuclear autoantigen GS2NA mRNA) |
|  |  | Phospholipase A-2-activating protein (PLA2P) (PLAP) |
|  |  | Lactamase; beta 2; isoform CRA_a (Testicular secretory protein Li 23) |
|  |  | Tropomyosin alpha-3 chain |
|  |  | Dynein heavy chain 3; axonemal (Axonemal beta dynein heavy chain 3) (HsADHC3) (Ciliary dynein heavy chain 3) (Dnahc3-b) |
|  |  | Interferon regulatory factor 9 (IRF-9) (IFN-alpha-responsive transcription factor subunit) (ISGF3 p48 subunit) (Interferon-stimulated gene factor 3 gamma) (ISGF-3 gamma) (Transcriptional regulator ISGF3 subunit gamma) |
|  |  | cDNA FLJ60546; highly similar to Homo sapiens bridging integrator 1 (BIN1); transcript variant 3; mRNA |
|  |  | Geranylgeranyl transferase type-2 subunit beta |
|  |  | Epidermal growth factor receptor kinase substrate 8-like protein 2 |
|  |  | Branched-chain-amino-acid aminotransferase (EC 2.6.1.42) |
|  |  | RNA binding motif protein 28 isoform 1 (RNA binding motif protein 28; isoform CRA_a) |
|  |  | ARHGAP35 (Fragment) |
|  |  | Inactive tyrosine-protein kinase PEAK1 (Pseudopodium-enriched atypical kinase 1) (Sugen kinase 269) (Tyrosine-protein kinase SgK269) |
|  |  | Nuclear cap-binding protein subunit 2 (20 kDa nuclear cap-binding protein) (Cell proliferation-inducing gene 55 protein) (NCBP 20 kDa subunit) (CBP20) (NCBP-interacting protein 1) (NIP1) |
|  |  | Histone deacetylase complex subunit SAP18 |
|  |  |  |
| iii. | Proteins upregulated exclusively in 100 µM c-di-GMP treated cells | Epididymis secretory sperm binding protein |
|  | | Plasminogen activator inhibitor 2 (PAI-2) (Monocyte Arg-serpin) (Placental plasminogen activator inhibitor) (Serpin B2) (Urokinase inhibitor) |
|  |  | Matrix metalloproteinase 1 preproprotein variant (Fragment) |
|  |  | Superoxide dismutase (EC 1.15.1.1) (Fragment) |
|  |  | Cell migration-inducing and hyaluronan-binding protein (EC 3.2.1.35) |
|  |  | Matrix metalloproteinase 14 (Membrane-inserted) |
|  |  | Thrombospondin-2 |
|  |  | cDNA FLJ34004 fis; clone FCBBF1000232; highly similar to Cytochrome P450 51A1 |
|  |  | cDNA FLJ59612; highly similar to Lactadherin |
|  |  | Cell proliferation-inducing protein 41 |
|  |  | Aldo-keto reductase family 1; member B1 (Aldose reductase); isoform CRA_a |
|  |  | Nuclear factor NF-kappa-B p100 subunit |
|  |  | Sequestosome-1 (EBI3-associated protein of 60 kDa) (EBIAP) (p60) (Phosphotyrosine-independent ligand for the Lck SH2 domain of 62 kDa) (Ubiquitin-binding protein p62) |
|  |  | EGF-like repeat and discoidin I-like domain-containing protein 3 (Developmentally-regulated endothelial cell locus 1 protein) (Integrin-binding protein DEL1) |
|  |  | cDNA FLJ57046; highly similar to Lysosomal alpha-glucosidase |
|  |  | Syndecan binding protein (Syntenin); isoform CRA_c |
|  |  | cDNA FLJ16785 fis; clone NT2RI2015342; highly similar to Solute carrier family 2; facilitated glucose transporter member 1 |
|  |  | COL6A2 protein (Collagen; type VI; alpha 2) |
|  |  | Thymidine phosphorylase (TP) (EC 2.4.2.4) (TdRPase) |
|  |  | Serpin peptidase inhibitor; clade E (Nexin; plasminogen activator inhibitor type 1); member 1; isoform CRA_b |
|  |  | Rho family GTPase 3; isoform CRA_b |
|  |  | Polyribonucleotide nucleotidyltransferase 1; mitochondrial (EC 2.7.7.8) (3'-5' RNA exonuclease OLD35) (PNPase old-35) (Polynucleotide phosphorylase 1) (PNPase 1) (Polynucleotide phosphorylase-like protein) |
|  |  | Ferritin |
|  |  | ENO2 protein (Enolase 2 (Gamma; neuronal); isoform CRA_a) (Epididymis secretory protein Li 279) (cDNA FLJ42665 fis; clone BRAMY2019989; highly similar to Gamma-enolase) (cDNA; FLJ92734; Homo sapiens enolase 2; (gamma; neuronal) (ENO2); mRNA) |
|  |  | cDNA FLJ51896; highly similar to Glia-derived nexin |
|  |  | Peroxidasin homolog (EC 1.11.1.7) (Melanoma-associated antigen MG50) (Vascular peroxidase 1) (p53-responsive gene 2 protein) |
|  |  | Phospholipase D family; member 3; isoform CRA_b |
|  |  | cDNA FLJ53367; highly similar to Transmembrane glycoprotein NMB |
|  |  | DNA-directed RNA polymerase II subunit RPB1 (RNA polymerase II subunit B1) (EC 2.7.7.6) (DNA-directed RNA polymerase II subunit A) (DNA-directed RNA polymerase III largest subunit) (RNA-directed RNA polymerase II subunit RPB1) (EC 2.7.7.48) |
|  |  | TAPBP |
|  |  | Metaxin-2 |
|  |  | Tumor necrosis factor receptor superfamily member 11B (Osteoclastogenesis inhibitory factor) (Osteoprotegerin) |
|  |  | Beta-2-microglobulin |
|  |  | Fibroblast growth factor (FGF) |
|  |  | Glucosidase; alpha acid (Pompe disease; glycogen storage disease type II) |
|  |  | cDNA FLJ61587; highly similar to Integrin alpha-1 (Fragment) |
|  |  | ADP-ribosylation factor GTPase-activating protein 1 (ARF GAP 1) (ADP-ribosylation factor 1 GTPase-activating protein) (ARF1 GAP) (ARF1-directed GTPase-activating protein) |
|  |  | A0A024R433 |
|  |  | Isopentenyl-diphosphate Delta-isomerase 1 (EC 5.3.3.2) (Isopentenyl pyrophosphate isomerase 1) (IPP isomerase 1) (IPPI1) |
|  |  | Thrombospondin-1 (Glycoprotein G) |
|  |  | IRGQ protein |
|  |  | Peptidylprolyl isomerase (EC 5.2.1.8) |
|  |  | Leupaxin |
|  |  | Nicotinamide phosphoribosyltransferase (NAmPRTase) (EC 2.4.2.12) |
|  |  | NADH-cytochrome b5 reductase 2 (b5R.2) (EC 1.6.2.2) |
|  |  | Amyloid-beta precursor protein |
|  |  | 39S ribosomal protein L53; mitochondrial (L53mt) (MRP-L53) (Mitochondrial large ribosomal subunit protein mL53) |
|  |  | 3-hydroxy-3-methylglutaryl coenzyme A synthase (HMG-CoA synthase) (EC 2.3.3.10) |
|  |  | Collagen triple helix repeat-containing protein 1 (Protein NMTC1) |
|  |  | H.sapiens ras-related Hrab4 protein |
|  |  | Neuropilin-1 variant (Fragment) |
|  |  | Membrane metallo-endopeptidase |
|  |  | 39S ribosomal protein L24; mitochondrial (L24mt) (MRP-L24) (Mitochondrial large ribosomal subunit protein uL24m) |
|  |  | Ubiquitin carboxyl-terminal hydrolase (EC 3.4.19.12) |
|  |  | Receptor-interacting serine/threonine-protein kinase 2 (EC 2.7.11.1) (Tyrosine-protein kinase RIPK2) |
|  |  | Thyroid receptor-interacting protein 11 (TR-interacting protein 11) (TRIP-11) (Clonal evolution-related gene on chromosome 14 protein) (Golgi-associated microtubule-binding protein 210) (GMAP-210) (Trip230) |
|  |  | Filamin A-interacting protein 1-like (130 kDa GPBP-interacting protein) (90 kDa GPBP-interacting protein) (Protein down-regulated in ovarian cancer 1) (DOC-1) |
|  |  | E3 ubiquitin-protein ligase DTX3L (EC 2.3.2.27) (B-lymphoma- and BAL-associated protein) (Protein deltex-3-like) (RING-type E3 ubiquitin transferase DTX3L) (Rhysin-2) (Rhysin2) |
|  |  | Cyclin G-associated kinase variant (Fragment) |
|  |  | Interferon-induced GTP-binding protein Mx2 (Interferon-regulated resistance GTP-binding protein MxB) (Myxovirus resistance protein 2) (p78-related protein) |

Table S3. Proteins significantly upregulated by 100 µM c-di-AMP with a measurable fold change (p ≤ 0.05 and Log2 fold change ≥ 0.5)

| S/No. | Protein ID | Gene name | Protein name | Log2 Fold Change 100 µM c-di-AMP vs Control | T-TEST p-value Control vs 100 µM c-di-AMP |
| --- | --- | --- | --- | --- | --- |
| 1 | P05161 | ISG15 G1P2 UCRP | Ubiquitin-like protein ISG15 (Interferon-induced 15 kDa protein) (Interferon-induced 17 kDa protein) (IP17) (Ubiquitin cross-reactive protein) (hUCRP) | 3.5332 | 0.01444651 |
| 2 | Q9Y3Z3 | SAMHD1 MOP5 | Deoxynucleoside triphosphate triphosphohydrolase SAMHD1 (dNTPase) (EC 3.1.5.-) (Dendritic cell-derived IFNG-induced protein) (DCIP) (Monocyte protein 5) (MOP-5) (SAM domain and HD domain-containing protein 1) (hSAMHD1) | 2.1304 | 8.51664E-05 |
| 3 | Q5TZN3 | UBE2C hCG_38372 | UBE2C protein (Ubiquitin-conjugating enzyme E2C) (Ubiquitin-conjugating enzyme E2C; isoform CRA_e) | 0.9208 | 0.05023356 |
| 4 | A8KA84 | A8KA84 | cDNA FLJ78682; highly similar to Homo sapiens 2'-5'-oligoadenylate synthetase 3; 100kDa (OAS3); mRNA | 0.9085 | 0.02146039 |
| 5 | Q8IXL7 | MSRB3 UNQ1965/PRO4487 | Methionine-R-sulfoxide reductase B3 (MsrB3) (EC 1.8.4.12) (EC 1.8.4.14) | 0.8833 | 0.000681395 |
| 6 | H0YMV8 | RPS27L | 40S ribosomal protein S27 | 0.7864 | 0.0381439 |
| 7 | P42224 | STAT1 | Signal transducer and activator of transcription 1-alpha/beta (Transcription factor ISGF-3 components p91/p84) | 0.7127 | 8.97734E-05 |
| 8 | A0A024R0A8 | AHCYL1 hCG_38162 | Adenosylhomocysteinase (EC 3.3.1.1) | 0.7103 | 0.01271575 |
| 9 | Q59E89 | Q59E89 | DnaJ (Hsp40) homolog; subfamily B; member 4 variant (Fragment) | 0.7078 | 0.02540102 |
| 10 | B7ZKK7 | EIF2AK2 | eIF2AK2 protein | 0.7009 | 5.30044E-06 |
| 11 | B4DWI8 | B4DWI8 | cDNA FLJ57805; highly similar to Homo sapiens paraspeckle component 1 (PSPC1); transcript variant alpha; mRNA | 0.6455 | 0.02293787 |
| 12 | A0A494C164 | STAT2 | Signal transducer and activator of transcription | 0.6352 | 0.002227359 |
| 13 | B0QYK0 | EWSR1 | RNA-binding protein EWS | 0.6132 | 0.01248256 |
| 14 | C9JBL0 | SP100 | Nuclear autoantigen Sp-100 (Fragment) | 0.6107 | 0.01373905 |
| 15 | P80217 | IFI35 IFP35 | Interferon-induced 35 kDa protein (IFP 35) (Ifi-35) | 0.593 | 0.03744187 |
| 16 | B4DVR4 | B4DVR4 | cDNA FLJ60912; highly similar to Vinexin | 0.588 | 0.01832094 |
| 17 | Q59EC0 | Q59EC0 | Adenosine deaminase; RNA-specific isoform ADAR-a variant (Fragment) | 0.5737 | 0.002072562 |
| 18 | Q05BU6 | SFRS11 | SFRS11 protein (Fragment) | 0.551 | 0.01794335 |
| 19 | A0A024R5J4 | SSH3 hCG_21061 | Slingshot homolog 3 (Drosophila); isoform CRA_f | 0.5482 | 0.02302436 |
| 20 | A2ACR1 | PSMB9 hCG_17510 | Proteasome subunit beta (EC 3.4.25.1) | 0.5417 | 0.003131649 |
| 21 | Q14258 | TRIM25 EFP RNF147 ZNF147 | E3 ubiquitin/ISG15 ligase TRIM25 (EC 6.3.2.n3) (Estrogen-responsive finger protein) (RING finger protein 147) (RING-type E3 ubiquitin transferase) (EC 2.3.2.27) (RING-type E3 ubiquitin transferase TRIM25) (Tripartite motif-containing protein 25) (Ubiquitin/ISG15-conjugating enzyme TRIM25) (Zinc finger protein 147) | 0.5201 | 0.00100788 |
| 22 | A0A1C3PHH3 | HLA-B | MHC class I antigen | 0.5158 | 0.02444818 |
| 23 | B4DI70 | B4DI70 | cDNA FLJ53509; highly similar to Galectin-3-binding protein | 0.5143 | 0.04110686 |
| 24 | Q59H57 | Q59H57 | Fusion (Involved in t(12;16) in malignant liposarcoma) isoform a variant (Fragment) | 0.5129 | 0.03525603 |
| 25 | A0AV58 | STRN3 | Striatin; calmodulin binding protein 3 (cDNA FLJ77970; highly similar to Homo sapiens nuclear autoantigen GS2NA mRNA) | 0.5001 | 0.02117329 |

**Table S4.** Proteins significantly upregulated by 100 µM c-di-GMP with a measurable fold change (p ≤ 0.05 and Log2 fold change ≥ 0.5)

| S/No. | Protein ID | Gene names | Protein names | Log2 Fold Change 100 µM c-di-GMP vs Control | T-TEST p-value Control vs 100 µM c-di-GMP |
| --- | --- | --- | --- | --- | --- |
| 1 | A0A384MEK5 | A0A384MEK5 | Epididymis secretory sperm binding protein | 4.2983 | 0.02941964 |
| 2 | P05120 | SERPINB2 PAI2 PLANH2 | Plasminogen activator inhibitor 2 (PAI-2) (Monocyte Arg-serpin) (Placental plasminogen activator inhibitor) (Serpin B2) (Urokinase inhibitor) | 3.6049 | 1.6405E-05 |
| 3 | P05161 | ISG15 G1P2 UCRP | Ubiquitin-like protein ISG15 (Interferon-induced 15 kDa protein) (Interferon-induced 17 kDa protein) (IP17) (Ubiquitin cross-reactive protein) (hUCRP) | 3.3245 | 0.01478059 |
| 4 | Q53G75 | Q53G75 | Matrix metalloproteinase 1 preproprotein variant (Fragment) | 2.6525 | 0.004700702 |
| 5 | Q9Y3Z3 | SAMHD1 MOP5 | Deoxynucleoside triphosphate triphosphohydrolase SAMHD1 (dNTPase) (EC 3.1.5.-) (Dendritic cell-derived IFNG-induced protein) (DCIP) (Monocyte protein 5) (MOP-5) (SAM domain and HD domain-containing protein 1) (hSAMHD1) | 2.2485 | 6.30328E-05 |
| 6 | Q7Z7M4 | SOD2 | Superoxide dismutase (EC 1.15.1.1) (Fragment) | 2.0754 | 6.81076E-07 |
| 7 | Q8WUJ3 | CEMIP KIAA1199 | Cell migration-inducing and hyaluronan-binding protein (EC 3.2.1.35) | 1.8803 | 0.004963125 |
| 8 | A0A1C3PHH3 | HLA-B | MHC class I antigen | 1.5498 | 0.001589588 |
| 9 | K4RH61 | MMP14 | Matrix metalloproteinase 14 (Membrane-inserted) | 1.5119 | 3.05612E-05 |
| 10 | A8KA84 |  | cDNA FLJ78682; highly similar to Homo sapiens 2'-5'-oligoadenylate synthetase 3; 100kDa (OAS3); mRNA | 1.5107 | 0.02084006 |
| 11 | A0A3B3ITK0 | THBS2 | Thrombospondin-2 | 1.3195 | 8.00383E-06 |
| 12 | B3KRC6 | B3KRC6 | cDNA FLJ34004 fis; clone FCBBF1000232; highly similar to Cytochrome P450 51A1 | 1.2891 | 0.005703806 |
| 13 | B4E396 | B4E396 | cDNA FLJ59612; highly similar to Lactadherin | 1.2762 | 1.05415E-05 |
| 14 | A1KY36 | A1KY36 | Cell proliferation-inducing protein 41 | 1.1396 | 0.00029749 |
| 15 | A0A024R7A8 | AKR1B1 hCG_20344 | Aldo-keto reductase family 1; member B1 (Aldose reductase); isoform CRA_a | 1.0901 | 4.44517E-05 |
| 16 | B4DI70 | B4DI70 | cDNA FLJ53509; highly similar to Galectin-3-binding protein | 1.0333 | 0.00555492 |
| 17 | A0A1P8C958 | NFKB2 | Nuclear factor NF-kappa-B p100 subunit | 1.0303 | 0.000289713 |
| 18 | Q13501 | SQSTM1 ORCA OSIL | Sequestosome-1 (EBI3-associated protein of 60 kDa) (EBIAP) (p60) (Phosphotyrosine-independent ligand for the Lck SH2 domain of 62 kDa) (Ubiquitin-binding protein p62) | 0.996 | 0.001932215 |
| 19 | O43854 | EDIL3 DEL1 | EGF-like repeat and discoidin I-like domain-containing protein 3 (Developmentally-regulated endothelial cell locus 1 protein) (Integrin-binding protein DEL1) | 0.9071 | 0.002563218 |
| 20 | B7Z5V6 | B7Z5V6 | cDNA FLJ57046; highly similar to Lysosomal alpha-glucosidase | 0.8915 | 0.000800577 |
| 21 | A0A024R7Z5 | SDCBP hCG_1787561 | Syndecan binding protein (Syntenin); isoform CRA_c | 0.8578 | 9.24097E-06 |
| 22 | B3KVN0 | B3KVN0 | cDNA FLJ16785 fis; clone NT2RI2015342; highly similar to Solute carrier family 2; facilitated glucose transporter member 1 | 0.8465 | 4.8619E-05 |
| 23 | Q9BUM6 | Q9BUM6 | COL6A2 protein (Collagen; type VI; alpha 2) | 0.8419 | 0.01185393 |
| 24 | A0A494C164 | STAT2 | Signal transducer and activator of transcription | 0.8403 | 0.000406579 |
| 25 | E5KRG5 | TYMP hCG_1988078 | Thymidine phosphorylase (TP) (EC 2.4.2.4) (TdRPase) | 0.8262 | 0.000277134 |
| 26 | A0A024QYT5 | SERPINE1 hCG_17353 | Serpin peptidase inhibitor; clade E (Nexin; plasminogen activator inhibitor type 1); member 1; isoform CRA_b | 0.8244 | 8.41572E-05 |
| 27 | D3DP96 | RND3 hCG_15792 | Rho family GTPase 3; isoform CRA_b | 0.8105 | 0.01209529 |
| 28 | Q8TCS8 | PNPT1 PNPASE | Polyribonucleotide nucleotidyltransferase 1; mitochondrial (EC 2.7.7.8) (3'-5' RNA exonuclease OLD35) (PNPase old-35) (Polynucleotide phosphorylase 1) (PNPase 1) (Polynucleotide phosphorylase-like protein) | 0.8052 | 0.02124857 |
| 29 | Q6NZ44 | FTH1 | Ferritin | 0.7917 | 0.003405452 |
| 30 | Q6FHV6 | ENO2 HEL-S-279 hCG_25937 | ENO2 protein (Enolase 2 (Gamma; neuronal); isoform CRA_a) (Epididymis secretory protein Li 279) (cDNA FLJ42665 fis; clone BRAMY2019989; highly similar to Gamma-enolase) (cDNA; FLJ92734; Homo sapiens enolase 2; (gamma; neuronal) (ENO2); mRNA) | 0.7801 | 0.001804969 |
| 31 | P42224 | STAT1 | Signal transducer and activator of transcription 1-alpha/beta (Transcription factor ISGF-3 components p91/p84) | 0.7602 | 4.29914E-05 |
| 32 | B4DMR3 | B4DMR3 | cDNA FLJ51896; highly similar to Glia-derived nexin | 0.7317 | 0.003910291 |
| 33 | Q92626 | PXDN KIAA0230 MG50 PRG2 VPO VPO1 | Peroxidasin homolog (EC 1.11.1.7) (Melanoma-associated antigen MG50) (Vascular peroxidase 1) (p53-responsive gene 2 protein) | 0.685 | 0.000170268 |
| 34 | A0A024R0Q4 | PLD3 hCG_1995506 | Phospholipase D family; member 3; isoform CRA_b | 0.6765 | 0.01423375 |
| 35 | B4DN79 | B4DN79 | cDNA FLJ53367; highly similar to Transmembrane glycoprotein NMB | 0.6731 | 0.01341297 |
| 36 | Q8IXL7 | MSRB3 UNQ1965/PRO4487 | Methionine-R-sulfoxide reductase B3 (MsrB3) (EC 1.8.4.12) (EC 1.8.4.14) | 0.641 | 0.01367194 |
| 37 | P24928 | POLR2A POLR2 | DNA-directed RNA polymerase II subunit RPB1 (RNA polymerase II subunit B1) (EC 2.7.7.6) (DNA-directed RNA polymerase II subunit A) (DNA-directed RNA polymerase III largest subunit) (RNA-directed RNA polymerase II subunit RPB1) (EC 2.7.7.48) | 0.6381 | 0.01546836 |
| 38 | A0A1U9X8L5 | A0A1U9X8L5 | TAPBP | 0.6318 | 0.01331544 |
| 39 | A0A1G4HQ05 | HLA-A | MHC class I antigen | 0.6174 | 0.03579318 |
| 40 | C9JNK6 | MTX2 | Metaxin-2 | 0.606 | 0.04964096 |
| 41 | O00300 | TNFRSF11B OCIF OPG | Tumor necrosis factor receptor superfamily member 11B (Osteoclastogenesis inhibitory factor) (Osteoprotegerin) | 0.6043 | 0.00796677 |
| 42 | B4E0X1 | B4E0X1 | Beta-2-microglobulin | 0.6036 | 0.00288001 |
| 43 | D9ZGF5 | FGF2 | Fibroblast growth factor (FGF) | 0.5986 | 0.007234615 |
| 44 | A0A384MDR3 | FTL hCG_39405 | Ferritin | 0.5831 | 0.006956723 |
| 45 | A0A024R8Q1 | GAA hCG_27920 | Glucosidase; alpha acid (Pompe disease; glycogen storage disease type II); isoform CRA_a | 0.5793 | 0.02444482 |
| 46 | B4DTY8 | B4DTY8 | cDNA FLJ61587; highly similar to Integrin alpha-1 (Fragment) | 0.5792 | 0.04231428 |
| 47 | Q14258 | TRIM25 EFP RNF147 ZNF147 | E3 ubiquitin/ISG15 ligase TRIM25 (EC 6.3.2.n3) (Estrogen-responsive finger protein) (RING finger protein 147) (RING-type E3 ubiquitin transferase) (EC 2.3.2.27) (RING-type E3 ubiquitin transferase TRIM25) (Tripartite motif-containing protein 25) (Ubiquitin/ISG15-conjugating enzyme TRIM25) (Zinc finger protein 147) | 0.5633 | 0.00213628 |
| 48 | Q8N6T3 | ARFGAP1 ARF1GAP | ADP-ribosylation factor GTPase-activating protein 1 (ARF GAP 1) (ADP-ribosylation factor 1 GTPase-activating protein) (ARF1 GAP) (ARF1-directed GTPase-activating protein) | 0.5632 | 0.04607984 |
| 49 | A0A024R433 | A0A024R433 |  | 0.5562 | 0.006499066 |
| 50 | Q13907 | IDI1 | Isopentenyl-diphosphate Delta-isomerase 1 (EC 5.3.3.2) (Isopentenyl pyrophosphate isomerase 1) (IPP isomerase 1) (IPPI1) | 0.5527 | 0.003628837 |
| 51 | B4DWI8 | B4DWI8 | cDNA FLJ57805; highly similar to Homo sapiens paraspeckle component 1 (PSPC1); transcript variant alpha; mRNA | 0.5483 | 0.01373859 |
| 52 | P07996 | THBS1 TSP TSP1 | Thrombospondin-1 (Glycoprotein G) | 0.5285 | 0.000191529 |
| 53 | B7ZMD6 | IRGQ | IRGQ protein | 0.5152 | 0.01673345 |
| 54 | F8VU90 | FKBP11 | Peptidylprolyl isomerase (EC 5.2.1.8) | 0.5123 | 0.009934408 |
| 55 | O60711 | LPXN LDLP | Leupaxin | 0.5028 | 0.004842474 |
| 56 | A0A024R718 | PBEF1 hCG_18025 | Nicotinamide phosphoribosyltransferase (NAmPRTase) (EC 2.4.2.12) | 0.4999 | 0.000130062 |

**Table S5.** List of downregulated proteins and their distribution in various groups following treatment with either 100 µM c-di-AMP or c-di-GMP

| S/No | Treatment Group | Protein Name |
| --- | --- | --- |
| i. | 50 Common proteins in 100 µM c-di-AMP and also in 100 µM c-di-GMP treated cells | Queuosine salvage protein |
|  | | cDNA FLJ55135; highly similar to Di-N-acetylchitobiase |
|  |  | Mannose-P-dolichol utilization defect 1 protein |
|  |  | 39S ribosomal protein L38; mitochondrial (L38mt) (MRP-L38) (Mitochondrial large ribosomal subunit protein mL38) |
|  |  | Carboxypeptidase (EC 3.4.16.-) |
|  |  | Phosphorylated adapter RNA export protein (RNA U small nuclear RNA export adapter protein) |
|  |  | A0A024R473 |
|  |  | Ribosome production factor 2 homolog (Fragment) |
|  |  | cDNA FLJ76707; highly similar to Homo sapiens transmembrane protein 87A; mRNA |
|  |  | cDNA; FLJ94423; highly similar to Homo sapiens mitochondrial ribosomal protein L23 (MRPL23); nuclear gene encoding mitochondrial protein; mRNA |
|  |  | V-type proton ATPase subunit |
|  |  | cDNA FLJ60304; highly similar to Rab GTPase-binding effector protein 1 |
|  |  | cDNA FLJ56289; highly similar to Homo sapiens sperm specific antigen 2 (SSFA2); mRNA |
|  |  | Apolipoprotein B mRNA editing enzyme catalytic polypeptide-like 3B (Apolipoprotein B mRNA editing enzyme; catalytic polypeptide-like 3B; isoform CRA_c) |
|  |  | Golgi-associated PDZ and coiled-coil motif-containing protein (CFTR-associated ligand) (Fused in glioblastoma) (PDZ protein interacting specifically with TC10) (PIST) |
|  |  | Pumilio homolog 1 (Fragment) |
|  |  | ADP-ribosylation factor-like protein 6-interacting protein 4 (Fragment) |
|  |  | Active breakpoint cluster region-related protein (cDNA FLJ54747; highly similar to Active breakpoint cluster region-related protein) |
|  |  | Non-specific serine/threonine protein kinase (EC 2.7.11.1) |
|  |  | cDNA FLJ37014 fis; clone BRACE2010203; highly similar to Zinc phosphodiesterase ELAC protein 2 |
|  |  | E3 ubiquitin-protein ligase MARCH5 (EC 2.3.2.27) (Membrane-associated RING finger protein 5) (Membrane-associated RING-CH protein V) (MARCH-V) (Mitochondrial ubiquitin ligase) (MITOL) (RING finger protein 153) (RING-type E3 ubiquitin transferase MARCH5) |
|  |  | Guanine nucleotide-binding protein-like 3 (E2-induced gene 3 protein) (Novel nucleolar protein 47) (NNP47) (Nucleolar GTP-binding protein 3) (Nucleostemin) |
|  |  | Retinal rod rhodopsin-sensitive cGMP 3';5'-cyclic phosphodiesterase subunit delta |
|  |  | Protein LBH |
|  |  | Gamma-tubulin complex component 3 (GCP-3) (hGCP3) (Gamma-ring complex protein 104 kDa) (h104p) (hGrip104) (Spindle pole body protein Spc98 homolog) (hSpc98) |
|  |  | cDNA; FLJ96555 |
|  |  | LIM and senescent cell antigen-like-containing domain protein 1 (Particularly interesting new Cys-His protein 1) (PINCH-1) (Renal carcinoma antigen NY-REN-48) |
|  |  | Transmembrane protein 205 (Fragment) |
|  |  | Tumor protein p53-inducible protein 11 (Fragment) |
|  |  | Processing of 1; ribonuclease P/MRP subunit (S. cerevisiae) |
|  |  | cDNA FLJ75682; highly similar to Homo sapiens armadillo repeat containing; X-linked 3 (ARMCX3); transcript variant 1; mRNA |
|  |  | cDNA FLJ60156; highly similar to Vacuolar protein sorting protein 52 |
|  |  | Retinol dehydrogenase 14 (All-trans and 9-cis); isoform CRA_a (cDNA; FLJ95921; Homo sapiens retinol dehydrogenase 14 (all-trans and 9-cis)(RDH14); mRNA) |
|  |  | C-Mpl binding protein (La-related protein 4) |
|  |  | Uridine-cytidine kinase 2 |
|  |  | Abl interactor 2 |
|  |  | Microtubule-associated proteins 1A/1B light chain 3A (Autophagy-related protein LC3 A) (Autophagy-related ubiquitin-like modifier LC3 A) (MAP1 light chain 3-like protein 1) (MAP1A/MAP1B light chain 3 A) (MAP1A/MAP1B LC3 A) (Microtubule-associated protein 1 light chain 3 alpha) |
|  |  | cDNA FLJ57941; highly similar to Nucleoside diphosphate-linked moiety X motif16 |
|  |  | CCR4-NOT transcription complex; subunit 3; isoform CRA_a |
|  |  | Sorting nexin-27 |
|  |  | Serine/threonine-protein kinase N2 (EC 2.7.11.13) (PKN gamma) (Protein kinase C-like 2) (Protein-kinase C-related kinase 2) |
|  |  | Large neutral amino acids transporter small subunit 1 (4F2 light chain) (4F2 LC) (4F2LC) (CD98 light chain) (Integral membrane protein E16) (E16) (L-type amino acid transporter 1) (hLAT1) (Solute carrier family 7 member 5) (y+ system cationic amino acid transporter) |
|  |  | cDNA; FLJ96877 |
|  |  | Zinc finger; NFX1-type containing 1; isoform CRA_b |
|  |  | cDNA FLJ52943; highly similar to Zinc transporter SLC39A7 |
|  |  | MHC class I antigen (Fragment) |
|  |  | Protein C10 |
|  |  | Sushi domain containing 2; isoform CRA_a (Testicular tissue protein Li 190) |
|  |  | 60S ribosomal protein L36a-like (Large ribosomal subunit protein eL42-like) |
|  |  | Transcriptional enhancer factor TEF-1 |
|  |  |  |
| ii. | Proteins included exclusively in 100 µM c-di-GMP treated cells | Mycophenolic acid acyl-glucuronide esterase; mitochondrial (EC 3.1.1.93) (Alpha/beta hydrolase domain-containing protein 10) (Abhydrolase domain-containing protein 10) |
|  | | Putative hydroxypyruvate isomerase (Fragment) |
|  |  | cDNA FLJ58014; highly similar to Homo sapiens programmed cell death 4; transcript variant 1; mRNA |
|  |  | cDNA FLJ61262; highly similar to Ubiquitin-protein ligase E3C |
|  |  | DEAD-box corepressor DP103 |
|  |  | RWD domain-containing protein 1 (Fragment) |
|  |  | Bumetanide-sensitive Na-K-Cl cotransporter (Solute carrier family 12 (Sodium/potassium/chloride transporters); member 2; isoform CRA_b) |
|  |  | Mitotic-spindle organizing protein 2B |
|  |  | Kinesin-like protein KIF20A (GG10_2) (Mitotic kinesin-like protein 2) (MKlp2) (Rab6-interacting kinesin-like protein) (Rabkinesin-6) |
|  |  | Serine threonine kinase 39 isoform B (Fragment) |
|  |  | Uncharacterized protein DKFZp547A0616 (Fragment) |
|  |  | Solute carrier family 25 member 4 isoform 1 (Fragment) |
|  |  | Protein regulator of cytokinesis 1; isoform CRA_e |
|  |  | Rab GTPase-activating protein 1 (GAP and centrosome-associated protein) (Rab6 GTPase-activating protein GAPCenA) |
|  |  | Tyrosine-protein kinase BAZ1B (EC 2.7.10.2) (Bromodomain adjacent to zinc finger domain protein 1B) (Williams syndrome transcription factor) (Williams-Beuren syndrome chromosomal region 10 protein) (Williams-Beuren syndrome chromosomal region 9 protein) (hWALp2) |
|  |  | ER lumen protein-retaining receptor 1 (KDEL endoplasmic reticulum protein retention receptor 1) (KDEL receptor 1) (Putative MAPK-activating protein PM23) |
|  |  | FYVE and coiled-coil domain-containing protein 1 (Zinc finger FYVE domain-containing protein 7) |
|  |  | cDNA FLJ56545; highly similar to ATP-dependent RNA helicase DDX50 |
|  |  | Tetratricopeptide repeat domain 1 variant (Fragment) |
|  |  | DIS3 |
|  |  | SGCB protein |
|  |  | Glycerol-3-phosphate phosphatase (G3PP) (EC 3.1.3.21) (Aspartate-based ubiquitous Mg(2+)-dependent phosphatase) (AUM) (EC 3.1.3.48) (Phosphoglycolate phosphatase) (PGP) |
|  |  | Replication factor C subunit 2 (Activator 1 40 kDa subunit) (A1 40 kDa subunit) (Activator 1 subunit 2) (Replication factor C 40 kDa subunit) (RF-C 40 kDa subunit) (RFC40) |
|  |  | cDNA FLJ57657; highly similar to Charged multivesicular body protein 1a |
|  |  | Histone-lysine N-methyltransferase SETD7 (EC 2.1.1.-) (Histone H3-K4 methyltransferase SETD7) (H3-K4-HMTase SETD7) (Lysine N-methyltransferase 7) (SET domain-containing protein 7) (SET7/9) |
|  |  | Vacuolar-sorting protein SNF8 (ELL-associated protein of 30 kDa) (ESCRT-II complex subunit VPS22) (hVps22) |
|  |  | Glycylpeptide N-tetradecanoyltransferase (EC 2.3.1.97) |
|  |  | AP2-associated protein kinase 1 (Fragment) |
|  |  | Dihydrofolate reductase (EC 1.5.1.3) (cDNA; FLJ93028; Homo sapiens dihydrofolate reductase (DHFR); mRNA) |
|  |  | Ankyrin-1 (ANK-1) (Ankyrin-R) (Erythrocyte ankyrin) |
|  |  | TBC1 domain family member 23 (HCV non-structural protein 4A-transactivated protein 1) |
|  |  | Elongation factor Ts; mitochondrial (EF-Ts) (EF-TsMt) |
|  |  | UBX domain-containing protein 6 (UBX domain-containing protein 1) |
|  |  | NFU1 iron-sulfur cluster scaffold homolog; mitochondrial (HIRA-interacting protein 5) |
|  |  | Anoctamin-6 (Small-conductance calcium-activated nonselective cation channel) (SCAN channel) (Transmembrane protein 16F) |
|  |  | Nuclear pore complex protein Nup214 |
|  |  | Neurabin-2 (Neurabin-II) (Protein phosphatase 1 regulatory subunit 9B) (Spinophilin) |
|  |  | Protein diaphanous homolog 3 (Diaphanous-related formin-3) (DRF3) (MDia2) |
|  |  | Protein MEMO1 (C21orf19-like protein) (Hepatitis C virus NS5A-transactivated protein 7) (HCV NS5A-transactivated protein 7) (Mediator of ErbB2-driven cell motility 1) (Mediator of cell motility 1) (Memo-1) |
|  |  | Chromosome 11 open reading frame 58 |
|  |  | Beta-4 tubulin (Fragment) |
|  |  | cDNA FLJ42854 fis; clone BRHIP2008607; highly similar to Breast cancer anti-estrogen resistance protein 1 |
|  |  | Cytochrome b-c1 complex subunit 9 (Complex III subunit 9) (Complex III subunit X) (Cytochrome c1 non-heme 7 kDa protein) (Ubiquinol-cytochrome c reductase complex 7.2 kDa protein) |
|  |  | GPI ethanolamine phosphate transferase 1 |
|  |  | Eukaryotic translation elongation factor 1 alpha (Fragment) |
|  |  | Telomere length regulation protein TEL2 homolog (Protein clk-2 homolog) (hCLK2) |
|  |  | Nuclear pore complex protein Nup85 (85 kDa nucleoporin) (FROUNT) (Nucleoporin Nup75) (Nucleoporin Nup85) (Pericentrin-1) |
|  |  | 28S ribosomal protein S17; mitochondrial (Fragment) |
|  |  | 39S ribosomal protein L13; mitochondrial (L13mt) (MRP-L13) (Mitochondrial large ribosomal subunit protein uL13m) |
|  |  | Methyltransferase-like 26 |
|  |  | A0A024R313 |
|  |  | [F-actin]-monooxygenase MICAL2 |
|  |  | Starch-binding domain-containing protein 1 (Genethonin-1) (Glycophagy cargo receptor STBD1) |
|  |  | Transforming acidic coiled-coil-containing protein 3 |
|  |  | cDNA FLJ50501; highly similar to GRAM domain-containing protein 3 |
|  |  | NADH dehydrogenase (Ubiquinone) 1 alpha subcomplex; 3; 9kDa; isoform CRA_e (NADH dehydrogenase [ubiquinone] 1 alpha subcomplex subunit 3) (NDUFA3 protein) (cDNA FLJ76508; highly similar to Homo sapiens NADH dehydrogenase (ubiquinone) 1 alpha subcomplex; 3; 9kDa (NDUFA3); mRNA) |
|  |  | cAMP-regulated phosphoprotein 19 |
|  |  | Chromosome-associated kinesin KIF4A (Chromokinesin-A) |
|  |  | Zinc finger RNA-binding protein (hZFR) (M-phase phosphoprotein homolog) |
|  |  | cDNA FLJ90619 fis; clone PLACE1002374; highly similar to Cathepsin L |
|  |  | Cathepsin Z (EC 3.4.18.1) (Cathepsin P) (Cathepsin X) |
|  |  | Ubiquitin-fold modifier-conjugating enzyme 1 (Ufm1-conjugating enzyme 1) |
|  |  | Collagen alpha-1(I) chain (Alpha-1 type I collagen) |
|  |  | Retinol dehydrogenase 10 (All-trans); isoform CRA_a |
|  |  | 60S ribosomal protein L18a |
|  |  | Uveal autoantigen with coiled-coil domains and ankyrin repeats |
|  |  | Collagen; type I; alpha 2; isoform CRA_c (Epididymis secretory sperm binding protein) |
|  |  | Tyrosine 3-monooxygenase/tryptophan 5-monooxygenase activation protein; eta polypeptide; isoform CRA_b |
|  |  | Structural maintenance of chromosomes protein 4 (SMC protein 4) (SMC-4) (Chromosome-associated polypeptide C) (hCAP-C) (XCAP-C homolog) |
|  |  | Collagen alpha-1(III) chain |
|  |  | DNA mismatch repair protein (Fragment) |
|  |  | cDNA; FLJ93949; highly similar to Homo sapiens NIMA (never in mitosis gene a)-related kinase 7 (NEK7); mRNA |
|  |  | Kinetochore protein Spc24 (hSpc24) |
|  |  |  |
| iii. | Proteins included exclusively in 100 µM c-di-AMP treated cells | Syndecan binding protein (Syntenin); isoform CRA_c |
|  | | Plasminogen activator inhibitor 2 (PAI-2) (Monocyte Arg-serpin) (Placental plasminogen activator inhibitor) (Serpin B2) (Urokinase inhibitor) |
|  |  | Translocase of outer mitochondrial membrane 20 homolog (Yeast); isoform CRA_a |
|  |  | Sec61 alpha 1 subunit (S. cerevisiae); isoform CRA_c (cDNA FLJ14517 fis; clone NT2RM1000833; highly similar to Protein transport protein Sec61 subunit alpha isoform 1) (cDNA FLJ90225 fis; clone NT2RM1000855; highly similar to Protein transport protein Sec61 subunit alpha isoform 1) |
|  |  | Acetolactate synthase-like protein (EC 2.2.1.-) (IlvB-like protein) |
|  |  | 28S ribosomal protein S23; mitochondrial |
|  |  | cDNA FLJ53837; moderately similar to Homo sapiens acyl-Coenzyme A binding domain containing 5 (ACBD5); mRNA |
|  |  | CGI-111 protein |
|  |  | 3-hydroxyisobutyryl-CoA hydrolase; mitochondrial (EC 3.1.2.4) (3-hydroxyisobutyryl-coenzyme A hydrolase) |
|  |  | Sorting nexin-4 |
|  |  | Aldehyde dehydrogenase 1 family member L1 isoform 3 (Fragment) |
|  |  | MRPS9 protein (Fragment) |
|  |  | Mitochondrial ribosomal protein L49; isoform CRA_b |
|  |  | Glutaryl CoA dehydrogenase |
|  |  | Cytidine 5'-monophosphate N-acetylneuraminic acid synthetase variant (Fragment) |
|  |  | cDNA FLJ75725; highly similar to Homo sapiens vesicle transport through interaction with t-SNAREs homolog 1B (yeast) (VTI1B); mRNA |
|  |  | Fibronectin type III domain containing 3A; isoform CRA_b |
|  |  | cDNA FLJ58182; highly similar to Protein CYR61 |
|  |  | Glycerol kinase (GK) (Glycerokinase) (EC 2.7.1.30) (ATP:glycerol 3-phosphotransferase) |
|  |  | Protein ABHD11 (EC 3.-.-.-) (Alpha/beta hydrolase domain-containing protein 11) (Abhydrolase domain-containing protein 11) (Williams-Beuren syndrome chromosomal region 21 protein) |
|  |  | WD repeat-containing protein 26 |
|  |  | Nesprin-3 (KASH domain-containing protein 3) (KASH3) (Nuclear envelope spectrin repeat protein 3) |
|  |  | Mitochondrial ribosomal protein S7; isoform CRA_a |
|  |  | cDNA FLJ50791; highly similar to Nitrilase homolog 1 |
|  |  | Lysophosphatidylserine lipase ABHD12 (EC 3.1.-.-) (2-arachidonoylglycerol hydrolase ABHD12) (Abhydrolase domain-containing protein 12) (hABHD12) (Monoacylglycerol lipase ABHD12) (EC 3.1.1.23) (Oxidized phosphatidylserine lipase ABHD12) (EC 3.1.-.-) |
|  |  | cDNA FLJ31479 fis; clone NT2NE2001634; moderately similar to NADH-UBIQUINONE OXIDOREDUCTASE 9 KD SUBUNIT |
|  |  | IL6ST isoform 4 |
|  |  | Multivesicular body subunit 12A |
|  |  | Serpin peptidase inhibitor; clade B (Ovalbumin); member 9; isoform CRA_a (Testicular tissue protein Li 180) |
|  |  | Endothelial protein C receptor (Fragment) |
|  |  | Mitogen-activated protein kinase (EC 2.7.11.24) |
|  |  | MICOS complex subunit |
|  |  | cDNA FLJ54538; highly similar to Transportin-2 |
|  |  | NADH dehydrogenase (Ubiquinone) 1 beta subcomplex; 3; 12kDa; isoform CRA_a |
|  |  | Alpha-mannosidase (EC 3.2.1.-) |
|  |  | Chromosome 10 open reading frame 70; isoform CRA_b |
|  |  | E3 ubiquitin-protein ligase KCMF1 (EC 2.3.2.27) (FGF-induced in gastric cancer) (Potassium channel modulatory factor) (PCMF) (RING-type E3 ubiquitin transferase KCMF1) (ZZ-type zinc finger-containing protein 1) |
|  |  | Ras-related GTP binding C; isoform CRA_a |
|  |  | Mitochondrial import inner membrane translocase subunit Tim23 |
|  |  | SNW1 protein |
|  |  | Mitochondrial peptide methionine sulfoxide reductase (EC 1.8.4.11) (Peptide-methionine (S)-S-oxide reductase) (Peptide Met(O) reductase) (Protein-methionine-S-oxide reductase) (PMSR) |
|  |  | ER membrane protein complex subunit 8 (Fragment) |
|  |  | ADP-ribosylation factor GTPase-activating protein 2 (Fragment) |
|  |  | Protein transport protein SEC23 |
|  |  | Ectonucleoside triphosphate diphosphohydrolase 1 (NTPDase 1) (EC 3.6.1.5) (Ecto-ATP diphosphohydrolase 1) (Ecto-ATPDase 1) (Ecto-ATPase 1) (Ecto-apyrase) (Lymphoid cell activation antigen) (CD antigen CD39) |
|  |  | Translocase of inner mitochondrial membrane 9 homolog (Yeast); isoform CRA_a |
|  |  | Cation-transporting ATPase (EC 7.2.2.-) |
|  |  | Coiled-coil domain containing 22; isoform CRA_a |
|  |  | Ribosome biogenesis regulatory protein homolog |
|  |  | Matrix-remodeling-associated protein 7 |
|  |  | Ribosome biogenesis protein WDR12 (WD repeat-containing protein 12) |
|  |  | cDNA FLJ46798 fis; clone TRACH3031660; highly similar to cAMP-dependent protein kinase type II-beta regulatory subunit |
|  |  | cDNA FLJ78024 |
|  |  | cDNA FLJ61635; highly similar to Homo sapiens likely ortholog of mouse immediate early response; erythropoietin 4 (LEREPO4); mRNA |
|  |  | Phosphofurin acidic cluster sorting protein 1; isoform CRA_a |
|  |  | Diacylglycerol kinase (DAG kinase) (EC 2.7.1.107) |
|  |  | ADP-ribosylation factor GTPase-activating protein 1 (ARF GAP 1) (ADP-ribosylation factor 1 GTPase-activating protein) (ARF1 GAP) (ARF1-directed GTPase-activating protein) |
|  |  | V-type proton ATPase subunit a |
|  |  | Cleft lip and palate associated transmembrane protein 1 isoform 2 (Cleft lip and palate associated transmembrane protein 1; isoform CRA_b) |
|  |  | Selenoprotein F |
|  |  | N(4)-(beta-N-acetylglucosaminyl)-L-asparaginase (EC 3.5.1.26) (Aspartylglucosaminidase) (Glycosylasparaginase) (N4-(N-acetyl-beta-glucosaminyl)-L-asparagine amidase) [Cleaved into: Glycosylasparaginase alpha chain; Glycosylasparaginase beta chain] |
|  |  | Metaxin-2 |
|  |  | FLJ00144 protein (Fragment) |
|  |  | 60S ribosomal protein L22-like 1 |
|  |  | cDNA FLJ51641; highly similar to Nicastrin |
|  |  | cDNA FLJ61541; highly similar to Homo sapiens PDZ and LIM domain 5 (PDLIM5); transcript variant 2; mRNA |
|  |  | Chromobox homolog 5 (HP1 alpha homolog; Drosophila); isoform CRA_b (Epididymis luminal protein 25) |
|  |  | Contactin-associated protein 1 (Caspr) (Caspr1) (Neurexin IV) (Neurexin-4) (p190) |
|  |  | Mothers against decapentaplegic homolog (MAD homolog) (Mothers against DPP homolog) (SMAD family member) (Fragment) |
|  |  | E3 ubiquitin-protein ligase RNF181 |
|  |  | Frequenin homolog (Drosophila); isoform CRA_a |
|  |  | Uncharacterized protein DKFZp686E23276 (Fragment) |
|  |  | RUN and FYVE domain-containing protein 1 (FYVE-finger protein EIP1) (La-binding protein 1) (Rab4-interacting protein) (Zinc finger FYVE domain-containing protein 12) |
|  |  | EH domain-binding protein 1-like protein 1 |
|  |  | cDNA FLJ56673; highly similar to Homo sapiens adipocyte-specific adhesion molecule (ASAM); mRNA |
|  |  | Ras-related protein Rab-22A (Rab-22) |
|  |  | Torsin-1B (Torsin ATPase-1B) (EC 3.6.4.-) (Torsin family 1 member B) |
|  |  | cDNA FLJ50983; highly similar to Homo sapiens lysocardiolipin acyltransferase (LYCAT); transcript variant 1; mRNA |
|  |  | cDNA FLJ75882; highly similar to Homo sapiens spastic paraplegia 20; spartin (Troyer syndrome) (SPG20); mRNA |
|  |  | Lysophospholipid acyltransferase 7 (Fragment) |
|  |  | Core-binding factor; beta subunit; isoform CRA_b |
|  |  | cDNA FLJ58024; highly similar to NADH-ubiquinone oxidoreductase 20 kDa subunit; mitochondrial |
|  |  | Neuronal protein |
|  |  | Ribulose-phosphate 3-epimerase (EC 5.1.3.1) (Ribulose-5-phosphate-3-epimerase) |
|  |  | Protein PRR14L (Proline rich 14-like protein) |
|  |  | Translocon-associated protein subunit gamma |

**Table S6**. Proteins significantly downregulated by 100 µM c-di-AMP with a measurable fold change (p ≤ 0.05 and Log2 fold change ≥ -0.5)

| S/No | Protein ID | Gene name | Protein name | Log2 Fold Change 100 µM c-di-AMP vs Control | T-TEST p-value Control vs 100 µM c-di-AMP |
| --- | --- | --- | --- | --- | --- |
| 1 | P05120 | SERPINB2 PAI2 PLANH2 | Plasminogen activator inhibitor 2 (PAI-2) (Monocyte Arg-serpin) (Placental plasminogen activator inhibitor) (Serpin B2) (Urokinase inhibitor) | -0.9590 | 0.005166683 |
| 2 | B3KNF6 | SEC61A1 hCG_40108 | Sec61 alpha 1 subunit (S. cerevisiae); isoform CRA_c (cDNA FLJ14517 fis; clone NT2RM1000833; highly similar to Protein transport protein Sec61 subunit alpha isoform 1) (cDNA FLJ90225 fis; clone NT2RM1000855; highly similar to Protein transport protein Sec61 subunit alpha isoform 1) | -0.5713 | 0.007176389 |
| 3 | A0A024R3W2 | TOMM20 hCG_1810880 | Translocase of outer mitochondrial membrane 20 homolog (Yeast); isoform CRA_a | -0.5187 | 0.005670565 |
| 4 | A1L0T0 | ILVBL AHAS | Acetolactate synthase-like protein (EC 2.2.1.-) (IlvB-like protein) | -0.5125 | 0.04425698 |
| 5 | A0A024R7Z5 | SDCBP hCG_1787561 | Syndecan binding protein (Syntenin); isoform CRA_c | -0.4999 | 0.001796354 |

**Table S7.** Proteins significantly downregulated by 100 µM c-di-GMP with a measurable fold change (p ≤ 0.05 and Log2 fold change ≥ -0.5)

| S/No. | Protein ID | Gene name | Protein name | Log2 Fold Change 100 µM c-di-GMP vs Control | T-TEST p-value Control vs 100 µM c-di-GMP |
| --- | --- | --- | --- | --- | --- |
| 1 | Q96KR1 | ZFR | Zinc finger RNA-binding protein (hZFR) (M-phase phosphoprotein homolog) | -0.9341 | 0.03867683 |
| 2 | B3KQK4 |  | cDNA FLJ90619 fis; clone PLACE1002374; highly similar to Cathepsin L | -0.8909 | 0.00035668 |
| 3 | H0YE88 | TEAD1 | Transcriptional enhancer factor TEF-1 | -0.7378 | 0.00603228 |
| 4 | Q9UBR2 | CTSZ | Cathepsin Z (EC 3.4.18.1) (Cathepsin P) (Cathepsin X) | -0.7174 | 4.1484E-05 |
| 5 | Q9Y3C8 | UFC1 CGI-126 HSPC155 | Ubiquitin-fold modifier-conjugating enzyme 1 (Ufm1-conjugating enzyme 1) | -0.6356 | 0.04477089 |
| 6 | P02452 | COL1A1 | Collagen alpha-1(I) chain (Alpha-1 type I collagen) | -0.6113 | 0.00179866 |
| 7 | A0A024R7X6 | RDH10 hCG_19185 | Retinol dehydrogenase 10 (All-trans); isoform CRA_a | -0.5801 | 0.032918 |
| 8 | M0R117 | RPL18A | 60S ribosomal protein L18a | -0.5391 | 0.03784083 |
| 9 | B7ZKM6 | UACA | Uveal autoantigen with coiled-coil domains and ankyrin repeats | -0.5335 | 0.00287662 |
| 10 | A0A384MDU2 | COL1A2 hCG_1686428 | Collagen; type I; alpha 2; isoform CRA_c (Epididymis secretory sperm binding protein) | -0.5332 | 0.01235516 |
| 11 | A0A024R1K7 | YWHAH hCG_41511 | Tyrosine 3-monooxygenase/tryptophan 5-monooxygenase activation protein; eta polypeptide; isoform CRA_b | -0.5207 | 0.04579311 |
| 12 | Q9NTJ3 | SMC4 CAPC SMC4L1 | Structural maintenance of chromosomes protein 4 (SMC protein 4) (SMC-4) (Chromosome-associated polypeptide C) (hCAP-C) (XCAP-C homolog) | -0.5202 | 0.00054018 |
| 13 | P02461 | COL3A1 | Collagen alpha-1(III) chain | -0.4965 | 0.00100613 |

**Table S8.** Ingenuity pathways exclusively regulated by c-di-AMP

| S/No. | 10 elements included exclusively in "c-di-AMP Pathway": |
| --- | --- |
| 1 | Lipid Antigen Presentation by CD1 |
| 2 | Proline Biosynthesis I |
| 3 | IL-15 Production |
| 4 | Spliceosomal Cycle |
| 5 | Proline Biosynthesis II (from Arginine) |
| 6 | Arginine Degradation VI (Arginase 2 Pathway) |
| 7 | Salvage Pathways of Pyrimidine Deoxyribonucleotides |
| 8 | Sphingomyelin Metabolism |
| 9 | Leucine Degradation I |
| 10 | Dolichyl-diphosphooligosaccharide Biosynthesis |

**Table S9.** Ingenuity pathways exclusively regulated by c-di-GMP

| S/No. | 128 elements included exclusively in "c-di-GMP Pathway": |
| --- | --- |
| 1 | Hepatic Fibrosis Signaling Pathway |
| 2 | Dendritic Cell Maturation |
| 3 | Tumor Microenvironment Pathway |
| 4 | Glycolysis I |
| 5 | Hepatic Fibrosis / Hepatic Stellate Cell Activation |
| 6 | Neuroinflammation Signaling Pathway |
| 7 | Type I Diabetes Mellitus Signaling |
| 8 | Neuroprotective Role of THOP1 in Alzheimer's Disease |
| 9 | Gluconeogenesis I |
| 10 | Ferroptosis Signaling Pathway |
| 11 | Mitochondrial Dysfunction |
| 12 | Acute Phase Response Signaling |
| 13 | Sirtuin Signaling Pathway |
| 14 | Cytotoxic T Lymphocyte-mediated Apoptosis of Target Cells |
| 15 | T Helper Cell Differentiation |
| 16 | Inflammasome pathway |
| 17 | IL-6 Signaling |
| 18 | Myc Mediated Apoptosis Signaling |
| 19 | NRF2-mediated Oxidative Stress Response |
| 20 | Tumoricidal Function of Hepatic Natural Killer Cells |
| 21 | Natural Killer Cell Signaling |
| 22 | UVA-Induced MAPK Signaling |
| 23 | Apelin Liver Signaling Pathway |
| 24 | Apoptosis Signaling |
| 25 | Virus Entry via Endocytic Pathways |
| 26 | Induction of Apoptosis by HIV1 |
| 27 | HOTAIR Regulatory Pathway |
| 28 | HMGB1 Signaling |
| 29 | Retinoate Biosynthesis I |
| 30 | TWEAK Signaling |
| 31 | Docosahexaenoic Acid (DHA) Signaling |
| 32 | Atherosclerosis Signaling |
| 33 | JAK/Stat Signaling |
| 34 | Colorectal Cancer Metastasis Signaling |
| 35 | Intrinsic Prothrombin Activation Pathway |
| 36 | PTEN Signaling |
| 37 | Oncostatin M Signaling |
| 38 | BAG2 Signaling Pathway |
| 39 | IL-8 Signaling |
| 40 | Ceramide Signaling |
| 41 | Phospholipase C Signaling |
| 42 | Choline Biosynthesis III |
| 43 | Death Receptor Signaling |
| 44 | Cardiac Hypertrophy Signaling (Enhanced) |
| 45 | Senescence Pathway |
| 46 | mTOR Signaling |
| 47 | Granzyme B Signaling |
| 48 | TNFR1 Signaling |
| 49 | Bladder Cancer Signaling |
| 50 | GŒ±q Signaling |
| 51 | CD27 Signaling in Lymphocytes |
| 52 | FAT10 Signaling Pathway |
| 53 | Actin Cytoskeleton Signaling |
| 54 | PPAR Signaling |
| 55 | Methylglyoxal Degradation III |
| 56 | CNTF Signaling |
| 57 | The Visual Cycle |
| 58 | Pancreatic Adenocarcinoma Signaling |
| 59 | Antioxidant Action of Vitamin C |
| 60 | Oxidative Phosphorylation |
| 61 | Maturity Onset Diabetes of Young (MODY) Signaling |
| 62 | Tec Kinase Signaling |
| 63 | Role of Macrophages, Fibroblasts and Endothelial Cells in Rheumatoid Arthritis |
| 64 | Apelin Endothelial Signaling Pathway |
| 65 | Sphingosine-1-phosphate Signaling |
| 66 | NF-Œ∫B Signaling |
| 67 | Renin-Angiotensin Signaling |
| 68 | Molecular Mechanisms of Cancer |
| 69 | Role of NFAT in Regulation of the Immune Response |
| 70 | Phospholipases |
| 71 | Signaling by Rho Family GTPases |
| 72 | PI3K/AKT Signaling |
| 73 | Rac Signaling |
| 74 | Estrogen Receptor Signaling |
| 75 | Th1 Pathway |
| 76 | RhoA Signaling |
| 77 | Role of JAK family kinases in IL-6-type Cytokine Signaling |
| 78 | Axonal Guidance Signaling |
| 79 | Endothelin-1 Signaling |
| 80 | Systemic Lupus Erythematosus In T Cell Signaling Pathway |
| 81 | GP6 Signaling Pathway |
| 82 | PPARŒ±/RXRŒ± Activation |
| 83 | IL-10 Signaling |
| 84 | 14-3-3-mediated Signaling |
| 85 | RAR Activation |
| 86 | ERK5 Signaling |
| 87 | Caveolar-mediated Endocytosis Signaling |
| 88 | Regulation of Cellular Mechanics by Calpain Protease |
| 89 | TREM1 Signaling |
| 90 | Role of MAPK Signaling in Inhibiting the Pathogenesis of Influenza |
| 91 | ERK/MAPK Signaling |
| 92 | NF-Œ∫B Activation by Viruses |
| 93 | STAT3 Pathway |
| 94 | Toll-like Receptor Signaling |
| 95 | HIF1Œ± Signaling |
| 96 | NADH Repair |
| 97 | FLT3 Signaling in Hematopoietic Progenitor Cells |
| 98 | Actin Nucleation by ARP-WASP Complex |
| 99 | Xenobiotic Metabolism General Signaling Pathway |
| 100 | PEDF Signaling |
| 101 | IL-9 Signaling |
| 102 | Inhibition of Angiogenesis by TSP1 |
| 103 | IL-4 Signaling |
| 104 | Glucocorticoid Receptor Signaling |
| 105 | Osteoarthritis Pathway |
| 106 | B Cell Development |
| 107 | Acute Myeloid Leukemia Signaling |
| 108 | Crosstalk between Dendritic Cells and Natural Killer Cells |
| 109 | Altered T Cell and B Cell Signaling in Rheumatoid Arthritis |
| 110 | OX40 Signaling Pathway |
| 111 | PKCŒ∏ Signaling in T Lymphocytes |
| 112 | Inhibition of Matrix Metalloproteases |
| 113 | FcŒ≥ Receptor-mediated Phagocytosis in Macrophages and Monocytes |
| 114 | Creatine-phosphate Biosynthesis |
| 115 | Trans, trans-farnesyl Diphosphate Biosynthesis |
| 116 | dTMP De Novo Biosynthesis |
| 117 | Huntington's Disease Signaling |
| 118 | Regulation of eIF4 and p70S6K Signaling |
| 119 | Ceramide Biosynthesis |
| 120 | UDP-N-acetyl-D-glucosamine Biosynthesis II |
| 121 | Rapoport-Luebering Glycolytic Shunt |
| 122 | NAD Biosynthesis III |
| 123 | Th1 and Th2 Activation Pathway |
| 124 | SAPK/JNK Signaling |
| 125 | Role of RIG1-like Receptors in Antiviral Innate Immunity |
| 126 | Retinol Biosynthesis |
| 127 | Granulocyte Adhesion and Diapedesis |
| 128 | iNOS Signaling |
